# Solvent-Directed Social Chiral Self-Sorting in Pd_2_L_4_ Coordination Cages

**DOI:** 10.1021/jacs.4c12525

**Published:** 2024-11-17

**Authors:** Alexandre Walther, Gers Tusha, Björn Schmidt, Julian J. Holstein, Lars V. Schäfer, Guido H. Clever

**Affiliations:** † Department of Chemistry and Chemical Biology, TU Dortmund University, Otto Hahn Str. 6, 44227 Dortmund, Germany; ‡ Center for Theoretical Chemistry, Ruhr University Bochum, Universitätsstr. 150, 44801 Bochum, Germany

## Abstract

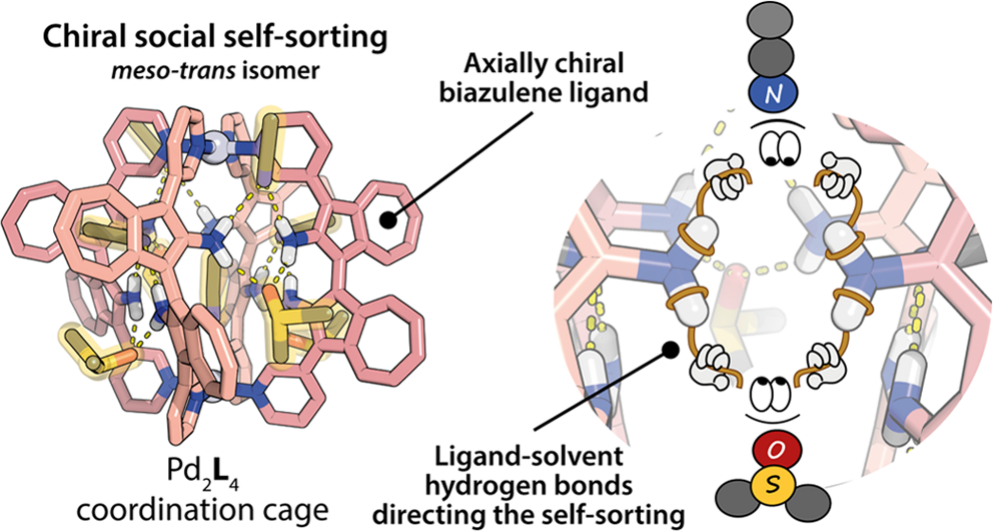

A family of Pd_2_**L**_4_ cages prepared from ligands based on an axially chiral diamino-[1,1′-biazulene] motif (serving as a unique azulene-based surrogate of the ubiquitous BINOL moiety) is reported. We show that preparing a cage starting from the racemate of a shorter bis-monodentate ligand derivative, equipped with pyridine donor groups, leads to integrative (“social”) chiral self-sorting, exclusively yielding the *meso-trans* product, but only in a selection of solvents. This phenomenon is driven by individual solvent molecules acting as hydrogen bonding tethers between the amino groups of neighboring ligands, thereby locking the final coordination cage in a single isomeric form. The experimental (solvent-dependent NMR, single-crystal X-ray diffraction) observations of this cooperative interaction could be explained by computational analyses only when explicit solvation was considered. Furthermore, we prepared a larger chiral ligand with isoquinoline donors, which, unlike the first one, does not undergo social self-sorting from its racemic mixture, further highlighting the importance of solvents bridging short distances between the amino groups. Homochiral cages formed from this larger ligand, however, furnish a cavity that can bind anionic and neutral metal complexes such as [Pt(CN)_6_]^2–^ and Cr(CO)_6_ and discriminate between the two enantiomers of chiral guest camphor sulfonate.

## Introduction

Soluble nanoscopic coordination cages promise to find value-creating application by serving as tailor-made mimics of natural enzymes.^1−4^ Regio- and enantioselective catalysis, as seen in the inner compartments of folded biopolymers, should be achievable in low-symmetry binding pockets of such artificial hosts. To create metallosupramolecular cages with cavities of defined, low-symmetry shape and functional group decoration, however, advanced synthetic strategies are required, including nonstatistical heteroleptic multicomponent assembly^5,6^ and the introduction of chirality. The latter can be achieved through several design approaches such as the use of chiral chelating caps on the metal centers,^7−9^ or vertex-directed chiral pendant groups on the coordination donors,^10−12^ the use of a chiral guest molecule imparting its configuration onto the whole host–guest complex,^13−16^ or the use of enantiomerically pure ligand backbones.^17−24^

To obtain enantiomerically pure ligands, synthesis can start from commercially available chiral building blocks such as BINOL derivatives,^25^ or racemates can be resolved by chiral chromatography. The self-assembly of a coordination cage starting from the racemate of a chiral ligand may result in a mixture of diastereomers if no driving force selects for a specific nonstatistical outcome, but chiral self-sorting may also be observed. In that case, for a Pd_2_**L**_4_ lantern-shaped cage, two separate outcomes are possible: either the ligands narcissistically self-sort to form pairs of homochiral cages, or the two enantiomers “socially” self-sort to form an achiral *meso* compound. In that regard, the use of the racemate of a ligand for the preparation of coordination cages can be regarded as a specific case of multicomponent assembly.^26^ An example of narcissistic self-sorting in Pd-based cages was observed first by Lützen and co-workers, showing that the racemate of a BINOL-based ligand narcissistically self-sorts to form a 1:1 mixture of both Pd_2_**L**_4_ cage enantiomers.^27^ On the other hand, chiral social self-sorting was observed by Claessens and Torrès back in 2002, in a Pd_3_**L**_2_ species formed from racemic ligands with a subphthalocyanine backbone.^28^ Later, we showed a similar type of self-sorting in a Pd_2_**L**_4_ cage system with [6]helicene-based ligands.^29^ There, the racemic ligand socially self-sorts upon coordination to palladium(II) to form the *meso*-*cis* Pd_2_**L**_4_ product.

Similar cases of chiral self-sorting (social and narcissistic) were also reported recently by Natarajan and co-workers who could switch between them by a change of counteranion or solvent,^30−32^ as well by the group of Su, who was able to access different isomers of a cage by a delicate interplay between the supramolecular assemblies and counteranions, solvents, and chiral guest molecules.^33^ The group of Feringa reported narcissistic self-sorting in photoresponsive cages based on molecular motors.^34^ Despite learning from those examples, it is still difficult to predict the assembly outcome of a racemic (vs enantiomerically pure) ligand solely based on its structure and mechanistic details often stay elusive.^35,36^ However, gaining deeper insight into the rules determining chiral self-sorting is important to allow for a more rational approach toward the design of complex assemblies involving chiral ligands.

**Figure 1:**
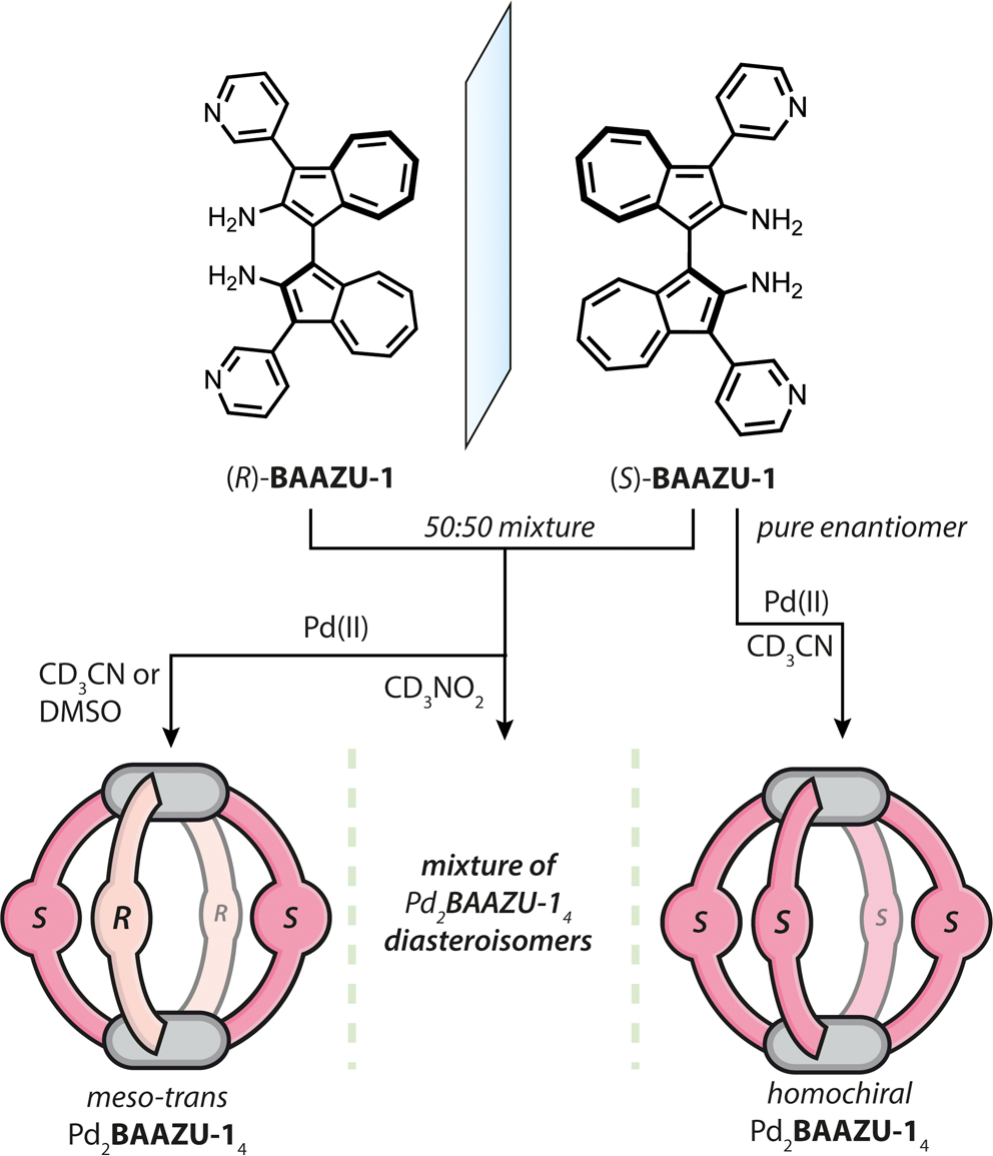
Upon coordination with Pd(II), the racemate of ligand **BAAZU-1** undergoes chiral self-sorting to exclusively form the *meso-trans* cage Pd_2_**L**_4_ when using H-bond acceptor solvents MeCN or DMSO. When poor H-bond acceptor MeNO_2_ is used instead, the system does not self-sort. The homochiral Pd_2_**L**_4_ cage can be obtained from the enantiopure ligand.

Here, we introduce a new axially chiral ligand decorated with hydrogen bond donating amino groups capable of chiral self-sorting within Pd_2_**L**_4_ cages as a result of its specific interaction with H-bond accepting solvent molecules. Based on experimental and computational evidence, we show how solvent-bridging of the amino groups of neighboring ligands drives the system toward a single *RSRS* (“*meso-trans*”) cage diastereoisomer (Figure 1). The ligands used in this study are based on an [1,1′-biazulene]-2,2′-diamine (“**BAAZU**”, for Bis-Amino-AZUlene) backbone, a colored isomer of the corresponding, widely employed binaphthyl units (such as in BINOL, BINAP or BINAM). While a few of such axially chiral biazulenes had already been synthesized,^37,38^ they have remained of little importance outside of the study of their preparation. Concerning the implementation of biazulenes in metallosupramolecular assemblies, we are only aware of a single example reported by Mazaki et al.^39^ Owing to their interesting electronic properties and geometry, we decided to include biazulene building blocks into the preparation of chiral self-assemblies, following our previous work on nonchiral coordination cages based on single-azulene ligands.^40^

## Results and Discussion

**Scheme 1:**
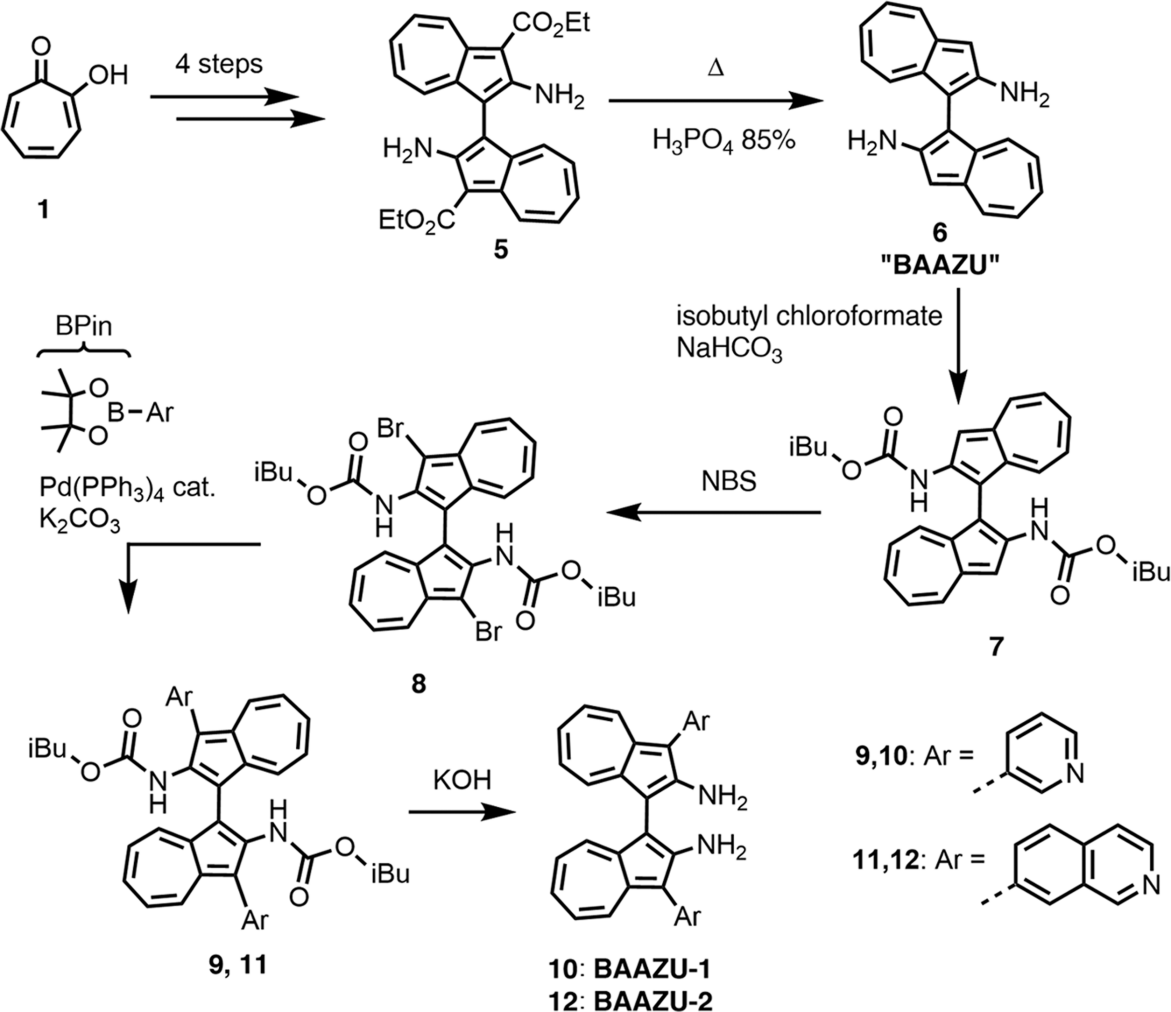
Synthesis Route for the Preparation of Ligands **BAAZU-1** and **BAAZU-2**. Compounds 1 to 5 have already been reported. Full reaction conditions are provided in the SI

Compound **6** was synthesized in five steps from commercially available tropolone (**1**) based on reported synthetic routes toward intermediate **5** (Scheme 1).^37,41−43^ Compound **5** (diethyl 2,2′-diamino-[1,1′-biazulene]-3,3′-dicarboxylate) was decarboxylated in hot H_3_PO_4_ 85% to yield **6** (2,2′-diamino-[1,1′-biazulene], **“BAAZU”**) as the chiral backbone of the ligand. Its structure was confirmed by NMR, mass spectrometry and X-ray crystallography (Subsections 1.2. and 7.1. of the Supporting Information (SI)).

Next, a protection of the amino groups was performed using isobutyl chloroformate to yield intermediate **7**. On a side note, a protection of the amino groups with Boc_2_O was attempted first but failed due to the unwanted formation of a urea bridge between the two amino groups. On the next step, **7** was brominated using *N*-bromo succinimide to yield **8**. A Suzuki coupling was then performed with 3-pyridyl-BPin to yield the protected ligand **9**. On the final step, it was deprotected by heating in a solution of ethylene glycol and KOH to yield the final racemic ligand **BAAZU-1** as a brown powder. Its structure was confirmed by NMR, mass spectrometry and X-ray crystallography (Subsections 1.3. and 7.2. of the SI).

**Figure 2:**
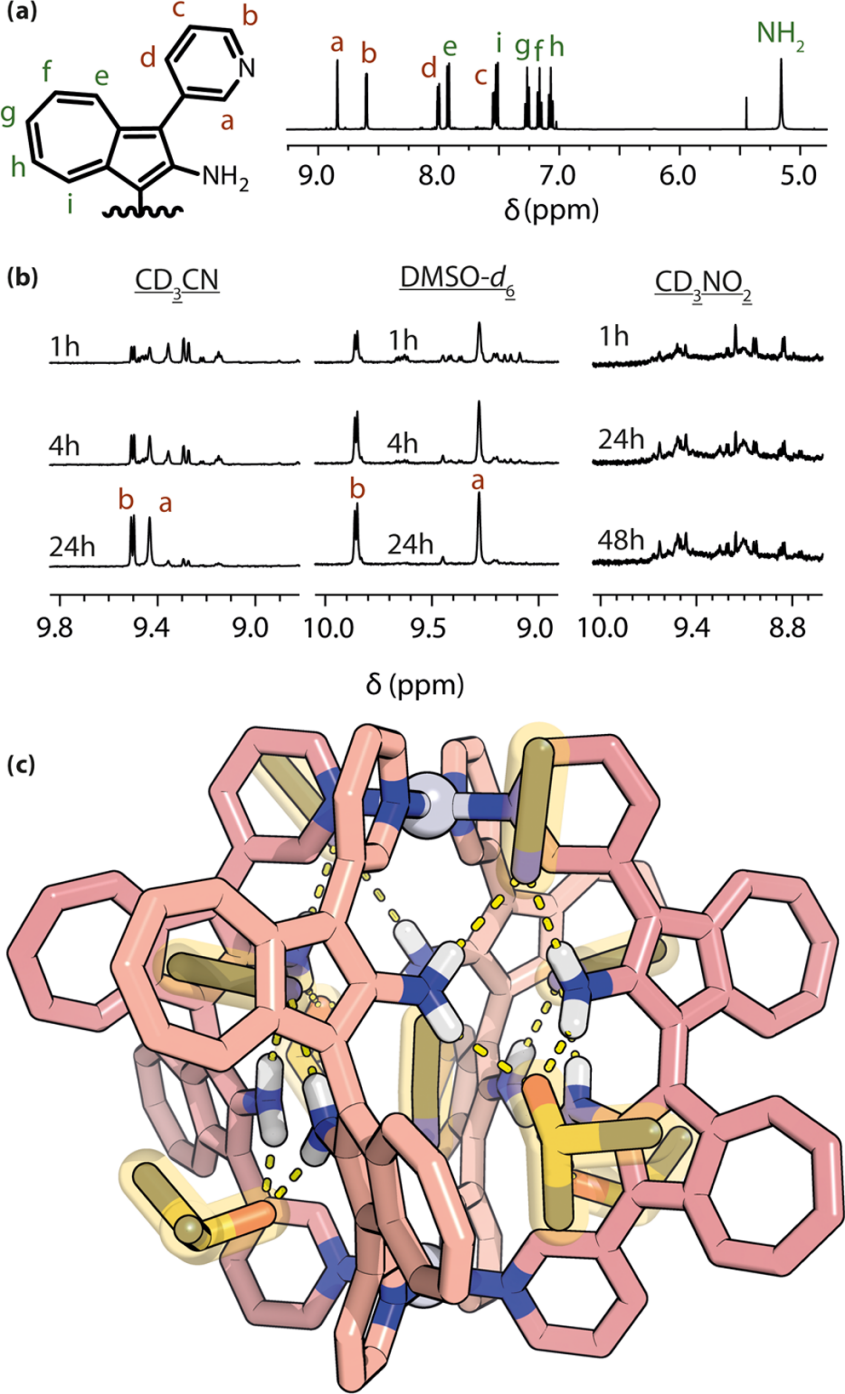
^1^H NMR (600 MHz, 298 K) of (a) **BAAZU-1** in CD_3_CN, and (b) [Pd_2_**BAAZU-1**^**enant**^_4_](BF_4_)_4_ in CD_3_CN (left), DMSO-*d*_6_ (center) and CD_3_NO_2_ (right) over time (partial spectra). (c) X-ray structure of *meso-trans* Pd_2_**BAAZU-1**^**rac**^_4_ with H-bonded CD_3_CN and DMSO solvent molecules (highlighted in orange).

Cage formation from racemic **BAAZU-1** and Pd(II) cations was studied in acetonitrile, DMSO and nitromethane. In acetonitrile, after heating for 1 h at 70 °C, ^1^H NMR analysis revealed a convoluted spectrum showing a multitude of signals. ESI-MS, however, revealed only signals assignable to species Pd_2_**BAAZU-1**_4_ (Figure S30), leading us to conclude that the multiple signals observed by NMR belong to a mixture of several possible cage diastereomers (achiral meso-cages *RRSS* and *RSRS*, and chiral *RRRR* and *RRRS* and their enantiomers; Figure 2b).

After heating for 24 h at 70 °C, however, the NMR spectrum cleared up, showing only one major species (with an equal number of signals as ligand **BAAZU-1**) and some traces of other products. ESI-MS confirmed again that the present species was Pd_2_**BAAZU-1**_4_ (Figure S31). Only two structures, featuring a higher symmetry, out of the four possible isomers can give rise to such a pattern: the homochiral cages *RRRR* and *SSSS* with *D*_4_ symmetry, and the *meso*-*trans* cage *RSRS* with *D*_2d_ symmetry (for a detailed analysis see Figure S33). The same pattern was observed in DMSO, the main species being already present in high abundance after heating at 70 °C for only 1 h. The transformation was complete after 5 h. In nitromethane, however, only a mixture of cages was observed even after heating for 3 days at 70 °C (Figure 2b).

**Figure 3:**
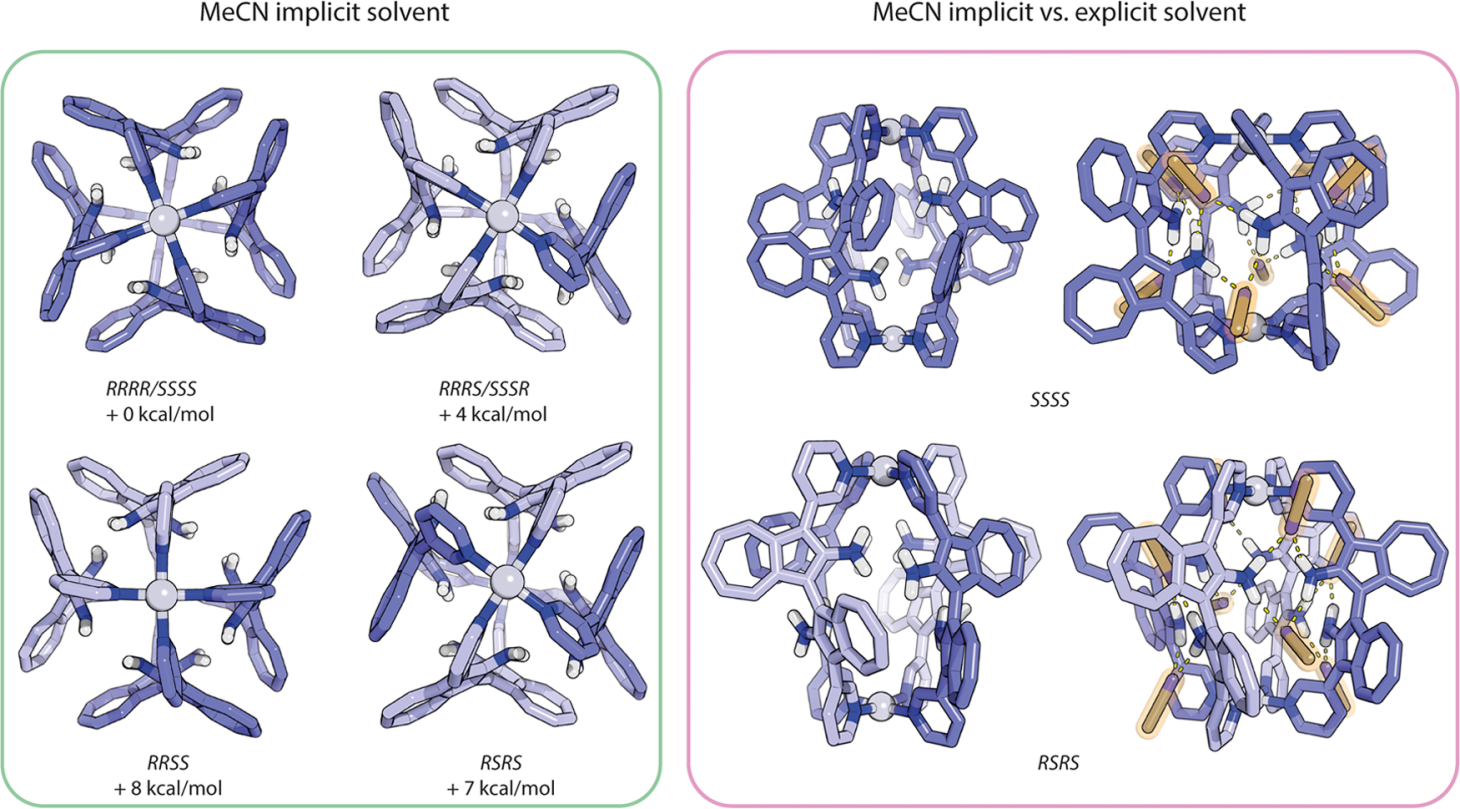
DFT-optimized structures (implicit solvent: MeCN) of the four different possible isomers of Pd_2_**BAAZU-1**^**rac**^_4_ with their corresponding calculated relative energies (left). Comparison of the homochiral and *meso-trans* cage structures obtained with implicit and explicit solvation models (right).

DFT computations were performed to help us determine which of the two possible isomers of the cage was forming. Therefore, the geometries of all four isomers were optimized at the ωB97X-D3/def2-SVP level of theory in implicit solvent (CPCM).^44^ Both DMSO and acetonitrile implicit solvent models yielded the same structures after optimization. Single point energies were computed at the DSD-PBEP86 D3BJ^45,46^/def2-TZVP level of theory (with the same implicit solvent) to calculate the energy differences between the diastereomers. These first calculations suggested that the most stable isomer in solution should be the homochiral *RRRR*/*SSSS* cage (Figure 3). Further, this computational prediction would have been in agreement with the experimental observation of a single set of signals in the ^1^H NMR spectrum, as expected for a racemic mixture of the *D*_4_-symmetric homochiral cages.

Subsequently, we managed to obtain crystals suitable for single crystal X-ray diffraction (SCXRD) from the slow diffusion of diisopropyl ether into a solution of the cage in acetonitrile and 10% DMSO. To our surprise, and contrary to the most stable isomer predicted by the first round of computations, the structure obtained here was the *meso-trans RSRS* cage (Figure 2c and subsection 7.4. of the SI). One striking feature of the structure revealed by SCXRD is the close proximity of the amino groups of neighboring ligands (approximately 3.5 Å between the nitrogens) and their corresponding azulene subunits being almost perfectly aligned in a single plane. Indeed, the structure revealed that each pair of amino groups is bridged by two solvent molecules, one above and one below, with their H-bond acceptor group (−CN or –SO) in close proximity to the hydrogens of the amino groups in a 2.2 to 2.7 Å range, which is typical for H-bonding distances. No π-stacking was observed between the ligand backbones or between cages within the crystal packing.

To confirm that the species in solution is indeed this same *meso-trans* cage, and the obtained isomer in the solid-state structure is not an artifact of the crystallization process, the two enantiomers of the **BAAZU-1** ligand were separated by semipreparative chiral HPLC (Figure S21). The absolute configuration of the enantiomers could be determined by X-ray crystallography (Subsection 7.2. of the SI): first eluting fraction 1 corresponds to the *R*-enantiomer, and thus fraction 2 to the *S*-enantiomer. This assignment was further confirmed by comparing experimental and TD-DFT-computed CD spectra (more details in Sections 6 and 8 of the SI).

**Figure 4:**
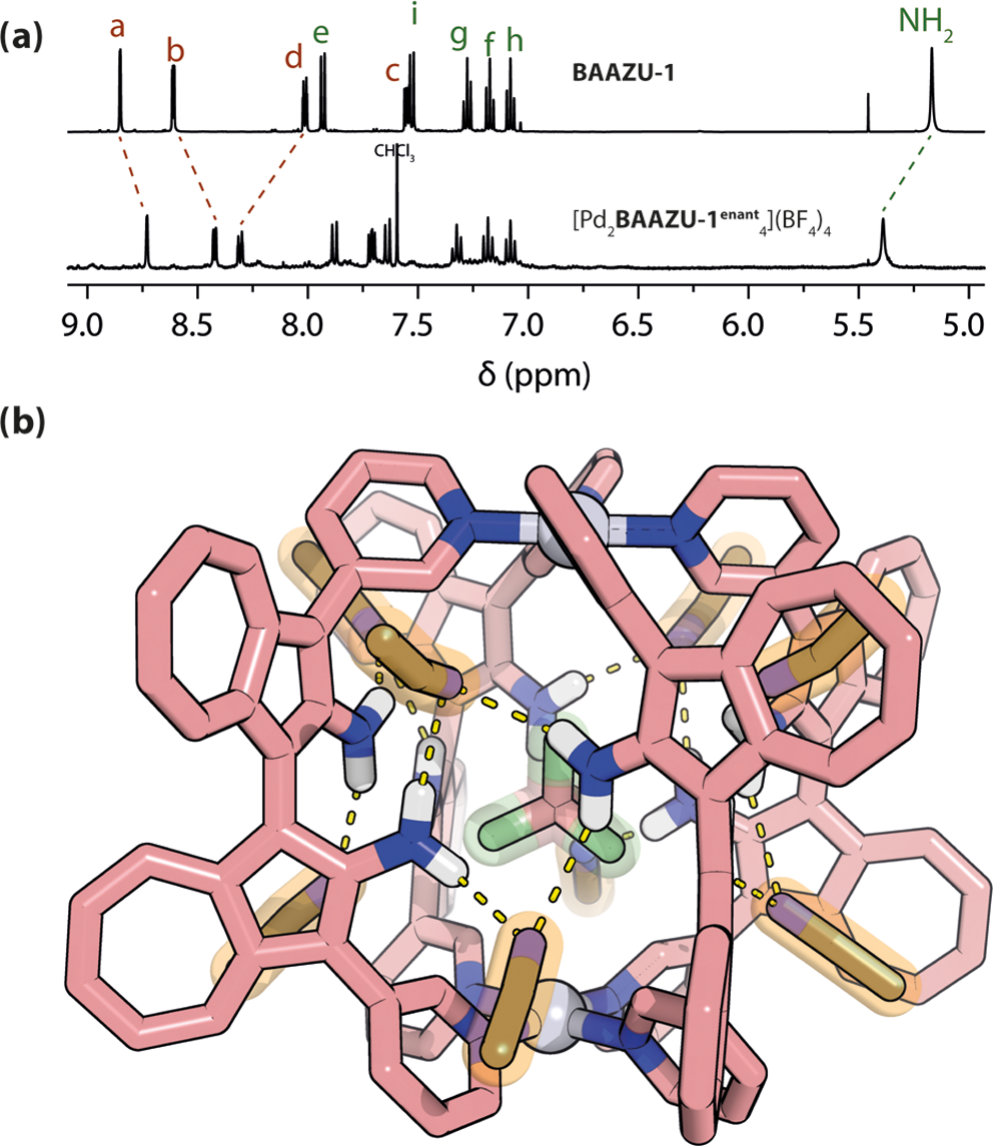
(a) ^1^H NMR (500 MHz, CD_3_CN, 298 K) of **BAAZU-1** (top) and [Pd_2_**BAAZU-1**^**enant**^_4_](BF_4_)_4_ in CD_3_CN (bottom). (b) X-ray structure of Pd_2_(*S*)-**BAAZU-1**_4_ with H-bonded solvent molecules and the central encapsulated BF_4_^–^ anion. Remaining solvent molecules and counteranions were omitted for clarity.

Due to concerns about the racemization of the ligand upon heating, the formation of the cage with the enantiopure ligand was done at room temperature in CD_3_CN. After 12 h of stirring at RT, the ^1^H NMR of the solution was recorded. In acetonitrile, a single species with the same number of NMR signals as the ligand is visible, but with pronouncedly different shifts than the *meso-trans* cage described above (Figure 4a). Furthermore, single crystals of the cage formed from (*S*)-**BAAZU-1** were grown from slow diffusion of diethyl ether into the acetonitrile solution. The structure revealed by SCXRD confirms the formation of the homochiral cage (Figure 4b and subsection 7.5. of the SI). The solid-state structure of the cage bears some resemblance with the previously described one, where eight solvent molecules bridge four pairs of amino groups. Due to the structural differences, though, the distance between the diagonally arranged NH_2_ groups and the bridging solvent molecules was found to be higher on average (2.3 Å for the *meso-trans* and 2.5 Å for the homochiral cage), indicating a lower degree of stabilizing H-bonding interactions in the latter case.

The cavity of the newly formed species contains one BF_4_^–^ counteranion as observed by X-ray crystallography (Figure 4b). On the contrary, the cavity of the *meso-trans* cage contains one MeCN solvent molecule (Figure 2b). The calculated volumes for both cavities are however similar: 60 Å^3^ for the *meso-trans* species, vs 63 Å^3^ for the homochiral cage (Figure S70). The difference in guest preference may thus be explained by the shape of the cavities and Pd–Pd distances (10.6 vs 9.3 Å). Indeed, the *meso-trans* cage has a prolate cavity, capable of better fitting a MeCN molecule, whereas the spherical shape of the cavity of the homochiral cage corresponds better to a tetrafluoroborate anion.

Looking at the positioning of these H-bonding solvent molecules, we therefore hypothesized that their presence is necessary for the self-sorting leading to the *meso* cage. Indeed, as already described above, dissolving the racemic ligand in nitromethane does not show any self-sorting even after extensive heating, as nitromethane is a poor H-bond acceptor. To support this idea, we titrated the nitromethane cage solution with DMSO. After the addition of 25 μL of DMSO to the 0.7 mM cage solution (approximately 1000 equiv, 5% vol.), only one major species was visible, as well as some minor signals (Figure S32). Hence, this experiment supports the necessity of strong H-bond acceptors to trigger the self-sorting into the single *meso-trans* cage.

Hydrogen bonds have already been shown to be major contributors to the self-sorting of coordination assemblies, for example in the work of Natarajan and co-workers,^47^ where they showed that interligand H-bonds were driving the formation of a single isomer of a Pd_2_**L**_4_ cage, self-assembled from asymmetric building blocks based on a steroid backbone. The group of Crowley used ligands functionalized with amino groups on the pyridyl donors to prepare a series of reduced-symmetry Pd_2_**L**_4_ cages, where the interactions between the amino groups and polarized hydrogens on neighboring donors were determined to be the driving force behind the nonstatistical assembly.^48,49^ The group of Lewis recently demonstrated that a Pd_2_**L**_4_ cage assembled from asymmetric ligands can switch between the *trans* and the *cis* isomers upon changing the solvent from CD_3_CN to DMSO and justified this transformation by a strong stabilizing interaction of DMSO with polarized C–H bonds around the coordination spheres.^50^ Nitschke and co-workers similarly revealed the role of interligand H-bonds in self-assembled structures, whose structures could be modulated by the solvent (MeCN or MeOH)^51^ or sterics.^52^ However, unlike the present case, the direct involvement of discrete solvent molecules was not observed in those studies. The central role of solvent-mediated H-bonding revealed by the crystal structures pointed to limitations of the initial DFT calculations, in which the solvent was taken into account only with an implicit model.

For this reason, on the basis of the experimental X-ray structures, different cage structures with increasing complexity concerning their solvent environment were modeled (Subsection 6.1. of the SI). On the first level, eight discrete solvent molecules (acetonitrile or DMSO) were included with the aim of maximizing the H-bonding pattern (Figure S63). Including explicit solvent molecules indeed allowed to partially overcome the discrepancies of the previous calculation results to the experiments, where the *RSRS* isomer was computed to be 7 kcal/mol higher in energy than the *RRRR* (or *SSSS*) isomer. In the new ranking, the *RRRR* and *RSRS* cages became now very close in energy (Table S2).

Adding a second layer of complexity, one BF_4_^–^ counterion and an additional acetonitrile molecule were included to further increase the similarity of the computed models to the crystal structures (where one BF_4_^–^ was found inside the *SSSS* and one acetonitrile inside the *RSRS* isomer; in the respective other case the anion/solvent was placed outside close to one of the outer Pd(II) cations). The energetic ranking of the isomers was computed again with the same protocol described above, leading to a further improved agreement with the experiments: now the *RSRS* cage is the most stable isomer, with a difference in Gibbs free energy of 7 kcal/mol compared to *RRRR* (or *SSSS*; Table S3). These results demonstrate the importance of explicitly considering solvent-mediated interactions to accurately describe the systems and capture the energetic ranking of the stereoisomers, which can become a challenge in the absence of crystallographic data.

Next, we investigated the stability of the cage toward racemization. First, the racemization of the ligand was monitored in acetonitrile by measuring the decay of the CD signals over time at several different temperatures. Eyring analysis yielded a racemization Gibbs free energy of activation of Δ*G*^‡^ = 110.3 kJ/mol (26.4 kcal/mol; Figure S48). Interestingly, the racemization rate was slowed down when measured in DMSO (Figure S50). We hypothesize that this change in rate may be caused by stronger H-bond interactions between the amino groups of the ligand and the solvent. However, more investigations are needed to accurately describe the underlying process.

**Figure 5:**
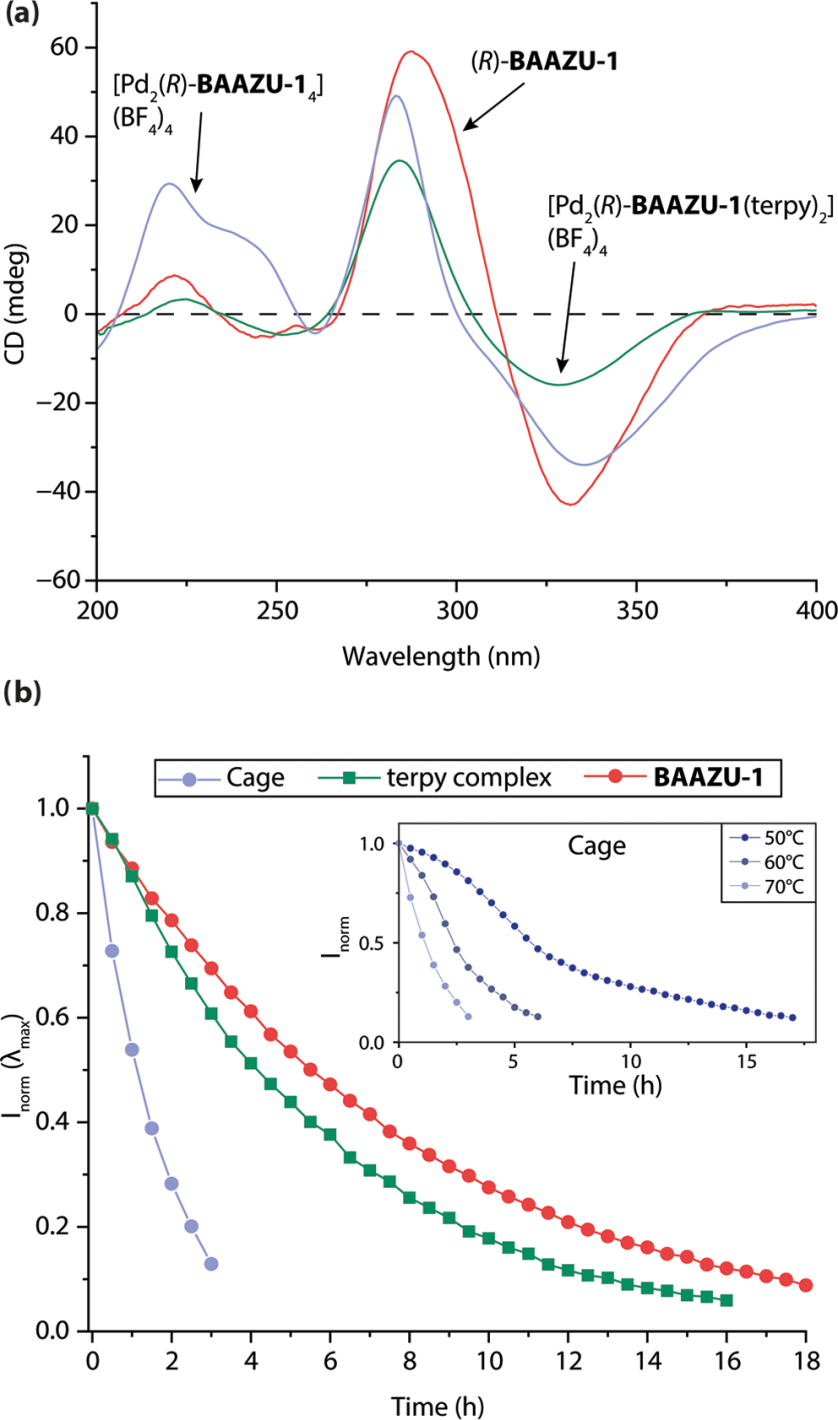
(a) CD spectra of (*R*)-**BAAZU-1**, complex [(Pd-terpy)_2_(*R*)**-BAAZU-1**](BF_4_)_4_ and cage [Pd_2_(*R*)**-BAAZU-1**_4_](BF_4_)_4_ in MeCN at 70 °C (0.125 mM in ligand, 2 mm cuvette, expect for the terpy complex: 1 mm cuvette). (b) Decay over time of the CD signal of **BAAZU-1** and of its two coordination species in MeCN at 70 °C measured at their λ_max_. The inset shows the sigmoidal decay of the signal of the cage at 50, 60, and 70 °C.

Astonishingly, the cage racemized much faster than the ligand in acetonitrile. Measurements by CD spectroscopy showed that a decrease of the signal intensity by 50% only required slightly more than 1 h (Figure 5b). Moreover, the decay of the CD signal does not follow a first order rate law; the shape of the curve looks sigmoidal at lower temperatures (Figures 5b and S46), leading us to infer a cooperative pathway to racemization of the cage. Indeed, without considering the exchange of ligands between cages, the epimerization of one ligand on the homochiral starting cage energetically follows an uphill pathway (according to the computed energies of the four isomers). The next epimerization, however, leads to the *meso-trans* cage (or in a lesser extent to *meso-cis*), and is energetically downhill. Assuming that the epimerization kinetics correlate with the thermodynamic drive for the consecutive steps, the sigmoidal shape of the observed reaction progress seems reasonable. But the mechanism might be more complicated, considering the required breaking and reforming of ligand-solvent H-bonded bridges during the cage racemization (as opposed to the racemization of the free ligand) and the expulsion of the BF_4_^–^ guest anion from the cavity of the homochiral cage. Also, differences in the electronic situation of the free ligand to the metal-coordinated ones may affect the rotational barrier around the central C–C bond of the biazulene. NMR studies also showed that the product of the racemization of the homochiral cage was the *meso-trans* isomer. Moreover, the racemization rate was faster in DMSO than in MeCN (Figures S36 and S37), unlike what was observed in the case of the free ligands (Figure S50).

In order to evaluate if the electron-withdrawing nature of the coordinated Pd(II) cations influence the racemization rate of the ligand, we monitored the racemization of a [(Pd-terpy)_2_**BAAZU-1**^**enant**^](BF_4_)_4_ complex by CD spectroscopy, prepared by mixing the homochiral ligand and 2 equiv of [Pd(terpy)(CH_3_CN)](BF_4_)_2_. In this model compound, the electron-withdrawing Pd(II) centers of the cage are simulated but the ligand is left free of close interactions with other ligands. While the racemization rate of this complex was found to be slightly faster than the one of the free ligand at the same temperature (Figure 5b), it is by far not as fast as the one observed for the cage, meaning that the Pd(II) coordination cannot alone explain the dramatic racemization rate increase of the cage, let alone the sigmoidal shape of its decay curve.

The racemization was then also investigated from a computational point of view. Models with increasing complexity (from the BAAZU moiety to the (Pd-terpy)_2_-complex) were considered and the isomerization energy barriers were computed at the DLPNO–CCSD(T)/def2-TZVPP^53^ level (Subsection 6.2. of the SI). The energy barriers obtained are in good agreement with the experimentally determined values and validate our models: the racemization Gibbs free energy barrier for both **BAAZU-1** and [(Pd-terpy)_2_**BAAZU-1**^**enant**^] was calculated to be 28 kcal/mol, close to the 26.9 kcal/mol measured for the ligand. The computations thus confirm that the rate acceleration of the cage epimerization does not have an electronic origin coming from the coordination to the Pd(II) cations.

To further confirm that chiral self-sorting leading to the *meso-trans* Pd_2_**BAAZU-1**_4_ cage is indeed dependent on bridging ligand-solvent interactions, a second ligand, **BAAZU-2**, was synthesized, bearing 7-isoquinoline donor groups (Scheme 1). Due to the larger donors, we hypothesized that the increased distance between the amino groups of neighboring ligands in the final assembly would reduce or even cancel the solvent-dependent self-sorting through H-bond bridges. And indeed, when using the racemic **BAAZU-2** ligand to form the cage, the spectra of the resulting solutions showed no self-sorting whatsoever in all solvents tested (acetonitrile, DMSO, and nitromethane; Figure S38). This result further denotes the importance of H-bond bridges between neighboring –NH_2_ groups and solvent molecules in the first structure.

The two enantiomers of **BAAZU-2** could also be separated by chiral HPLC (Figure S22), and the corresponding enantiopure cages were prepared in CD_3_CN by the addition of Pd(II) to the enantiopure ligand solution and heating at 70 °C for 5 min. The formation of the Pd_2_**L**_4_ cage was confirmed by ^1^H NMR and ESI-MS (Figures S40–S43). Interestingly, the Cotton effect of the first fraction of **BAAZU-2** is positive, unlike **BAAZU-1**, where it is negative (Figure S46). The first fraction of **BAAZU-2** was assigned to the (*S*)-enantiomer, while fraction 2 was assigned to the (*R*)-**BAAZU-2**. This assignment was performed through TD-DFT only (Figure S68), as the data from X-ray crystallography was not of good enough quality (Subsection 7.6. of the SI).

**Figure 6:**
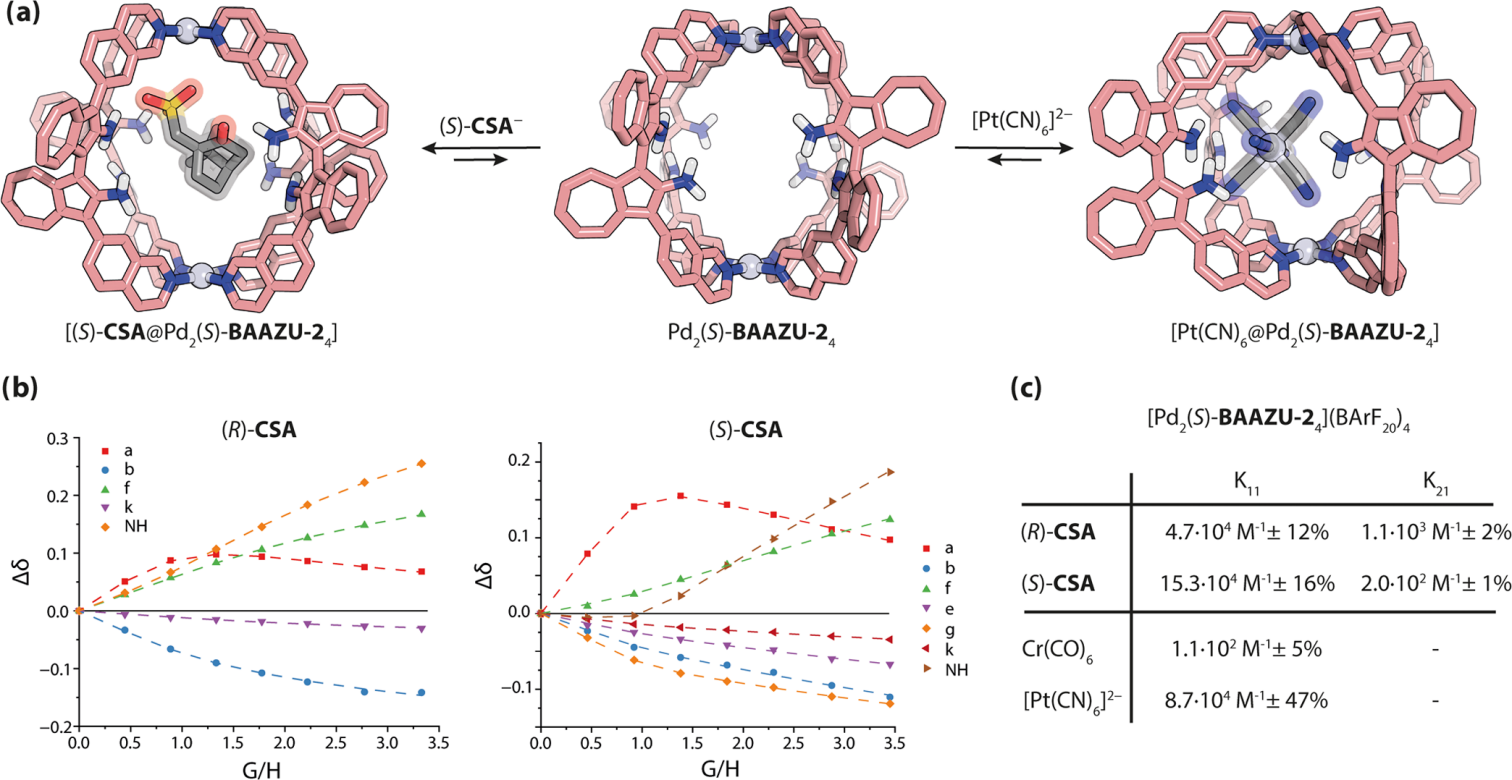
(a) Encapsulation of guests into the Pd_2_(*S*)-**BAAZU-2**_4_ cage (DFT structures, ωB97X-D or ωB97X-D3/def2-SVP). (b) ^1^H NMR titration curves of (*R*)- and (*S*)-**CSA** into cage [Pd_2_(*S*)-**BAAZU-2**_4_](BArF_20_)_4_ (scatter) with the BindFit fitting (dashed lines). (c) Binding constants for the four tested guests.

Prompted by the larger cavity (414 Å^3^, Figure S70) compared to the **BAAZU-1** cages (60–63 Å^3^), and the presence of eight -NH_2_ units on the rim, we tested the binding of guests with H-bond acceptor character inside the Pd_2_**BAAZU-2**^**enant**^_4_ cage (Figure 6a). In a previous report,^54^ we were able to bind octahedral metal complexes such as neutral chromium(0) hexacarbonyl or dianionic hexacyanoplatinate(IV) in a Pd_2_**L**_4_ cage with four inward pointing hydrogens from succinimide groups. Here, the binding of these metal complexes was studied by ^1^H NMR, UV–vis absorption and CD spectroscopy in acetonitrile, using BArF_20_^–^ (tetrakis(pentafluorophenyl)borate) as a large, noncompetitive counteranion.^55^ Neutral Cr(CO)_6_ was observed to bind weakly inside the cage through a shift of the inward pointing protons of **BAAZU-2**, while dianionic [Pt(CN)_6_]^2–^ is binding strongly in rapid exchange on the NMR time scale (Figures S56 and S57) in a 1:1 ratio (Figure 6c). Unlike the previous report,^54^ the C≡N stretching band of the guest in the host–guest complex could not be observed in the IR spectrum (Figure S60).

DFT models (ωB97X-D/def2-SVP) of the cage and of the host–guest complexes were prepared (Figure S69). The distance between the amino groups in the free cage were calculated to be 7.0 Å (N to N), a notable increase from the average 3.6 Å in the Pd_2_**BAAZU-1**^**rac**^_4_ cage. These longer distances do explain the inability for the solvent molecules to form H-bond bridges between the ligands, and by consequence the lack of self-sorting observed in Pd_2_**BAAZU-2**^**rac**^_4_. In the case of the octahedral metal complexes, the guests arrange on one of the sides of the cavity of the cage and four of their hydrogen-bond accepting atoms make contacts with the ligands’ amino groups. As no splitting was observed in the NMR spectra of the host–guest complexes (Figures S55 and S57), it is reasonable to assume that the gas-phase DFT models only represent a static snapshot of a highly dynamic host–guest complex at ambient temperatures in solution, where the guest tumbles around inside the cage and is not stalled in a single fixed position or orientation. Previous reports by Amouri and co-workers and by Juber and Schäfer support this hypothesis.^56,57^

Chiral guest discrimination was also tested with the cage by titration of (*R*)- and (*S*)-camphor sulfonate (**CSA**) into the homochiral cage. Inflection points in the ^1^H NMR titration graphs for both experiments suggested 1:2 (host:guest) binding (Figure 6b). This model was further confirmed by ESI-MS (Figure S62). Both guest enantiomers were shown to strongly bind in the (*S*)-cage in the first association event, with a 3-fold preference for (*S*)-**CSA** over (*R*)-**CSA**, showing a significant power for chiral guest discrimination (Figure 6c). The binding constants of the second event being both much weaker than the first, together with the size of the guest compared to the cavity (as seen by DFT calculations) suggest a loose binding of the second guest, probably to the cage’s apertures.

## Conclusions

We prepared a series of new Pd_2_**L**_4_ assemblies based on a chiral 2,2′-diamino-[1,1′-biazulene] backbone. The racemate of the first **BAAZU-1** ligand self-assembles into a *meso-trans* cage upon addition of Pd(II) through social chiral self-sorting. This phenomenon is strongly dependent on the solvent used, as it is required to form hydrogen bond bridges between the amino groups of neighboring ligands. Experimental results could be only explained by computational models when individual solvent molecules were explicitly considered. Our findings showcase that solvent effects, serving as key determinants for the outcome of metal-mediated assembly processes, may not be negligently treated by continuum solvation models without risking to overlook specific stabilizing interactions between individual solvent molecules and closely arranged features of the nanoscopic architecture.

A second cage, based on the larger ligand **BAAZU-2**, was shown to not undergo self-sorting, as the amino groups are too far apart from each other to form hydrogen bond bridges with the solvent molecules. The corresponding homochiral cage, however, binds neutral and negatively charged metal complexes, partly through hydrogen-bonding between the ligands and the guests. The chiral nature of Pd_2_**BAAZU-2**^**enant**^_4_ and its ability to bind transition metal complexes bears potential to use it for regio- or enantioselective (photo)catalytic transformations within its nanoconfined cavity, lined by eight azulene chromophores. It can further effectively discriminate between the two enantiomers of a camphor sulfonate guest, making it an attractive starting point for the development of colorimetric receptors for chiral analytes.

## Supporting Information Available

The experimental section (synthetic procedures, NMR, MS, CD spectroscopic data) (PDF)
DFT calculation data (ZIP)
