# Supplementary material for: Solvent-Directed Social Chiral Self-Sorting in Pd2L4 Coordination Cages
Source: J Am Chem Soc. 2024 Nov 17;146(47):32748–56. doi: 10.1021/jacs.4c12525 (PMC11626499; doi:10.1021/jacs.4c12525)
Supplement: Supplementary file 1 — ja4c12525_si_001.pdf [file ja4c12525_si_001.pdf]

Solvent-directed Social Chiral Self-Sorting in Pd_2L_4 Coordination Cages

Alexandre Walther[1], Gers Tusha[2], Björn Schmidt[1], Julian J. Holstein[1], Lars V. Schäfer[2], and Guido H. Clever[1*]

[1] Dept. of Chemistry and Chemical Biology, TU Dortmund University, Otto Hahn Str. 6, 44227 Dortmund, Germany.

[2] Center for Theoretical Chemistry, Ruhr University Bochum, Universitätsstr. 150, 44801 Bochum, Germany.

1. Synthetic procedures 2
  1.1. Synthesis of [1,1'-biazulene]-2,2'-diamine (“BAAZU“) 2
  1.2. Synthesis of 3,3'-di(pyridin-3-yl)-[1,1'-biazulene]-2,2'-diamine (“BAAZU-1“) 8
  1.3. Synthesis of 3,3'-di(isoquinolin-7-yl)-[1,1'-biazulene]-2,2'-diamine (“BAAZU-2“) 13
  1.4. $[Pd(CD_3CN)_4](BArF_{20})_2$ 16
2. Chiral separation of the ligands by HPLC 17
3. Preparation of the cages 19
  3.1. Pd_2BAAZU-1^{rac}_4 from the racemic ligand 19
    3.1.1. Evolution of NMR signals sets after addition of Pd(II) to the racemic ligand over time 21
    3.1.2. Titration of Pd_2**BAAZU-1^{rac}**$_4$ in CD_3NO_2 with DMSO-d_6 23
    3.1.3. Symmetry of the four diastereomers of the cage 24
  3.2. Pd_2BAAZU-1^{enant}_4 from enantiomerically pure ligand 25
  3.3. Pd_2BAAZU-2^{rac}_4 from racemic ligand 29
  3.4. Pd_2BAAZU-2^{enant}_4 from enantiomerically pure ligand 30
4. Circular dichroism spectra 32
  4.1. CD of Intermediate 8 32
  4.2. CD of BAAZU-1 33
  4.3. CD of BAAZU-2 33
  4.4. Racemisation kinetics of BAAZU-1 34
  4.5. Racemisation kinetics of $[Pd_2$BAAZU-1$(terpy)_2](BF_4)_4$ 36
  4.6. Racemisation kinetics of $[Pd_2$BAAZU-$1^{enant}_4](BF_4)_4$ 36
  4.7. Racemisation of $[Pd_2$BAAZU-$2^{enant}_4](BF_4)_4$ 37
5. Host-guest experiments 37
  5.1. $Cr^0(CO)_6$ 37
  5.2. $(NBu_4)_2[Pt^{IV}(CN)_6]$ 38
  5.3. Camphorsulfonate (tetrabutyl ammonium salt) 41
6. Computations 42
  6.1. Computational investigation of the energetic ranking of the isomers 42
  6.2. Computational investigation of the ligand isomerisation 45
  6.3. Calculation of ECD spectra 46
  6.4. BAAZU-2 cage and host-guest complexes 48
7. X-ray crystallography 50
  7.1. BAAZU (6) 55
  7.2. BAAZU-1 Fraction 1 55
  7.3. Diisobutyl (3,3'-dibromo-[1,1'-biazulene]-2,2'-diyl)dicarbamate (8) fractions 1 & 2 56
  7.4. $[Pd_2$BAAZU-$1^{rac}_4](BF_4)_4$ 57
  7.5. $[Pd_2(S)$-BAAZU-$1_4](BF_4)_4$ 58
  7.6. BAAZU-2 Fraction 2 59
8. Assignment of the absolute configuration of BAAZU-derivatives 60
9. References 61

1. Synthetic procedures

1.1. Synthesis of [1,1'-biazulene]-2,2'-diamine ("BAAZU")

General scheme

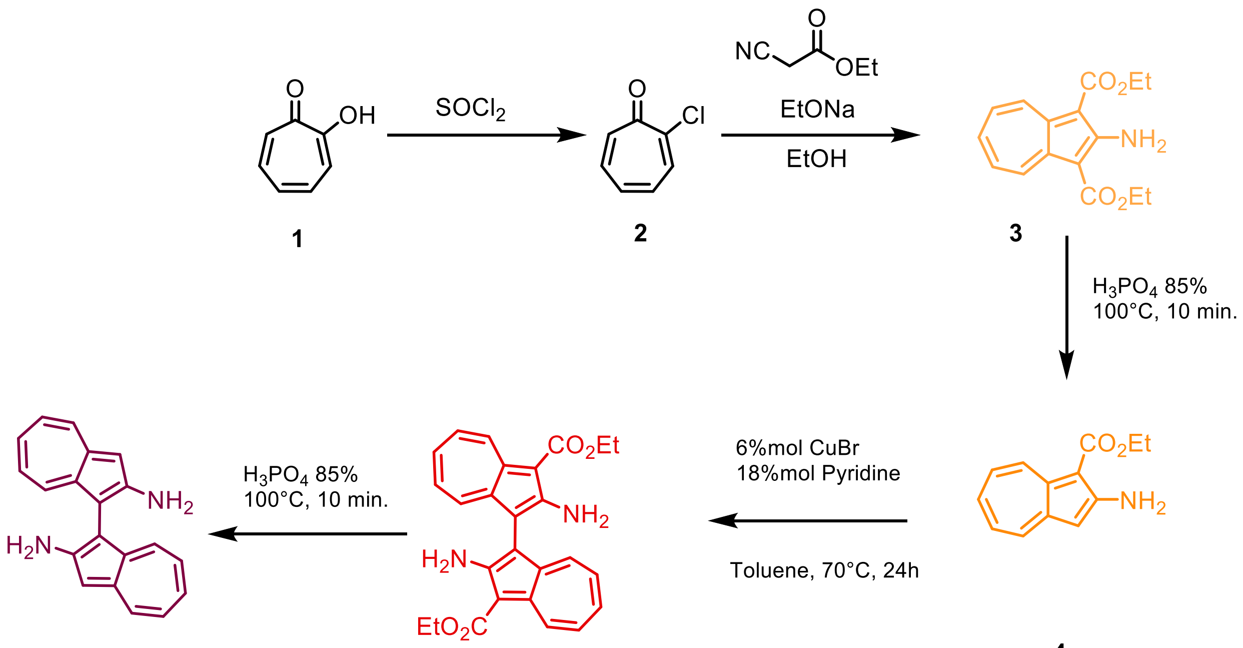

Scheme S1: Synthesis of compounds **2** to **6**.

Compounds **2** to **5** were synthesised according to reported literature procedures.

2-Chlorotropone (**2**):[1]

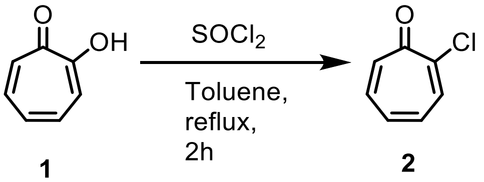

Scheme S2: Synthesis of compound **2**.

Tropolone **1** (1 g, 8.19 mmol) was dissolved in dry toluene (25 ml) and $SOCl_2$ (0.773 ml, 1.27 g, 10.65 mmol) was added dropwise to the stirring solution which was then refluxed for 2 hours under argon. After cooling down, the solvent was removed under low pressure. The product was purified from the resulting crude dark oil by repeated extraction with hot hexane and recrystallisation, to yield 2-chlorotropone **2** (1.08 g, 7.67 mmol) as off-white needles. Yield: 72%

^{1}H-NMR (500 MHz, DMSO-d_6) δ 8.02 (d, J = 9.4 Hz, 1H), 7.44 – 7.37 (m, 1H), 7.30 – 7.24 (m, 1H), 7.17 (d, J = 12.2 Hz, 1H), 7.10 (t, J = 10.1 Hz, 1H).

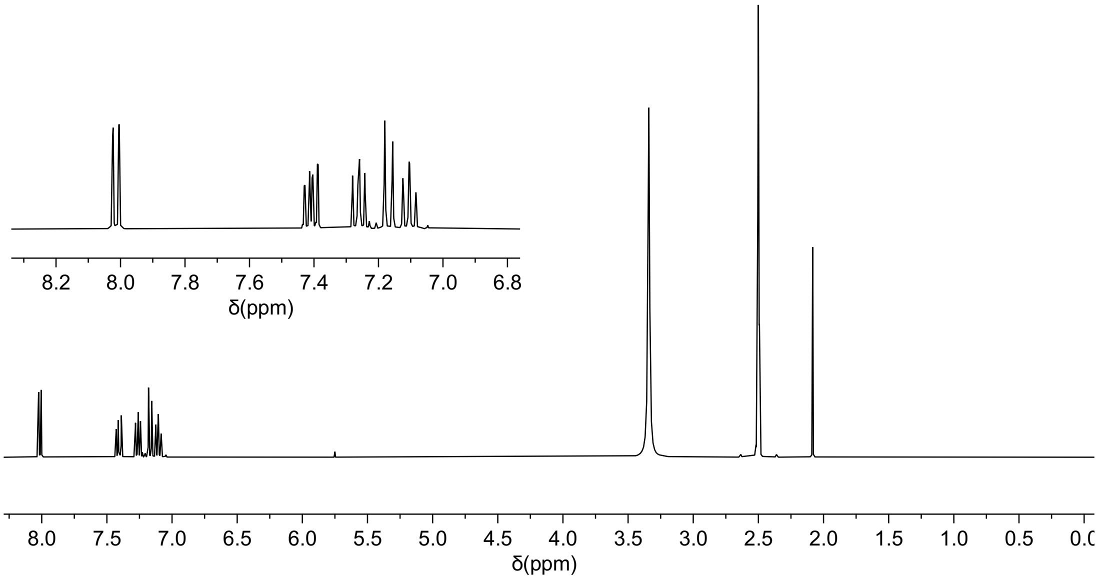

Figure S1: ^{1}H NMR (500 MHz, 298 K, DMSO-d_6) spectrum of 2-chlorotropone (**2**). The aromatic region is presented in the insert.

Diethyl 2-aminoazulene-1,3-dicarboxylate (**3**):[2]

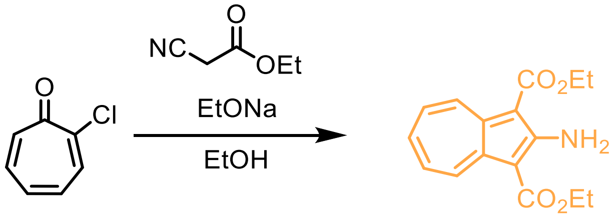

2 **3**

Scheme S3: Synthesis of compound 3.

Metallic sodium (403 mg, 17.5 mmol) was added to 30 ml of absolute ethanol and was left to react. Once done, the solution was then cooled down in an ice bath and ethyl cyanoacetate (3.2 g, 15.78 mmol, 3 ml) was slowly added to it under an argon atmosphere. A solution of 2-chlorotropone **2** (1 g, 7.11 mmol) in ethanol was then added dropwise. The reaction mixture turned immediately yellow. It was left to stir at RT overnight.

Afterwards, the reaction was quenched with water and it was extracted with DCM. The organic layer was dried with $MgSO_4$ and evaporated under low pressure. The crude was recrystalised from aqueous ethanol to yield the title compound **3** as orange crystals (890 mg, 3.13 mmol). Yield: 44%.

^{1}H-NMR (500 MHz, DMSO-d_6) δ 9.09 (d, J = 10.4 Hz, 2H), 7.83 (s, 2H), 7.69 (t, 2H), 7.57 (t, J = 9.6 Hz, 1H), 4.39 (q, J = 7.1 Hz, 4H), 1.39 (t, J = 7.1 Hz, 6H).

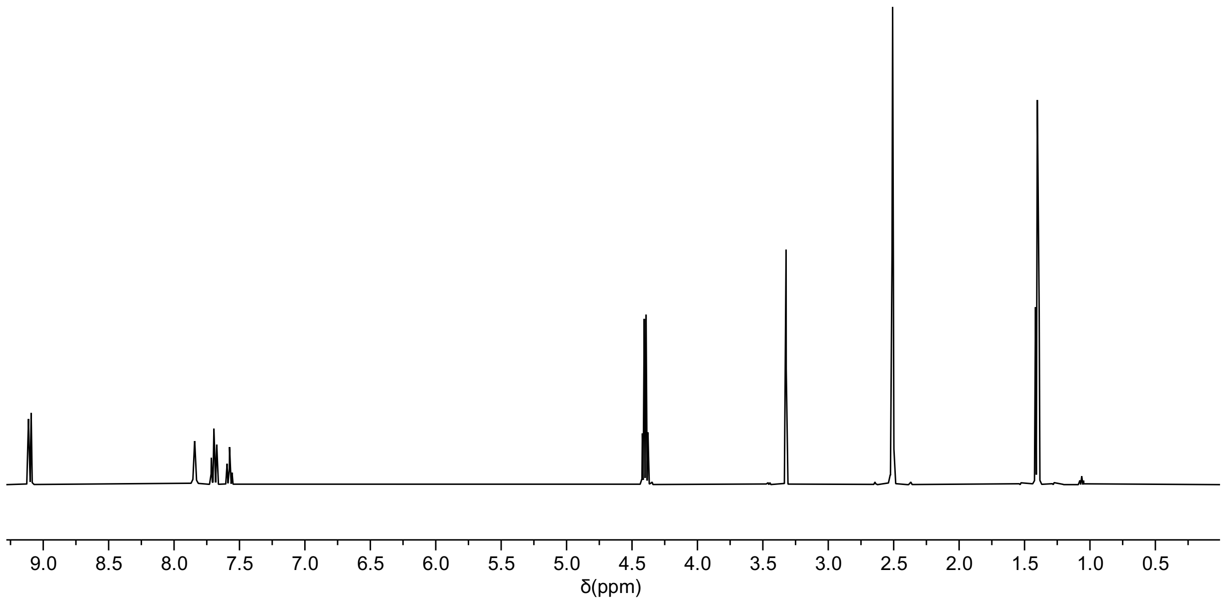

Figure S2: ^{1}H NMR (500 MHz, 298 K, DMSO-d_6) spectrum of **3**.

Ethyl 2-aminoazulene-1-carboxylate (**4**)[3]:

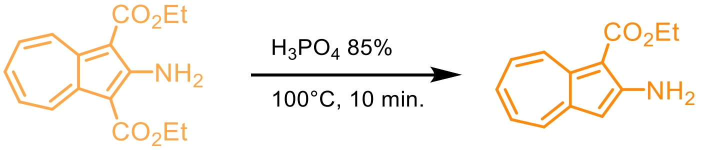

3 **4**

Scheme S4: Synthesis of compound **4**.

Diethyl 2-aminoazulene-1,3-dicarboxylate **3** (1.44 g, 5 mmol) was added to a round-bottom flask containing 10 ml of H_3PO_4 85%. The flask was then added to a pre-heated oil bath at 100°C, and the mixture was stirred 10 minutes. Gas evolution was observed. Afterward, the solution was left to cool down to RT, and was then added to 50 ml of water. The resulting solution was neutralised with NaOH and extracted with EtOAc. The organic layer was dried with $MgSO_4$ and the solvent was removed under low pressure to yield ethyl 2-aminoazulene-1-carboxylate **4** as an orange product (980 mg, 4.55 mmol). Yield: 91%.

^{1}H-NMR (500 MHz, DMSO-d_6) δ 8.65 (d, *J* = 9.6 Hz, 1H), 7.85 – 7.77 (m, 1H), 7.31 – 7.24 (m, 1H), 7.22 – 7.12 (m, 2H), 6.56 (s, 1H), 4.33 (q, *J* = 7.1 Hz, 2H), 1.37 (t, *J* = 7.1 Hz, 3H).

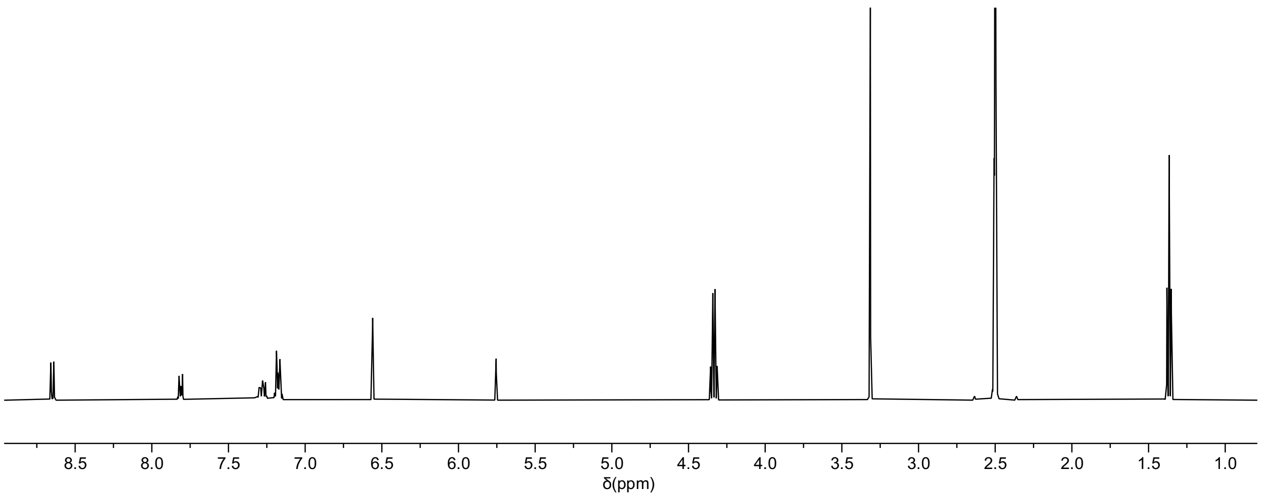

Figure S3: ^{1}H NMR (500 MHz, 298 K, DMSO-d_6) spectrum of **4**.

Diethyl 2,2'-diamino-[1,1'-biazulene]-3,3'-dicarboxylate (**5**):[4]

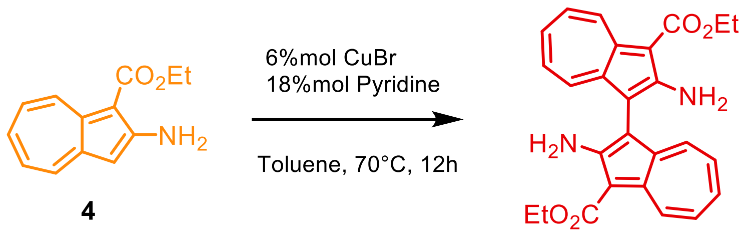

Scheme S5: Synthesis of compound **5**.

To a round bottom-flask charged with 50 ml of toluene were added ethyl 2-aminoazulene-1-carboxylate **4** (535 mg, 2.5 mmol), CuBr (21 mg, 0.15 mmol) and pyridine (36 mg, 36 µl, 0.45 mmol), and the mixture was stirred under air at 70°C overnight. The toluene was then removed under low pressure, and the crude was chromatographed (DCM, EtOAc 0-10%) to yield diethyl 2,2'-diamino-[1,1'-biazulene]-3,3'-dicarboxylate **5** as a red solid (214 mg, 0.50 mmol). Yield: 41%

^{1}H-NMR (500 MHz, CD_2Cl_2) δ 9.03 (d, J = 9.8 Hz, 1H), 7.47 (dd, J = 10.4, 1.0 Hz, 2H), 7.42 (td, J = 10.1, 1.2 Hz, 2H), 7.31 (tt, J = 10.2, 1.1 Hz, 2H), 7.16 (ddd, J = 10.2, 9.1, 1.1 Hz, 2H), 4.47 (q, J = 7.1 Hz, 4H), 1.49 (t, J = 7.1 Hz, 6H).

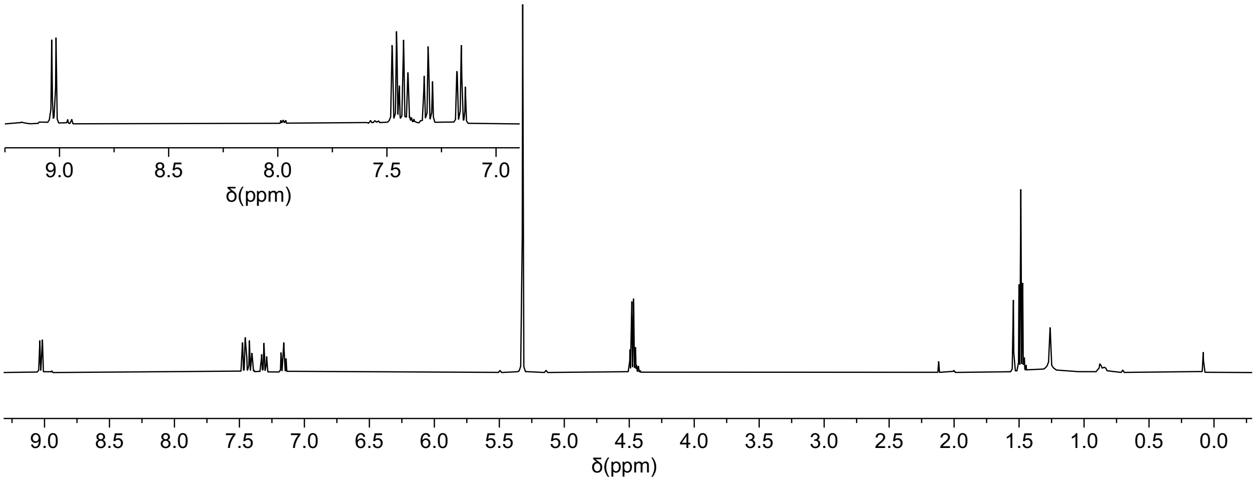

Figure S4: ^{1}H NMR (500 MHz, 298 K, CD_2Cl_2) spectrum of **5**. The aromatic region is presented in the insert.

[1,1'-biazulene]-2,2'-diamine (**6**):

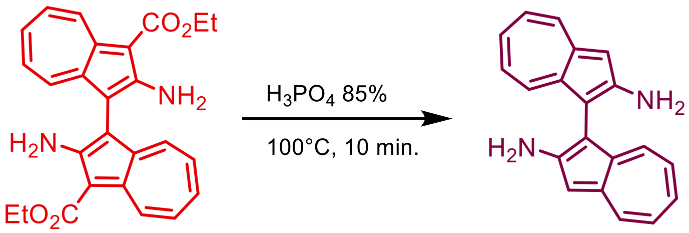

5 **6**

Scheme S6: Synthesis of compound **6**.

Diethyl 2,2'-diamino-[1,1'-biazulene]-3,3'-dicarboxylate **5** (300 mg, 0.70 mmol) was added to a round-bottom flask containing 10 ml of H_3PO_4 85%. The flask was then added to a pre-heated oil bath at 100°C, and the mixture was stirred 10 minutes. Gas evolution was observed. Afterward, the solution was left to cool down to RT, and was then added to 50 ml of water. The resulting solution was neutralised with NaOH and extracted with EtOAc. The organic layer was dried with $MgSO_4$ and the solvent was removed under low pressure to yield [1,1'-biazulene]-2,2'-diamine **6** ("**BAAZU**") as a dark brown product (181 mg, 0.64 mmol). Yield: 90%. The compound was found to slowly degrade over time when exposed to air, even at 4°C.

^{1}H-NMR (600 MHz, DMSO-d_6) δ 7.80 (dd, J = 9.5, 1.3 Hz, 2H), 7.20 (d, J = 9.8 Hz, 2H), 7.10 – 7.01 (m, 4H), 6.91 (ddd, J = 10.5, 8.7, 1.4 Hz, 2H), 6.77 (s, 2H), 6.01 (s, 4H).

^{13}C-NMR (126 MHz, DMSO-d_6) δ 158.05, 142.62, 139.18, 128.14, 126.53, 125.75, 124.36, 123.90, 107.86, 102.82.

ESI-MS (pos. mode): calculated for $[C_{20}H_{16}N_2+H]^+$: 285.1386, found 285.1376

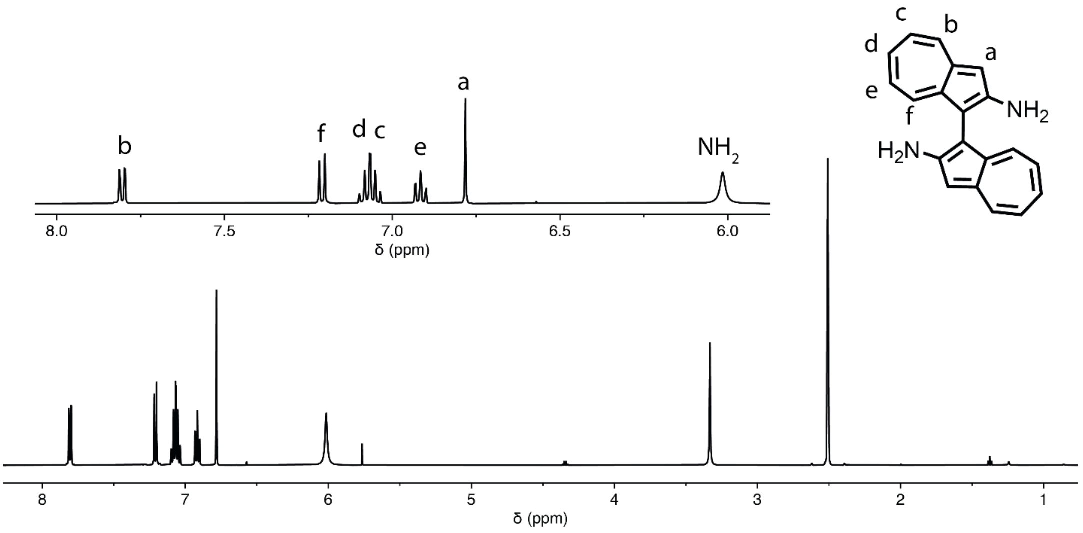

Figure S5: ^{1}H NMR (600 MHz, 298 K, DMSO-d_6) spectrum of **6**.

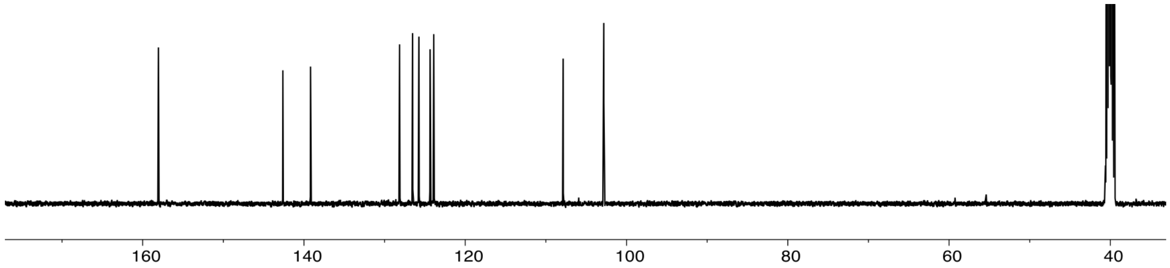

Figure S6: ^{13}C-NMR (126 MHz, 298 K, DMSO-d_6) spectrum of **6**.

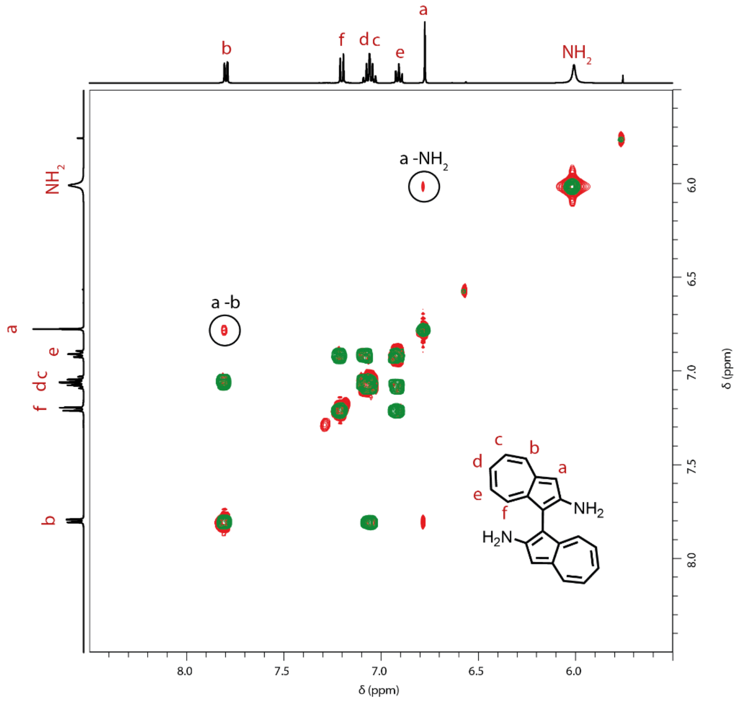

Figure S7: ^{1}H-^{1}H COSY (green traces) and NOESY (red traces) NMR (600 MHz, 298 K, DMSO-d_6) spectra **6**. Important NOE correlations are highlighted in the figure.

1.2. Synthesis of 3,3'-di(pyridin-3-yl)-[1,1'-biazulene]-2,2'-diamine ("BAAZU-1")

General scheme

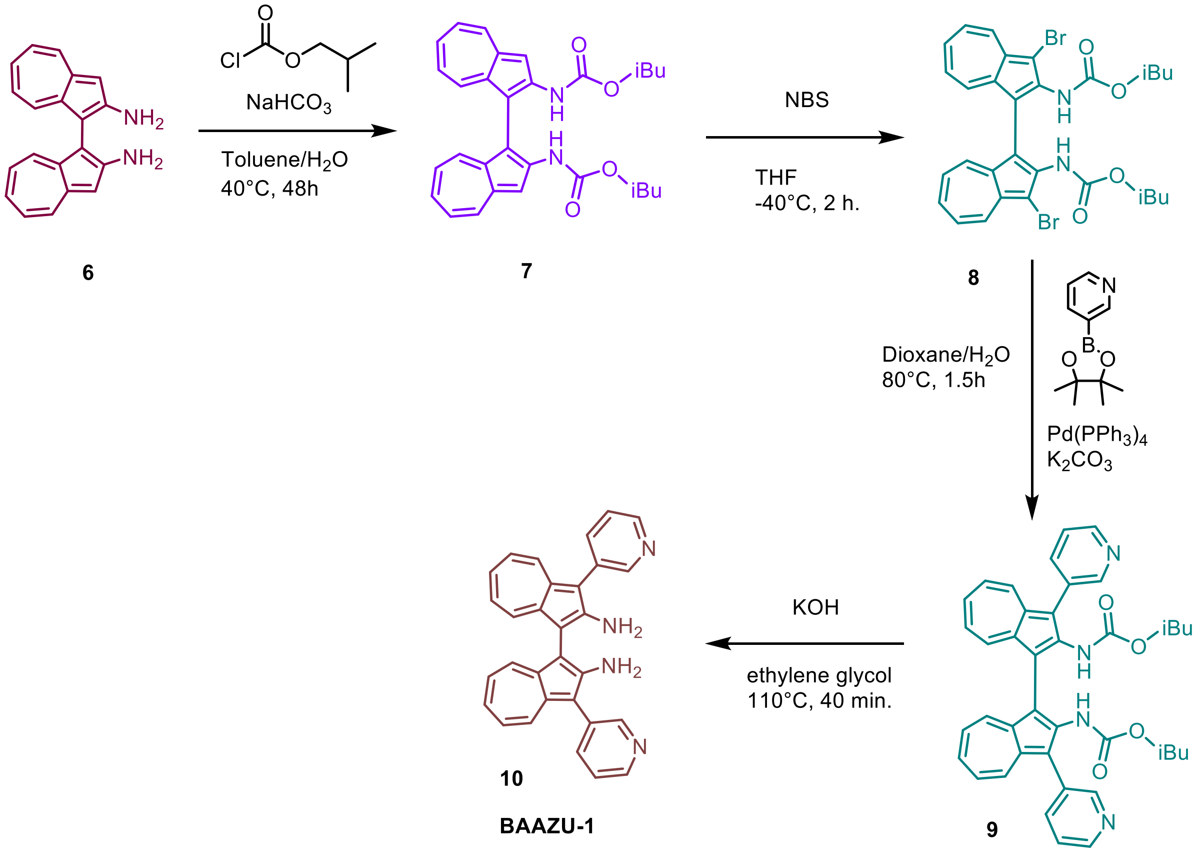

Scheme S7: Synthesis of compounds **7** to **10** ("**BAAZU-1**").

<u>Diisobutyl [1,1'-biazulene]-2,2'-diyldicarbamate (**7**):</u>

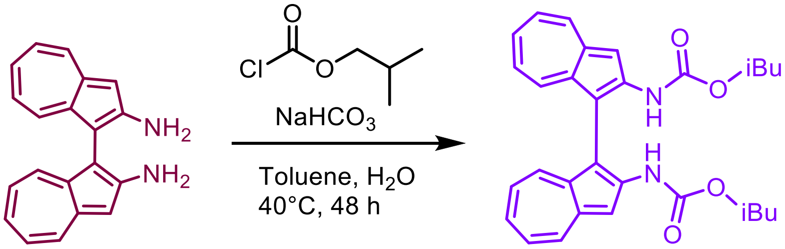

6 **7**

Scheme S8: Synthesis of compound **7**.

BAAZU (**6**) (455mg, 1.6mmol) was dissolved in a mixture of toluene (50 ml) and water (20 ml) with $NaHCO_3$ (1.1 g, 13.4 mmol) under argon. Isobutyl chloroformate (1.74 ml, 13.4 mmol) was slowly added as portions over the course of the reaction, and the reaction mixture was stirred strongly at 40°C for 48h. The solvent was then evaporated under low pressure, and the crude was chromatographed on silica gel (DCM). The

resulting product was suspended in a 1:1 mixture of DCM and pentane and filtered to yield a purple powder as the title compound **7** (400mg, 0.82mmol, 52% yield).

^{1}H NMR (600 MHz, $CDCl_3$) δ 8.26 (d, *J* = 9.6 Hz, 2H), 7.96 (s, 2H), 7.59 (d, *J* = 9.8 Hz, 2H), 7.43 (tt, *J* = 9.8, 1.0 Hz, 2H), 7.25 (t, *J* = 9.8 Hz, 2H), 7.04 (t, *J* = 9.7 Hz, 2H), 6.83 (s, 2H), 3.97 – 3.81 (m, 4H), 1.86 (s, 2H), 0.83 (d, *J* = 4.7 Hz, 12H).

^{13}C NMR (151 MHz, $CDCl_3$) δ 153.46, 145.62, 141.69, 137.91, 134.42, 133.92, 132.01, 125.09, 124.69, 105.87, 27.85, 19.04. One nucleus missing.

ESI-MS (pos. mode): calculated for $[C_{30}H_{32}N_2O_4+H]^+$: 485.2435, found 485.2437

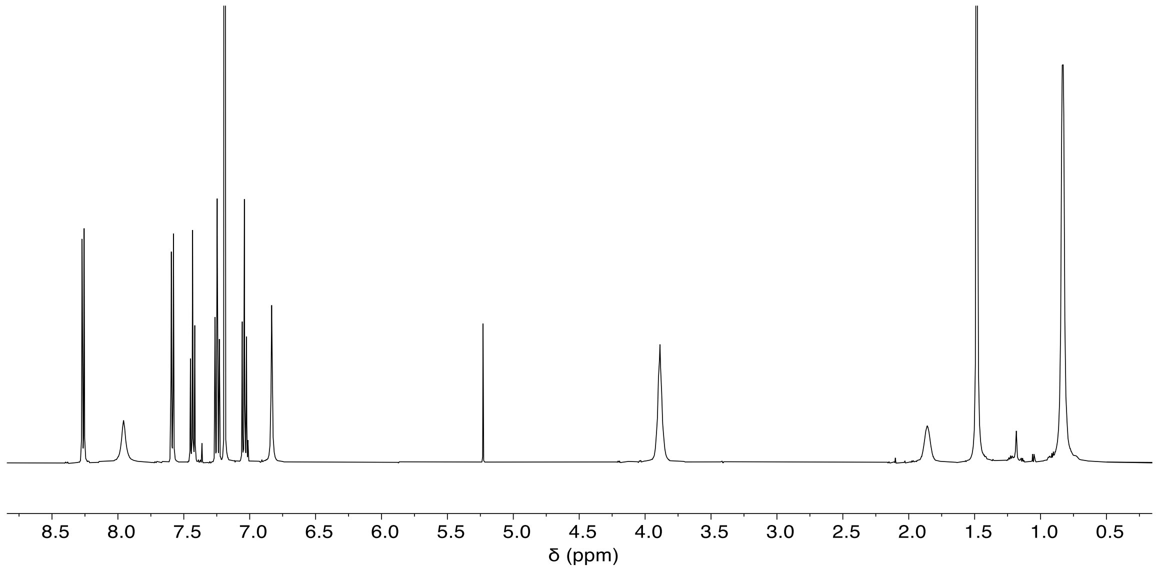

Figure S8: ^{1}H-NMR (600 MHz, 298 K, $CDCl_3$) spectrum of **7**.

Diisobutyl (3,3'-dibromo-[1,1'-biazulene]-2,2'-diyl)dicarbamate (**8**):

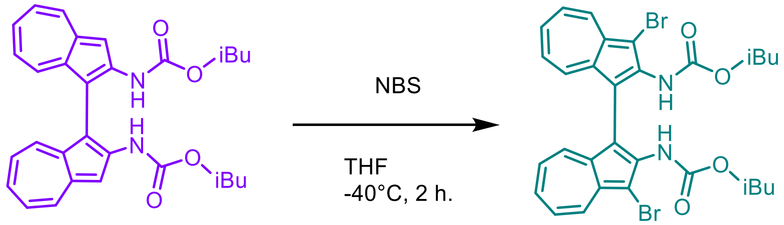

7 **8**

Scheme S9: Synthesis of compound **8**.

7 (400 mg, 0.83 mmol) was added to a two-necked round-bottom flask with 10 ml THF. The system was flushed with argon and then cooled to -40°C. A solution of NBS (310 mg, 1.74 mmol) in 10 ml THF was slowly dropped into the flask, and the reaction mixture was left to stir for two hours. Afterward, it was brought to room temperature, water was added to the mixture, and the aqueous phase was extracted with DCM. The organic phase was then dried with $MgSO_4$ and evaporated under reduced pressure. The

crude was chromatographed on silica (pentane, EtOAc 20%) to obtain the title compound as a blue-grey powder (343 mg, 0.53 mmol). Yield: 64%

It is worth noting that a similar reaction with NIS was attempted, but failed to produce the diiodo derivative.

^{1}H NMR (600 MHz, $CDCl_3$) δ 8.42 (dd, *J* = 9.9, 1.0 Hz, 2H), 7.66 (dd, *J* = 9.8, 1.0 Hz, 2H), 7.57 (tt, *J* = 9.9, 1.0 Hz, 2H), 7.34 (td, *J* = 9.8, 0.8 Hz, 2H), 7.07 (t, *J* = 9.7 Hz, 2H), 6.94 (s, 2H), 3.81 – 3.72 (m, 4H), 1.74 (hept, *J* = 6.7 Hz, 2H), 0.75 (dd, *J* = 6.7, 2.4 Hz, 12H).

^{13}C NMR (151 MHz, $CDCl_3$) δ 153.68, 142.82, 138.34, 137.57, 135.90, 134.94, 134.84, 125.08, 124.94, 115.29, 99.21, 71.99, 28.03, 19.05.

ESI-MS (pos. mode): calculated for $[C_{30}H_{30}Br_2N_2O_4+H]^+$: 663.0465, found 663.0455.

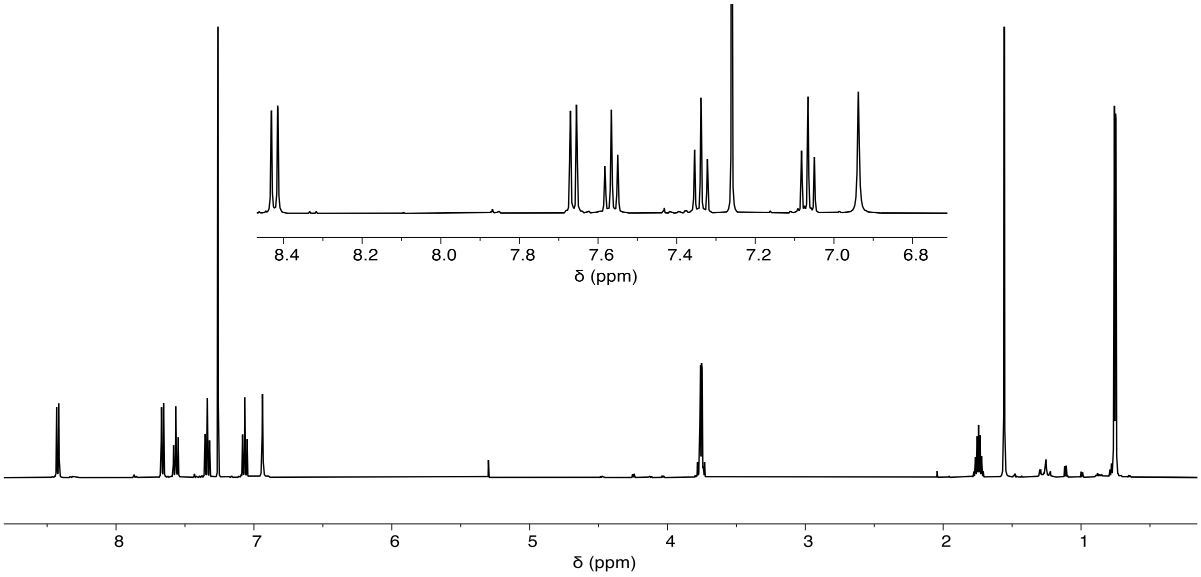

Figure S9: ^{1}H-NMR (500 MHz, 298 K, $CDCl_3$) spectrum of **8**. The aromatic region is presented in the insert.

<u>Diisobutyl (3,3'-di(pyridin-3-yl)-[1,1'-biazulene]-2,2'-diyl)dicarbamate (**9**):</u>

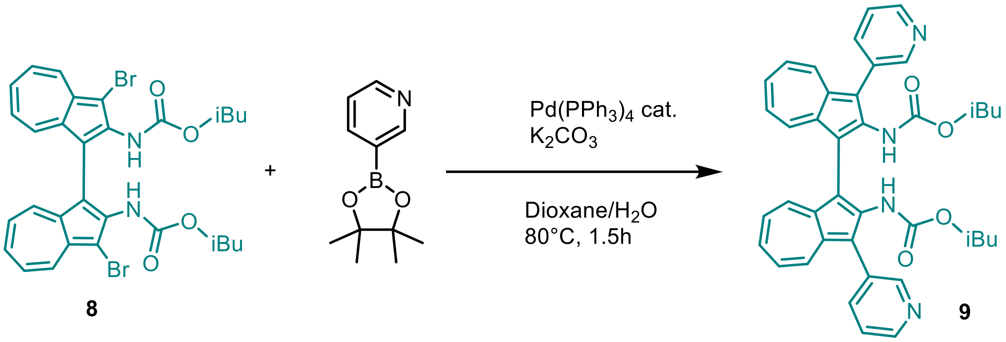

Scheme S10: Synthesis of compound **9**.

In a Schlenk flask, **8** (100 mg, 0.156 mmol), 3-pyridine-BPin (128 mg, 0.62 mmol), and K_2CO_3 (86 mg, 0.62 mmol) were added to a mixture of 1,4-dioxane (20 ml) and water (5 ml). The solution was degassed by

bubbling argon though it for 30 min. $Pd(PPh_3)_4$ (17.3 mg, 0.015 mmol) was added as the catalyst and the flask was closed. The reaction mixture was heated at 80°C and stirred for 1.5 hours. Afterward, the reaction was quenched with water and the product was extracted with DCM. The organic phased was dried with $MgSO_4$ and evaporated under lower pressure. The crude was chromatographed on silica (EtOAc, MeOH 0-5%) to yield the crude title compound **9** as a blue-grey powder (76 mg, 0.12 mmol), which was used in the next step without further purification.

ESI-MS (pos. mode): calculated for $[C_{40}H_{38}N_4O_4]^+$: 639.2966, found 639.2942

<u>3,3'-Di(pyridin-3-yl)-[1,1'-biazulene]-2,2'-diamine (**BAAZU-1**):</u>

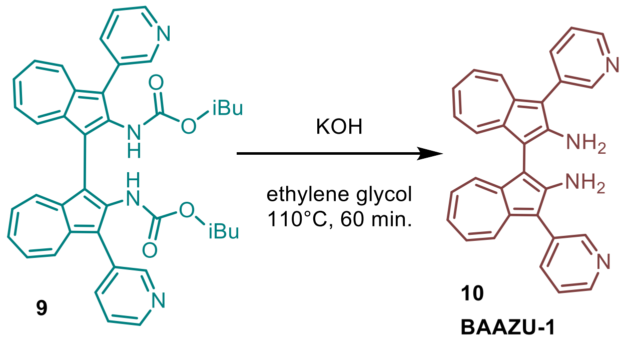

Scheme S11: Synthesis of compound **10** ("**BAAZU-1**").

9 (70 mg, 0.11 mmol) was added to a flask with ethylene glycol (10 ml) and powdered KOH (200 mg, excess), and argon was bubbled through the mixture for 15 minutes. Afterward, the flask was stoppered, and the mixture was stirred and heated at 110°C for 40 minutes. It was then quenched with water and the crude was extracted with DCM. The crude was chromatographed on silica (DCM, 5% MeOH) to yield **10** as a brown powder. The product was further purified by recycling GPC ($CHCl_3$, 0.5% NEt_3) to yield **BAAZU-1** as a brown powder (36.4 mg, 0.083 mmol, 75%)

ESI-MS (pos. mode): calculated for $[C_{30}H_{22}N_4]^+$: 438.1839, found 438.1826

^{1}H-NMR (600 MHz, CD_3CN) δ 8.84 (dd, J = 2.3, 0.9 Hz, 2H), 8.60 (dd, J = 4.8, 1.7 Hz, 2H), 8.00 (ddd, J = 7.8, 2.3, 1.7 Hz, 2H), 7.92 (dd, J = 9.9, 0.9 Hz, 2H), 7.57 – 7.49 (m, 4H), 7.27 (tt, *J* = 9.6, 1.0 Hz, 2H), 7.16 (td, J = 9.8, 1.0 Hz, 2H), 7.07 (td, J = 9.7, 1.0 Hz, 2H), 5.16 (s, 4H)

^{13}C-NMR (151 MHz, CD_3CN) δ 155.10, 151.48, 148.02, 139.75, 139.67, 138.07, 131.99, 130.77, 128.00, 126.20, 125.86, 125.21, 124.49, 110.98, 107.14

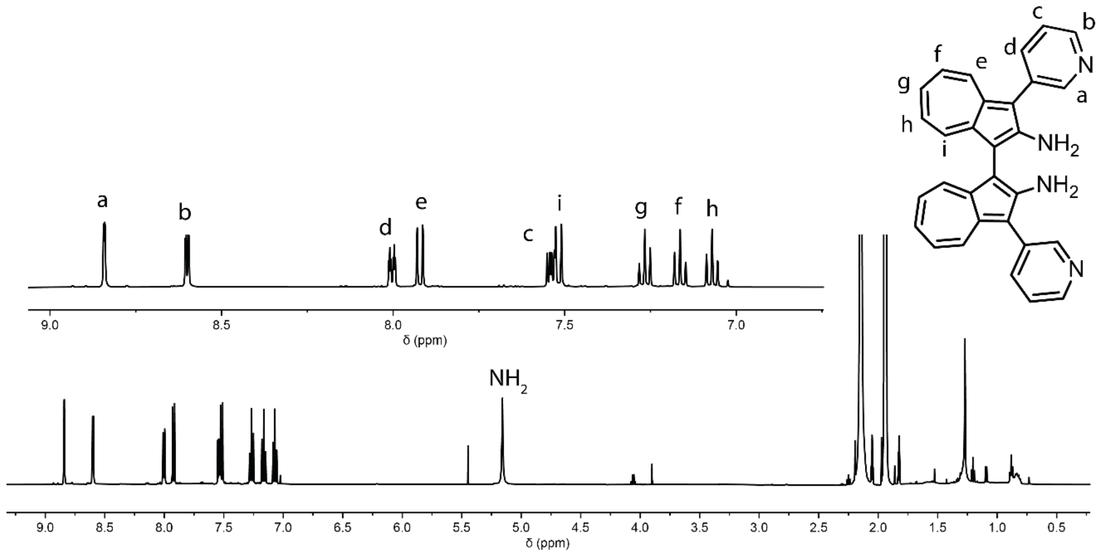

Figure S10: ^{1}H-NMR (600 MHz, 298 K, DMSO-d_6) spectrum of **BAAZU-1** (**10**). The aromatic region is presented in the insert.

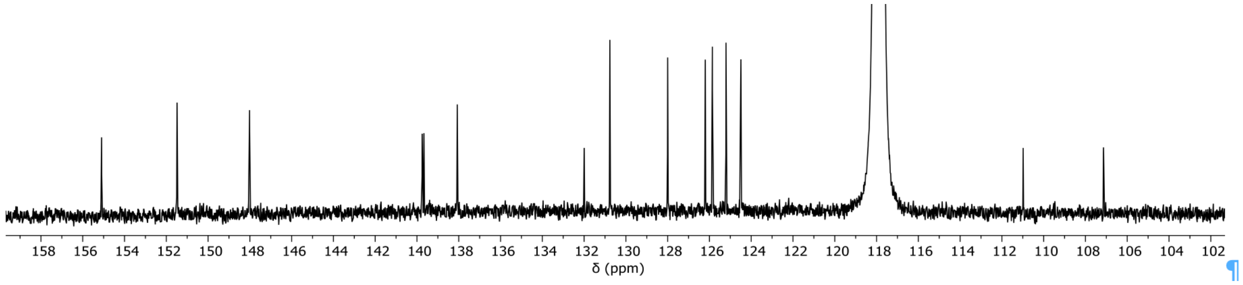

Figure S11: ^{13}C-NMR (151 MHz, 298 K, DMSO-d_6) spectrum of **BAAZU-1** (**10**).

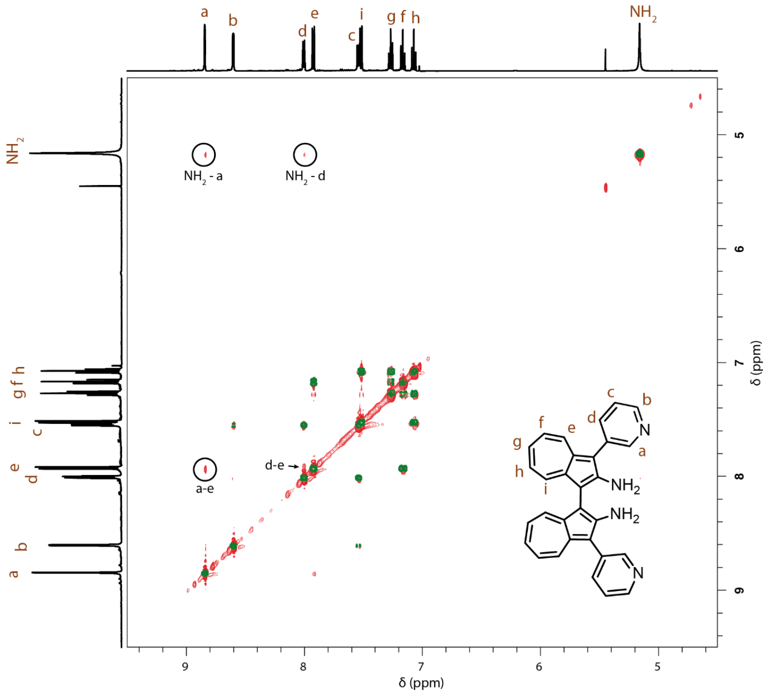

Figure S12: ^{1}H-^{1}H COSY (green traces) and NOESY (red traces) NMR (600 MHz, 298 K, DMSO-d_6) spectra of **BAAZU-1** (**10**). Important NOE correlations are highlighted in the figure.

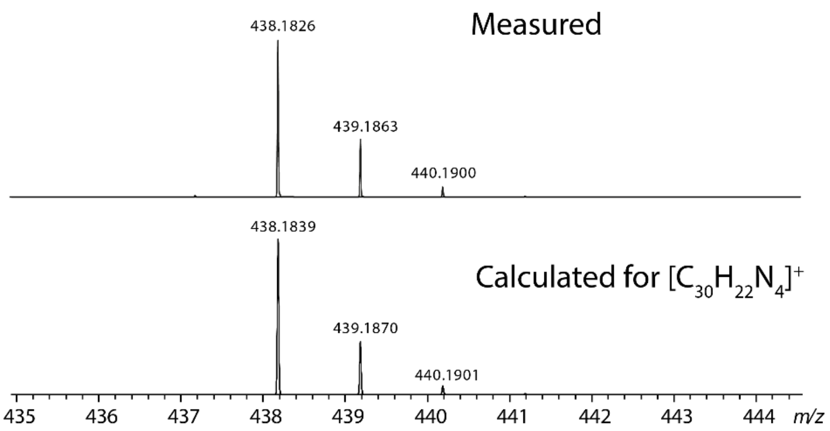

Figure S13: ESI-MS spectrum of [**BAAZU-1**]$^+$.

1.3. Synthesis of 3,3'-di(isoquinolin-7-yl)-[1,1'-biazulene]-2,2'-diamine ("BAAZU-2")

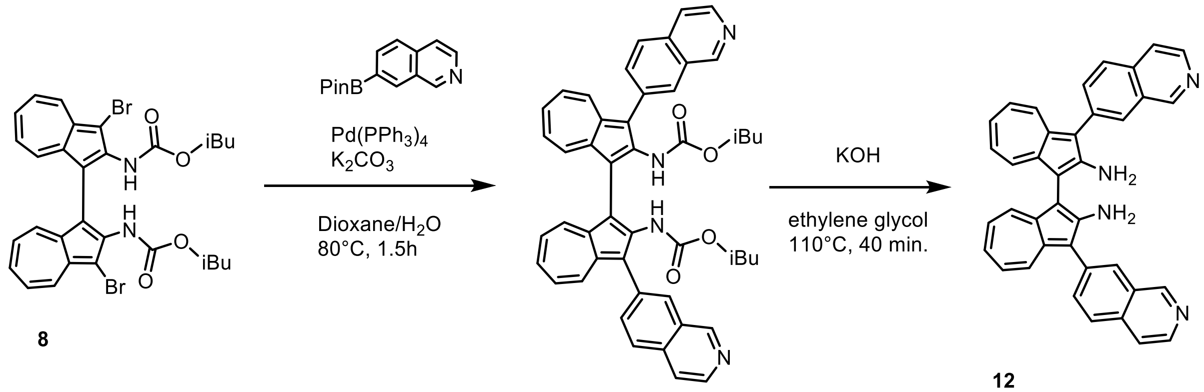

Scheme S12: Synthesis of compounds **11** and **12** ("**BAAZU-2**").

Diisobutyl (3,3'-di(isoquinolin-7-yl)-[1,1'-biazulene]-2,2'-diyl)dicarbamate (**11**):

In a Schlenk flask, **8** (170 mg, 0.260 mmol), isoquinoline-7-boronic acid pinacol ester (230 mg, 0.9 mmol), and K_2CO_3 (166 mg, 1.2 mmol) were added to a mixture of 1,4-dioxane (20 ml) and water (5 ml). The solution was degassed by bubbling argon though it for 30 min. $Pd(PPh_3)_4$ (35 mg, 0.03 mmol) was added as the catalyst and the flask was closed. The reaction mixture was heated at 80°C and stirred for 1.5 hours. Afterward, the reaction was quenched with water and the product was extracted with DCM. The organic phase was dried with $MgSO_4$ and evaporated under lower pressure. The crude was chromatographed on silica (DCM, MeOH 1-3%) to yield the title compound **11** as a blue-grey powder (173 mg, 0.234 mmol, 90% yield).

^{1}H NMR (500 MHz, CD_2Cl_2) δ 9.26 (s, 2H), 8.50 (d, *J* = 5.7 Hz, 2H), 8.43 (dd, *J* = 10.0, 0.9 Hz, 2H), 8.16 (d, *J* = 1.2 Hz, 2H), 7.99 (d, *J* = 1.2 Hz, 4H), 7.85 (dd, *J* = 9.8, 1.0 Hz, 2H), 7.75 – 7.69 (m, 2H), 7.58 (tt, *J* = 9.8, 1.1 Hz, 2H), 7.33 – 7.21 (m, 4H), 7.12 (t, *J* = 9.7 Hz, 2H), 5.33 – 5.29 (m, 2H), 3.54 (dd, *J* = 10.5, 6.8 Hz, 2H), 3.45 (dd, *J* = 10.6, 6.8 Hz, 2H), 1.53 (hept, *J* = 6.7 Hz, 2H), 0.61 (dd, *J* = 6.7, 2.1 Hz, 12H).

^{13}C-NMR (126 MHz, CD_2Cl_2) δ 153.75, 152.42, 143.47, 142.85, 138.13, 137.12, 137.05, 135.05, 134.59, 134.47, 133.88, 132.94, 129.05, 127.90, 126.58, 125.05, 124.65, 121.80, 120.19, 115.28, 71.28, 27.77, 18.45, 18.43.

ESI-MS (pos. mode): calculated for $[C_{48}H_{42}N_4O_4+H]^+$: 739.3279, found 739.3198

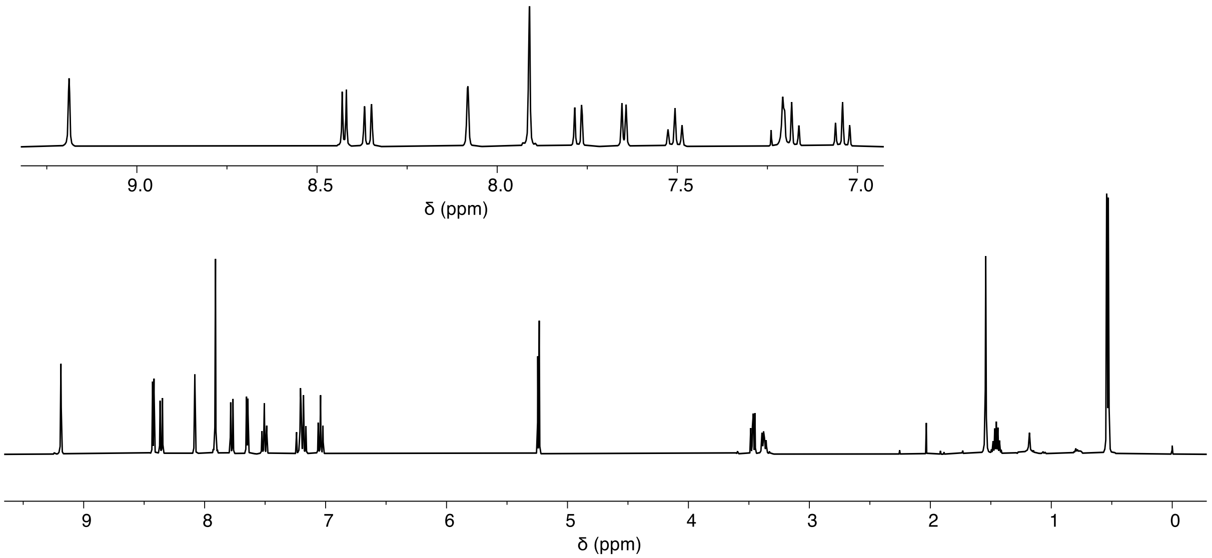

Figure S14: ^{1}H-NMR (500 MHz, 298 K, CD_2Cl_2) spectrum of **11**. The aromatic region is presented in the insert.

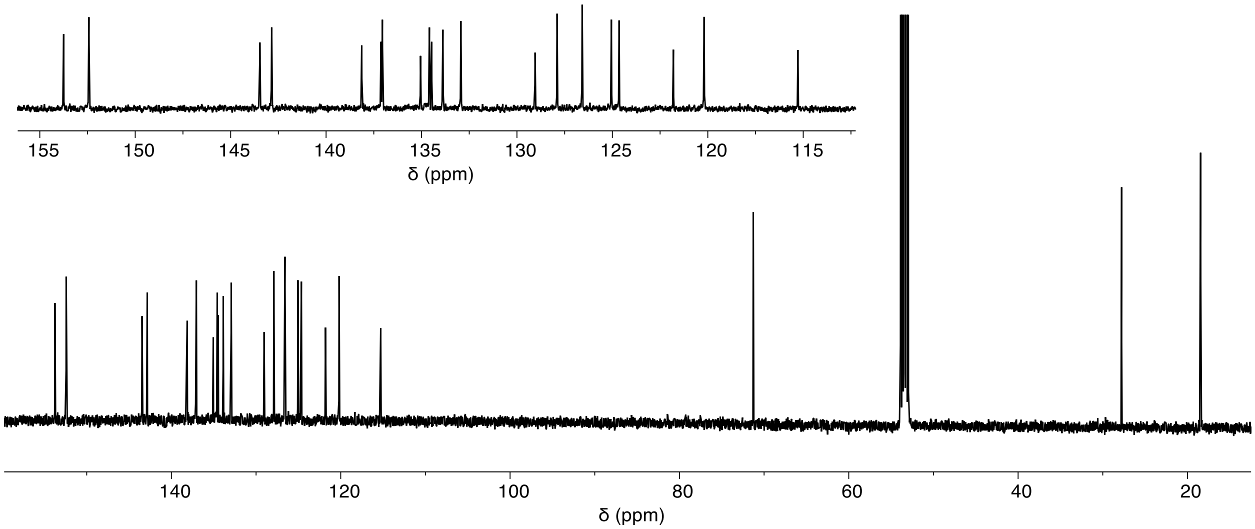

Figure S15: ^{13}C-NMR (151 MHz, 298 K, DMSO-d_6) spectrum of **11**. The aromatic region is presented in the insert.

3,3'-Di(isoquinolin-7-yl)-[1,1'-biazulene]-2,2'-diamine ("**BAAZU-2**"):

11 (62 mg, 0.08 mmol) was added to a flask with ethylene glycol (10 ml) and powdered KOH (200 mg, excess), and argon was bubbled through the mixture for 15 minutes. Afterward, the flask was stoppered, and the mixture was stirred and heated at 110°C for 40 minutes. It was then quenched with water and the crude was extracted with DCM. The crude was chromatographed on silica (DCM, 5% MeOH) to yield the title compound as a brown powder. The product was further purified by recycling GPC ($CHCl_3$, 0.5% NEt_3) to yield **BAAZU-2** (37 mg, 0.069 mmol, yield 86%)

^{1}H-NMR (500 MHz, DMSO-d_6) δ 9.47 (s, 2H), 8.60 (d, *J* = 5.6 Hz, 2H), 8.40 (s, 2H), 8.21 (d, *J* = 8.5 Hz, 2H), 8.14 (dd, *J* = 8.4, 1.7 Hz, 2H), 8.01 (dd, *J* = 9.8, 1.1 Hz, 2H), 7.98 (d, *J* = 5.7 Hz, 2H), 7.51 (dd, *J* = 9.9, 0.8 Hz, 2H), 7.31 – 7.24 (m, 2H), 7.21 (td, *J* = 9.8, 1.2 Hz, 2H), 7.12 (td, *J* = 9.6, 1.2 Hz, 2H), 6.05 (s, 4H).

^{13}C-NMR (126 MHz, DMSO-d_6) δ 155.46, 152.94, 142.97, 139.22, 139.11, 134.87, 134.21, 133.84, 129.74, 129.39, 128.38, 127.24, 127.00, 125.64, 125.08, 124.94, 120.67, 113.10, 107.01.

ESI-MS (pos. mode): calculated for $[C_{38}H_{26}N_4]^+$: 538.2152, found 538.2156

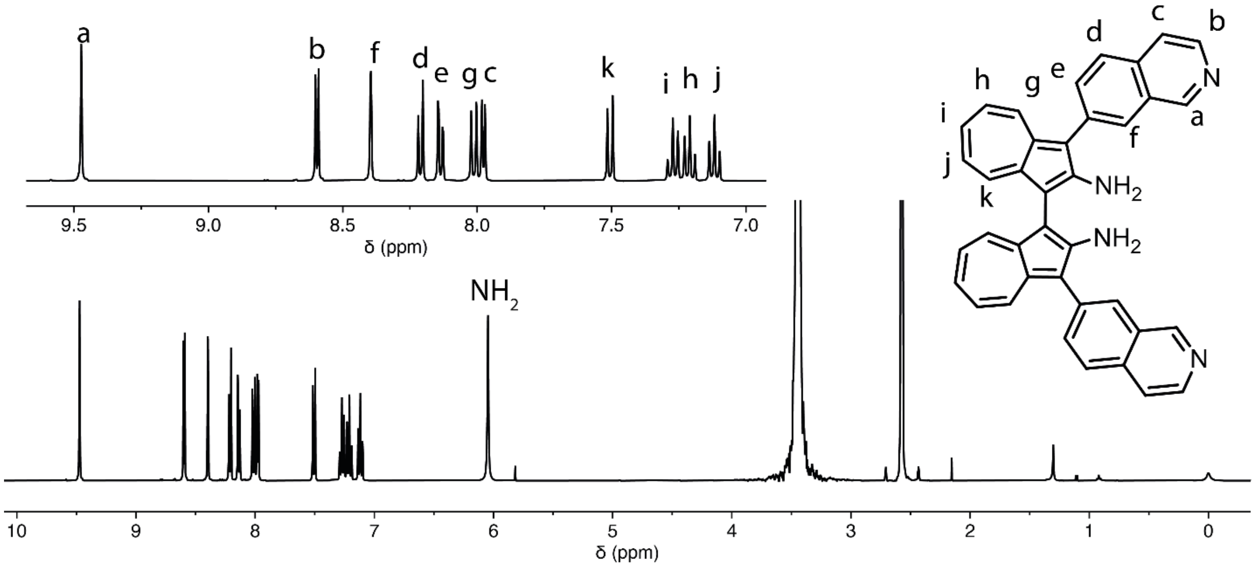

Figure S16: ^{1}H-NMR (500 MHz, 298 K, DMSO-d_6) spectrum of **BAAZU-2** (**12**). The aromatic region is presented in the insert.

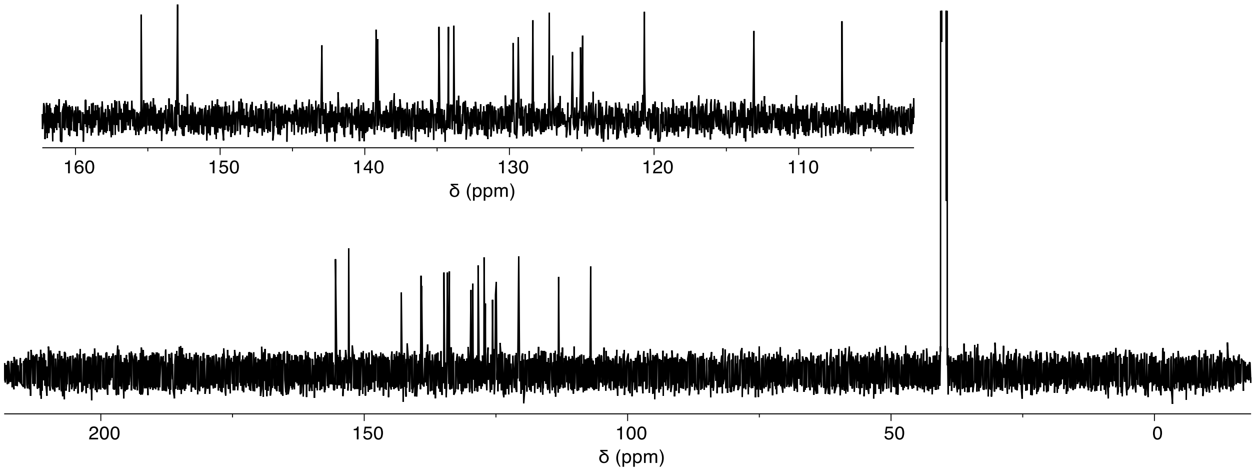

Figure S17: ^{13}C-NMR (151 MHz, 298 K, DMSO-d_6) spectrum of **BAAZU-2** (**12**). The aromatic region is presented in the insert.

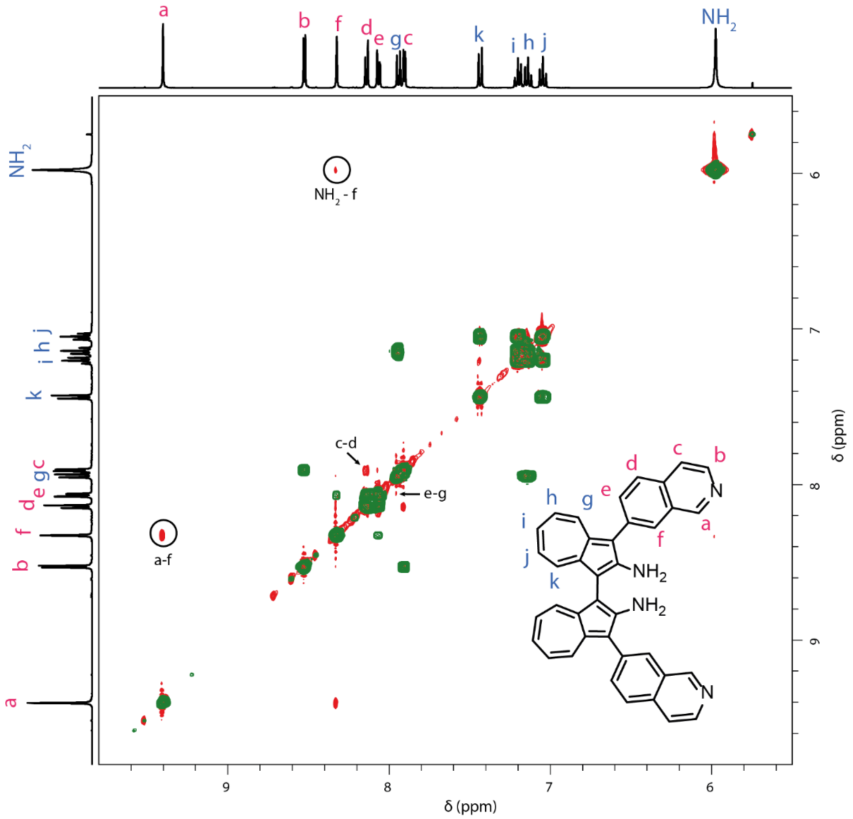

Figure S18: ^1H-^1H COSY (green traces) and NOESY (red traces) NMR (600 MHz, 298 K, DMSO-d_6) spectra of **BAAZU-2** (**12**). Important NOE correlations are highlighted in the figure.

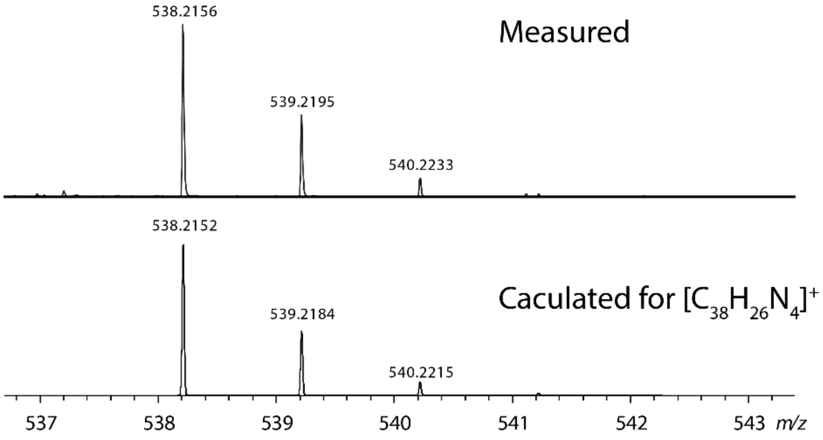

Figure S19: ESI-MS spectrum of [**BAAZU-2**]$^+$.

1.4. $[Pd(CD_3CN)_4](BArF_{20})_2$

A solution of $Pd(CH_3CN)_2Cl_2$ (15 µmol, 3.89 mg) and $[Ag(CH_3CN)_2](BArF_{20})$ (prepared from metal metathesis from $LiBArF_{20}$ and $AgNO_3$ in acetonitrile, followed by recrystallisation from diethylether)[5,6] (30 µmol, 28.53 mg) in CD_3CN (1 ml) was prepared, and it was stirred overnight protected from the light. Afterward, the AgCl precipitate was filtered off to yield a 15 mM solution of $[Pd(CD_3CN)_4](BArF_{20})_2$. The solution was used as-is for the formation of the cages.

2. Chiral separation of the ligands by HPLC

Chiral high-performance liquid chromatography was performed on an Agilent Technologies 1260 infinity HPLC system equipped with Daicel CHIRALPAK IC columns [250 x 4.6 mm (analytic) and 250 x 10 mm (semipreparative)]. Flowrates were set up at 1 ml/min for analytics and 4 ml/min for separation.

Intermediate **8** was separated using a dichloromethane/hexane (70%/30%) mixture as eluent.

BAAZU-1 was separated using a dichloromethane (with 0.05% NEt_3)/isopropanol (97%/3%) mixture as eluent.

BAAZU-2 was separated using a dichloromethane (with 0.05% NEt_3)/ isopropanol (92.5%/7.5%) mixture as eluent. The full method is presented on the following table:

Table S1: Chiral HPLC method for the separation of the two enantiomers of **BAAZU-2**.

Time [min]	DCM (%)	iPrOH (%)	MeOH (%)	n-hexane (%)
0.00	92.5	7.5	0	0
20.00	92.5	7.5	0	0
20.01	0	0	80	20
30.00	0	0	80	20
30.01	92.5	7.5	0	0
40.00	92.5	7.5	0	0

The washing step with methanol and n-hexane was necessary to keep the enantiomers eluting at similar times upon repeated runs. Without the washing step, the peaks would gradually elute earlier and earlier, and overlap after a certain number of cycles.

Chromatograms of **8**:

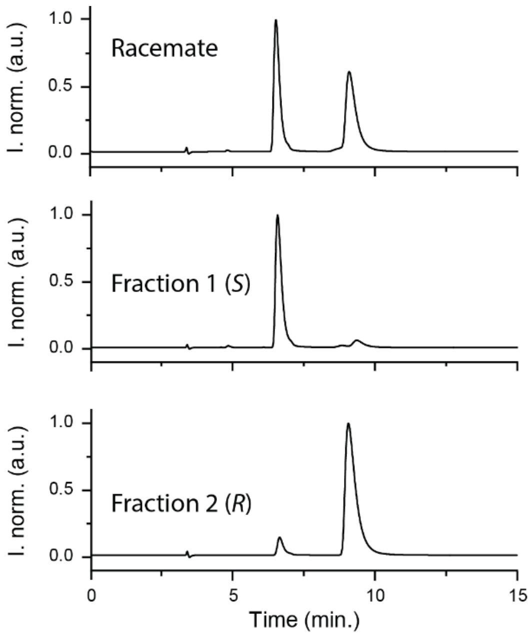

Figure S20: Chiral HPLC chromatograms of intermediate **8** as racemate (top), of the first fraction (middle), and of the second fraction (bottom).

Chromatograms of **BAAZU-1**:

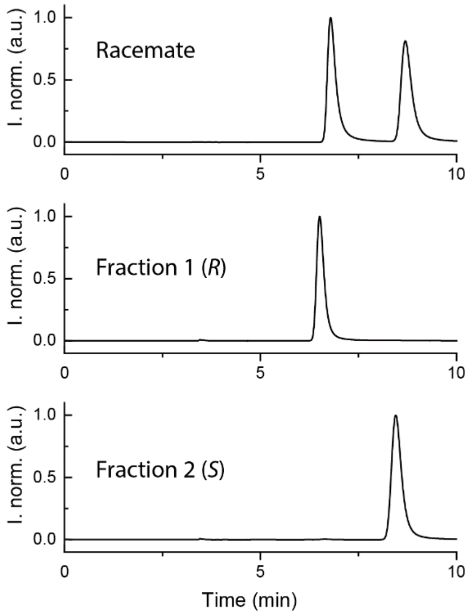

Figure S21: Chiral HPLC chromatograms of **BAAZU-1** as racemate (top), of the first fraction (middle), and of the second fraction (bottom).

Chromatograms of **BAAZU-2**:

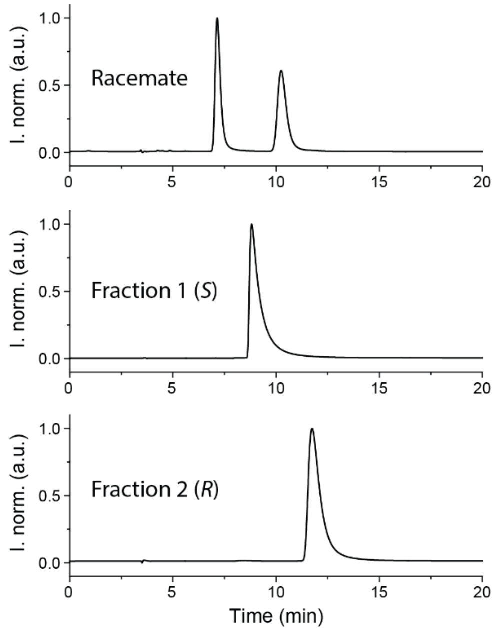

Figure S22: Chiral HPLC chromatograms of **BAAZU-2** as racemate (top), of the first fraction (middle), and of the second fraction (bottom).

The chromatograms of the racemates show different retention times compared to the separated enantiomers. The reason is not clear to us. However, the washing step explained above allows us to keep more consistent retention times in-between runs.

3. Preparation of the cages

All of the Pd_2L_4 species were assembled according to the same procedure: to 450µl of a 3.11mM solution of the ligand in CD_3CN, DMSO-d_6, or CD_3NO_2, 50µl of 15mM solution of the Pd(II) salt in the same solvent were added. The solutions were then heated at 70°C.

3.1. Pd_2**BAAZU-1**$^{rac}{}_4$ from the racemic ligand

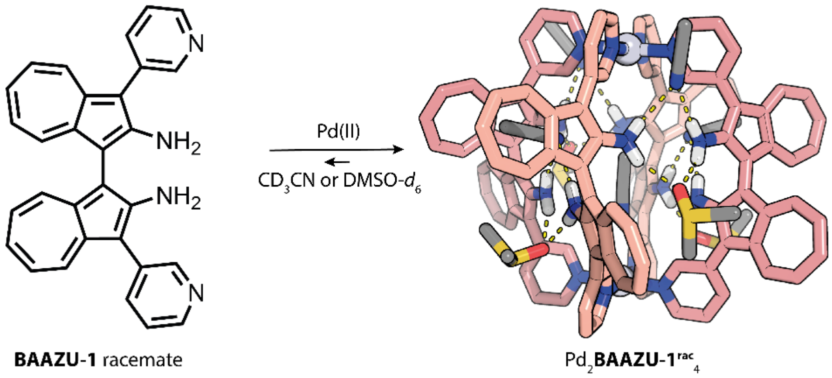

Scheme S13: Formation of Pd_2**BAAZU-1**$^{rac}{}_4$ from the racemate of **BAAZU-1**.

The coordination cage [Pd_2**BAAZU-1**$^{rac}{}_4$]$(BF_4)_4$ was synthesised as a 0.7mM solution in CD_3CN, DMSO-d_6, or CD_3NO_2 according to the general procedure, using $[Pd(CH_3CN)_4](BF_4)_4$ as the palladium(II) salt and the racemate of **BAAZU-1**. The *meso-trans* cage was obtained after heating for 24 hours in CD_3CN, or for 5 hours in DMSO-d_6. Only a mixture of diastereomers was obtained from CD_3NO_2. The following characterisation was made from the CD_3CN solution.

^{1}H-NMR (600 MHz, CD_3CN) δ 9.53 (dd, *J* = 5.7, 1.1 Hz, 2H), 9.46 (s, 2H), 8.31 (ddd, *J* = 7.8, 2.0, 1.4 Hz, 2H), 7.97 – 7.90 (m, 4H), 7.23 – 7.17 (m, 2H), 7.18 (dd, *J* = 9.4, 0.9 Hz, 2H), 7.11 (t, *J* = 9.7 Hz, 2H), 6.94 (ddd, *J* = 10.2, 9.1, 0.9 Hz, 2H), 5.30 (s, 4H).

^{13}C-NMR (151 MHz, CD_3CN) δ 156.15, 151.01, 148.24, 142.60, 141.42, 139.74, 137.14, 132.70, 129.05, 128.74, 127.58, 126.98, 126.86, 118.26, 108.51, 108.02, 55.28.

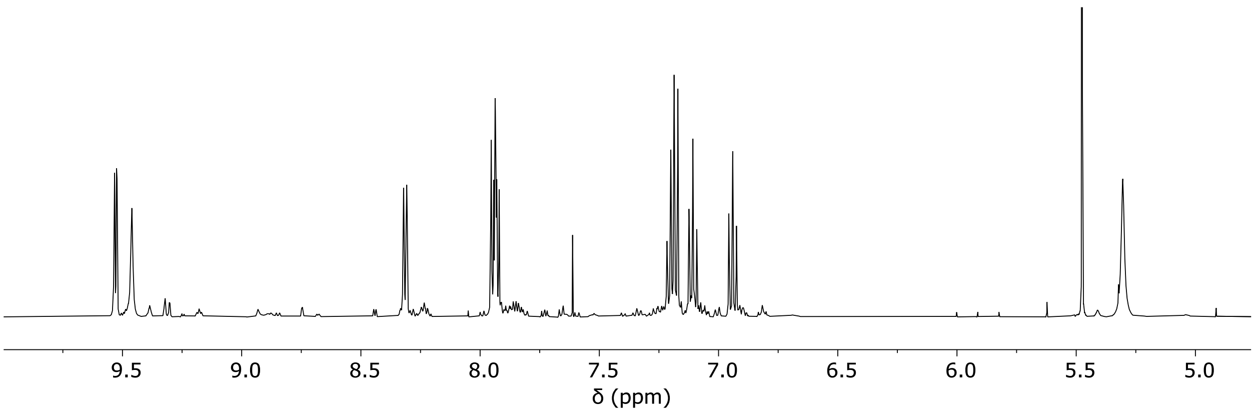

Figure S23: ^{1}H-NMR (600 MHz, 298 K, CD_3CN) spectrum of [Pd_2**BAAZU-1**$^{rac}{}_4$]$(BF_4)_4$.

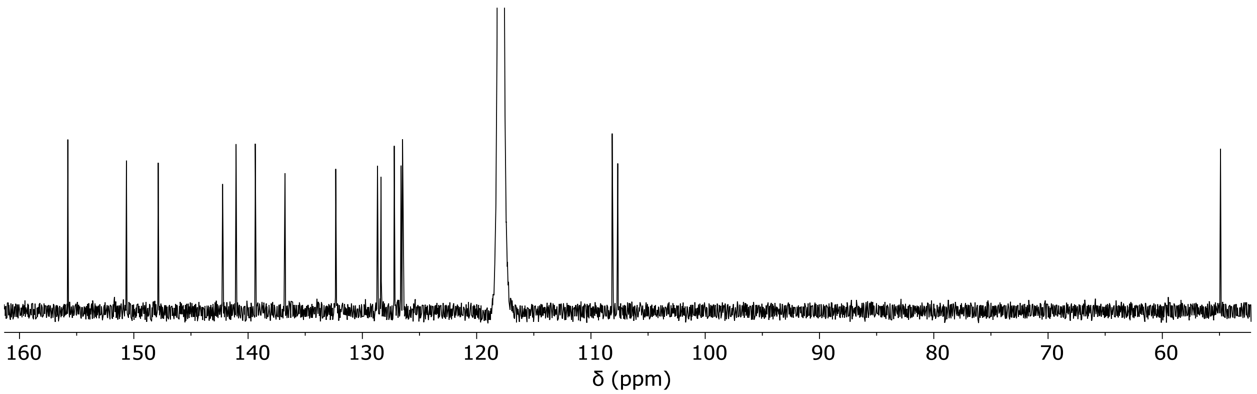

Figure S24: ^{13}C-NMR (151 MHz, 298 K, CD_3CN) spectrum of [Pd_2**BAAZU-1**$^{rac}{}_4$]$(BF_4)_4$.

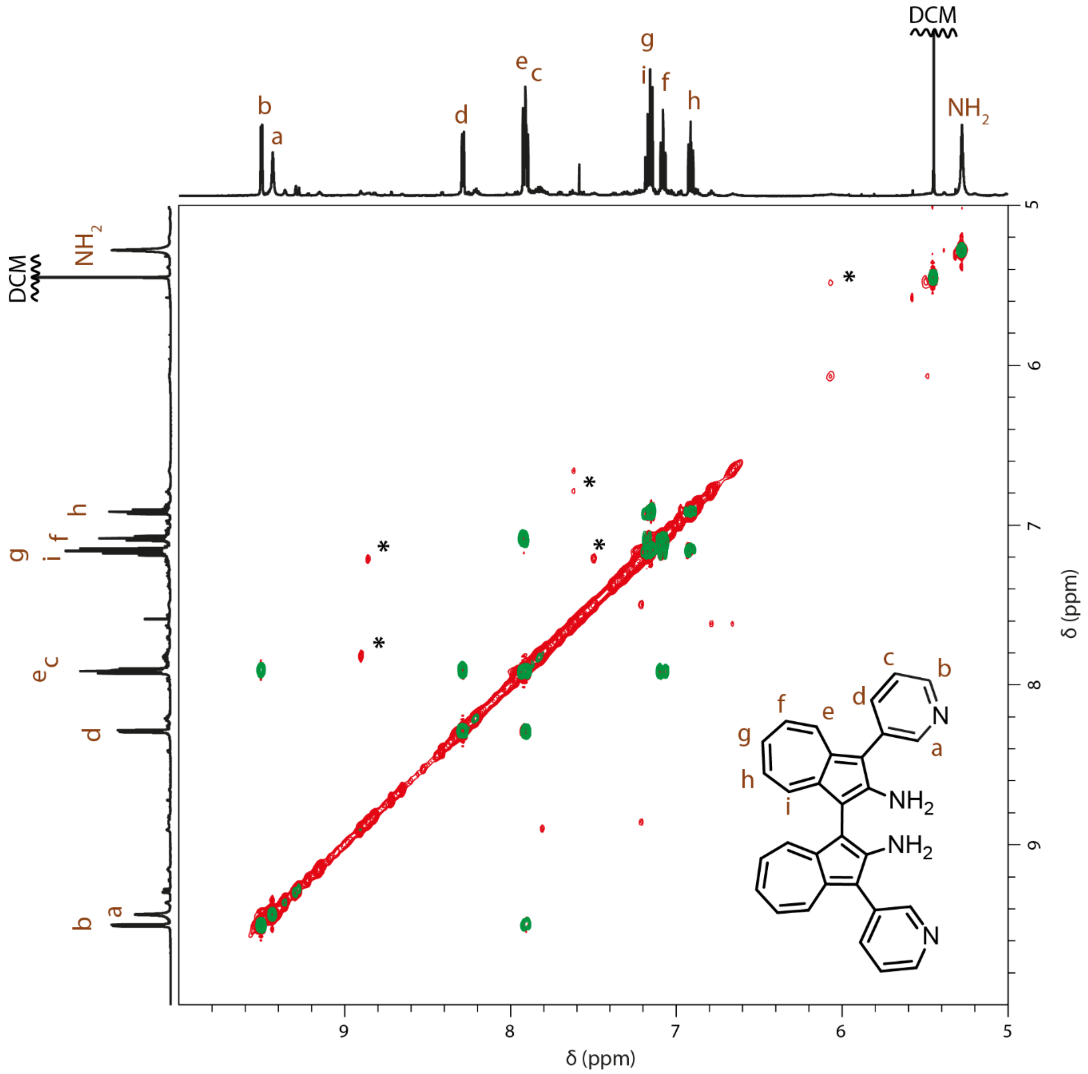

Figure S25: ^{1}H-^{1}H COSY (green traces) and NOESY (red traces) NMR (600 MHz, 298 K, CD_3CN) spectra of [Pd_2**BAAZU-1**$^{rac}{}_4$]$(BF_4)_4$. NOE correlation belonging to trace species are highlighted with an asterisk.

3.1.1. Evolution of NMR signals sets after addition of Pd(II) to the racemic ligand over time

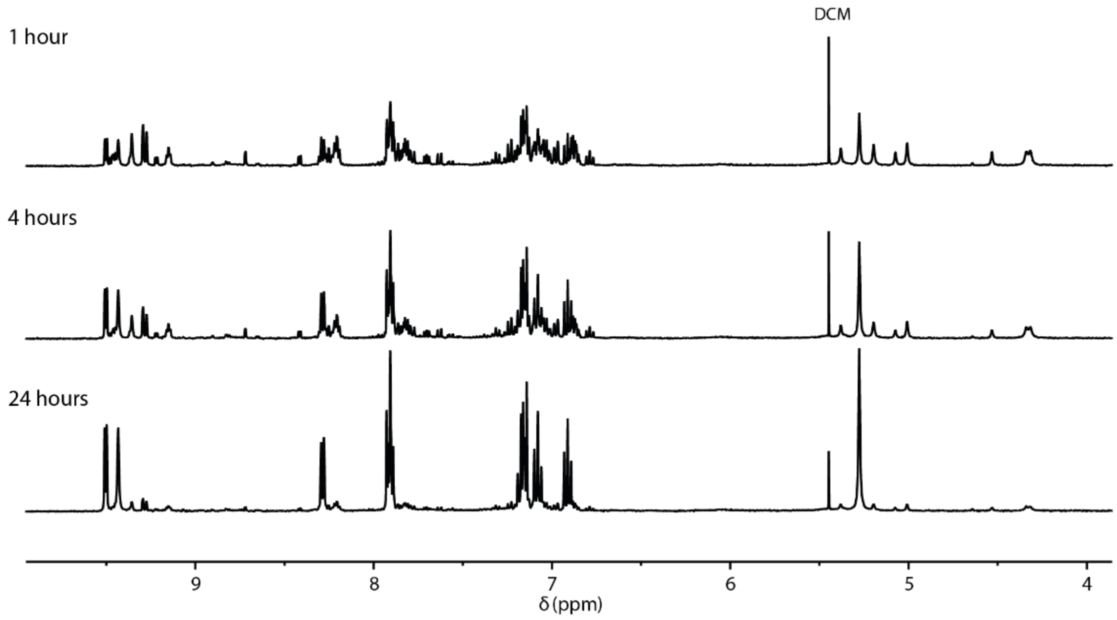

Figure S27: ^{1}H-NMR (500 MHz, CD_3CN, 298 K) of formation of cage [Pd$_2$**BAAZU-1**^{rac}$_4$](BF_4)$_4$ over time while heating at 70°C.

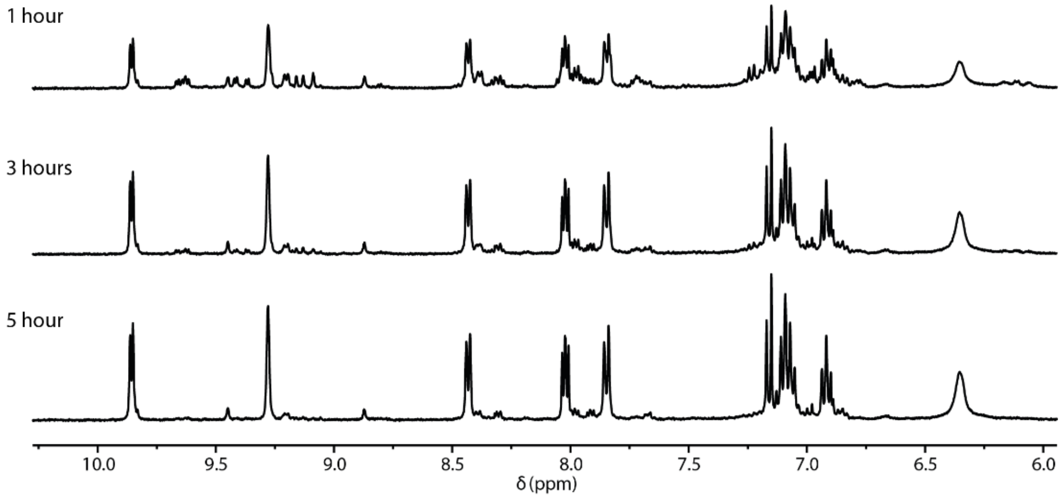

Figure S28: ^{1}H-NMR (500 MHz, DMSO-d_6, 298 K) of formation of cage [Pd$_2$**BAAZU-1**^{rac}$_4$](BF_4)$_4$ over time while heating at 70°C.

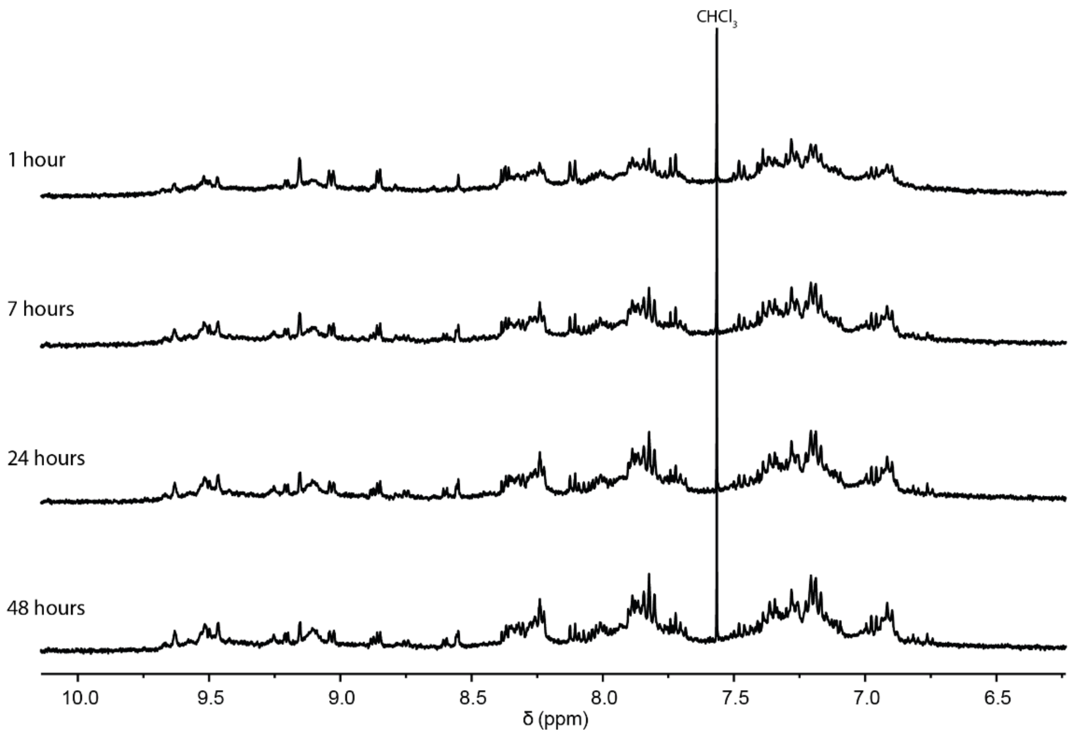

Figure S29: ^{1}H-NMR (500 MHz, CD_3NO_2, 298 K) of formation of cage [Pd$_2$**BAAZU-1**$^{rac}{}_4$]$(BF_4)_4$ over time while heating at 70°C.

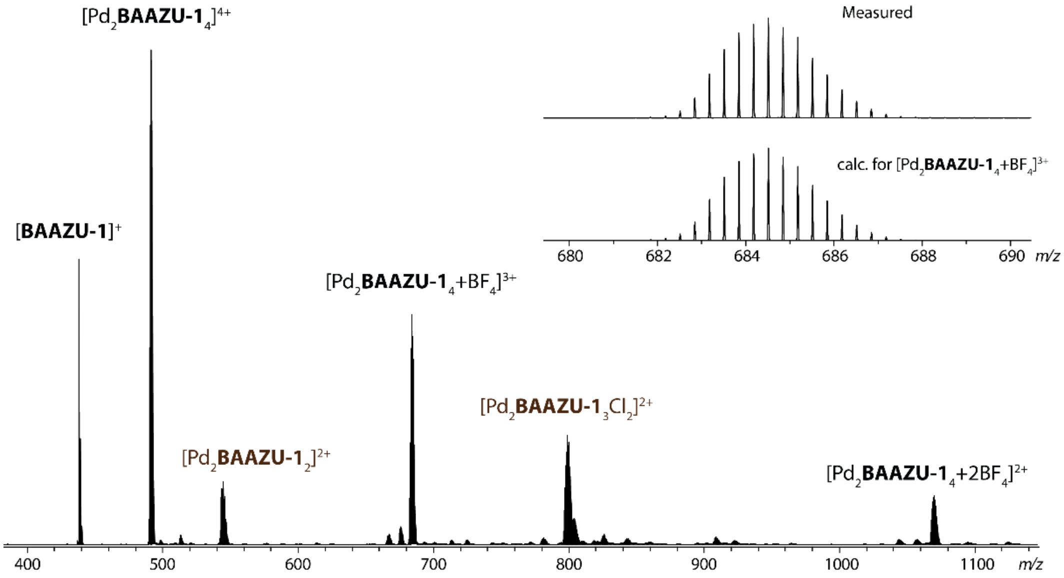

Figure S30: ESI-MS spectrum of cage [Pd$_2$**BAAZU-1**$^{rac}{}_4$]$(BF_4)_4$ after 1 h in CD_3CN, at 70°C.

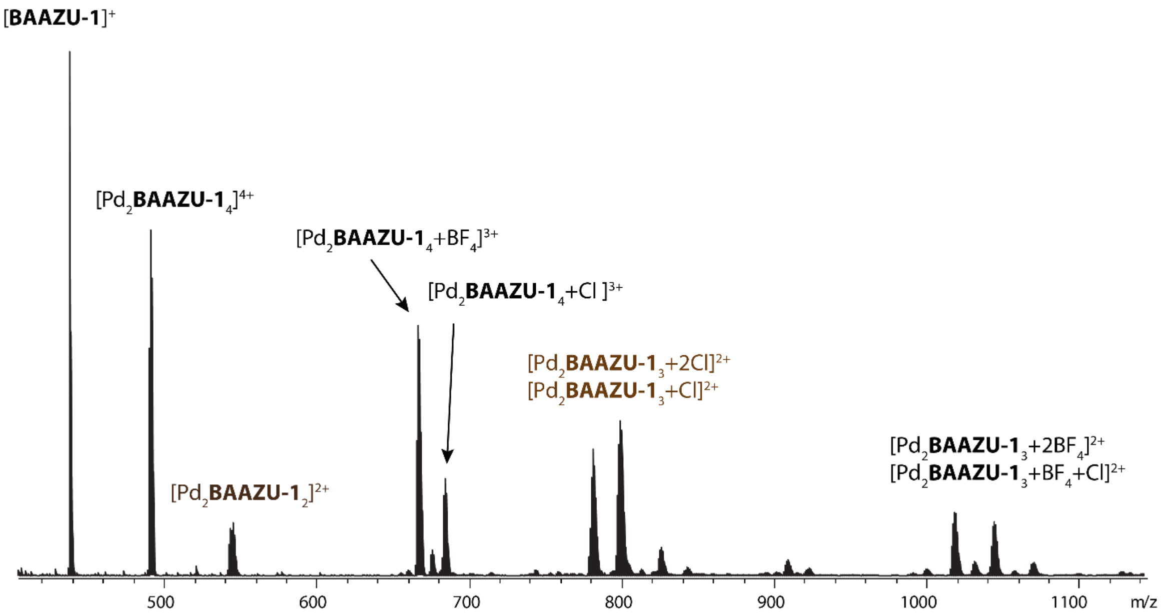

Figure S31: ESI-MS spectrum of cage [Pd_2**BAAZU-1**$^{rac}{}_4$]$(BF_4)_4$ after 24 h in CD_3CN, at 70°C. The chloride adducts are probably formed because of a contamination in the spectrometer.

3.1.2. Titration of Pd_2**BAAZU-1**$^{rac}{}_4$ in CD_3NO_2 with DMSO-d_6

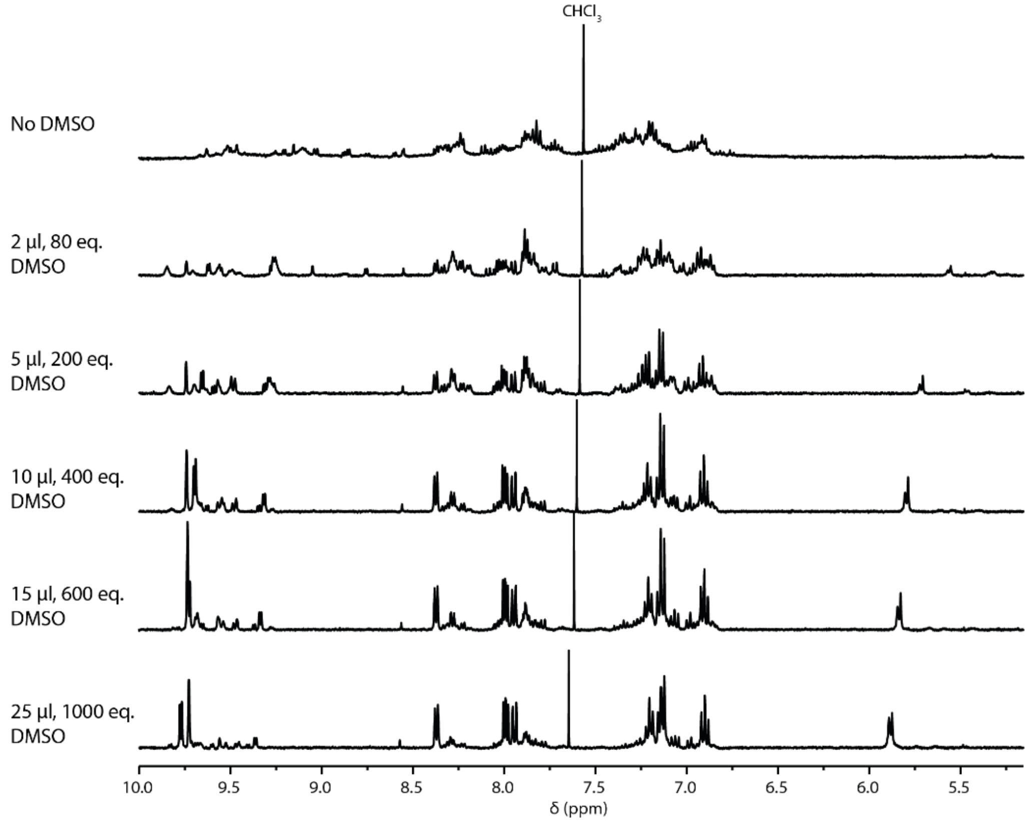

Figure S32: ^{1}H-NMR (500 MHz, CD_3NO_2, 298 K) of [Pd_2**BAAZU-1**$^{rac}{}_4$]$(BF_4)_4$ (1.4 mM in ligand equivalent) titrated with DMSO-d_6. The DMSO amount is expressed as equivalents to the cage.

3.1.3. Symmetry of the four diastereomers of the cage

In the homochiral cage, there exist five rotation axes, leading to every half of every ligand to be symmetrically equivalent to every other one; only one set of signals is therefore observed (similar to the free ligand). In the *RRRS*/*SSSR* isomer, one C_2 axis can be found. This low symmetry would lead to an NMR spectrum with four sets of signals (not observed). In the *meso-cis* cage, the additional horizonal mirror plane, cutting the cage through the two palladium centres and in-between the ligands, reduces the number of signals to two sets (not observed). Finally, in the *meso-trans* cage, the three C_2 axes and the two vertical dihedral mirror planes lead to a single set of signals.

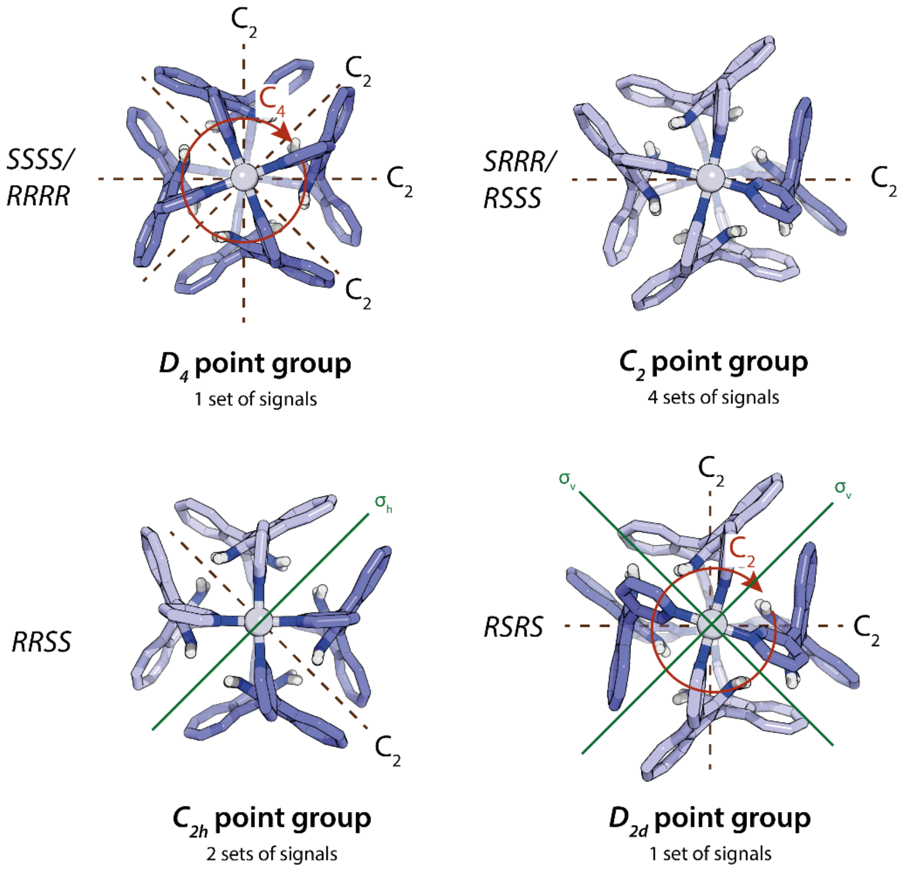

Figure S33: Symmetry operations in the four possible diastereomers of the Pd_2**BAAZU-1**$_4$ cage. The observed NMR pattern (showing only one set of signals) of the cage formed from the racemic ligand would only be explainable by either narcissistic self-sorting to a 1 : 1 mixture of isomers *RRRR* and *SSSS* or by formation of the D_{2d}-symmetric *RSRS meso-trans* cage. As cage formation from the enantiomerically pure ligand reveals an NMR signal set of pronouncedly different chemical shifting as observed for the cage formed from the racemic ligand, the latter could thus be confirmed to form only D_{2d}-symmetric *RSRS meso-trans* isomer under social chiral self-sorting in solution. This was further confirmed by the X-ray structure obtained from this solution and by computations considering explicit solvents and counter anions (see below).

3.2. Pd_2**BAAZU-1**$^{enant}{}_4$ from enantiomerically pure ligand

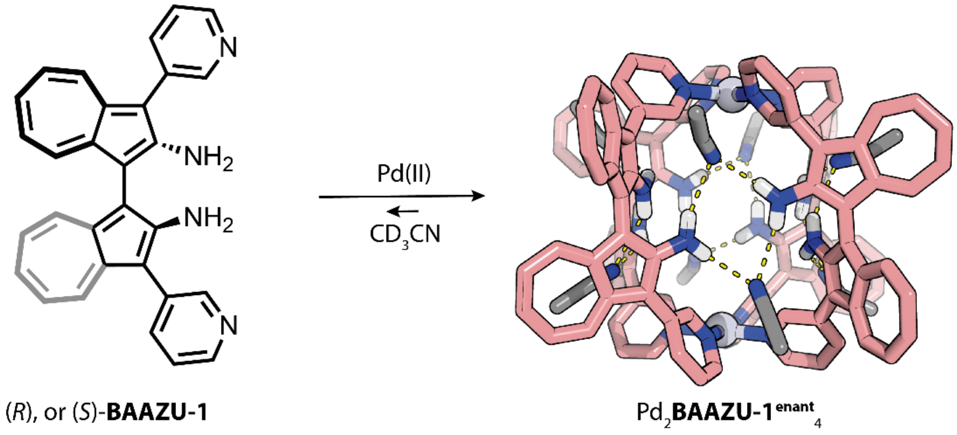

Scheme S14: Formation of Pd_2**BAAZU-1**$^{enant}{}_4$ from the enantiopure ligands (*R*)- or (*S*)-**BAAZU-1**.

The coordination cage [Pd_2**BAAZU-1**$^{enant}{}_4$]$(BF_4)_4$ was synthesised as a 0.7mM solution in CD_3CN according to the general procedure, using [$Pd(CH_3CN)_4$]$(BF_4)_4$ as the palladium(II) salt and enantiopure (*R*)- or (*S*)-**BAAZU-1**.

^{1}H-NMR (600 MHz, CD_3CN) δ 8.75 (s, 2H), 8.44 (dd, *J* = 5.9, 1.3 Hz, 2H), 8.33 (dd, *J* = 7.9, 1.7 Hz, 2H), 7.90 (d, *J* = 10.0 Hz, 2H), 7.73 (dd, *J* = 7.9, 5.9 Hz, 2H), 7.66 (d, *J* = 10.0 Hz, 2H), 7.34 (t, *J* = 9.5 Hz, 2H), 7.20 (t, *J* = 10.0 Hz, 2H), 7.10 (t, *J* = 9.6 Hz, 2H), 5.41 (s, 4H).

Due to the very long acquisition needed for ^{13}C-NMR and concerns over partial epimerisation of the compound over this period, the ^{13}C-NMR spectrum was not measured in this case.

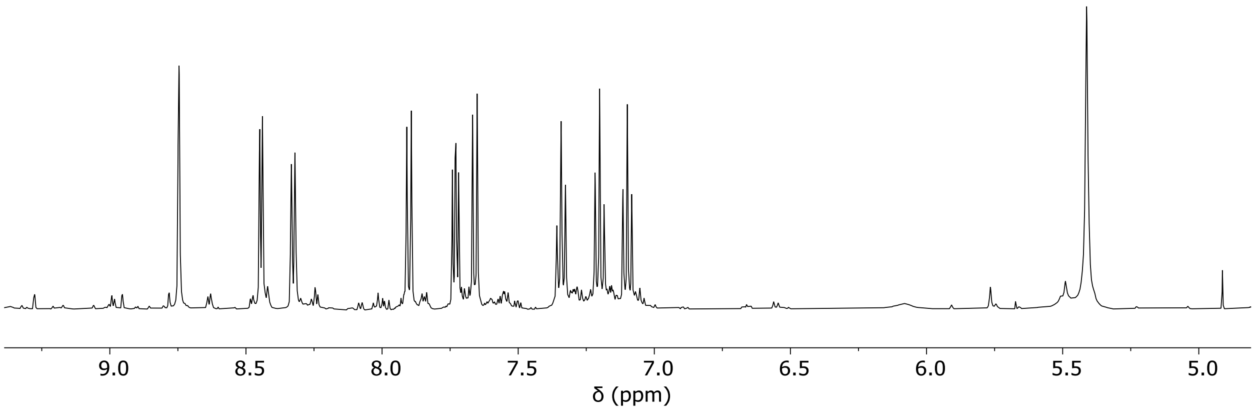

Figure S34: ^{1}H-NMR (600 MHz, 298 K, CD_3CN) spectrum of [Pd_2**BAAZU-1**$^{enant}{}_4$]$(BF_4)_4$.

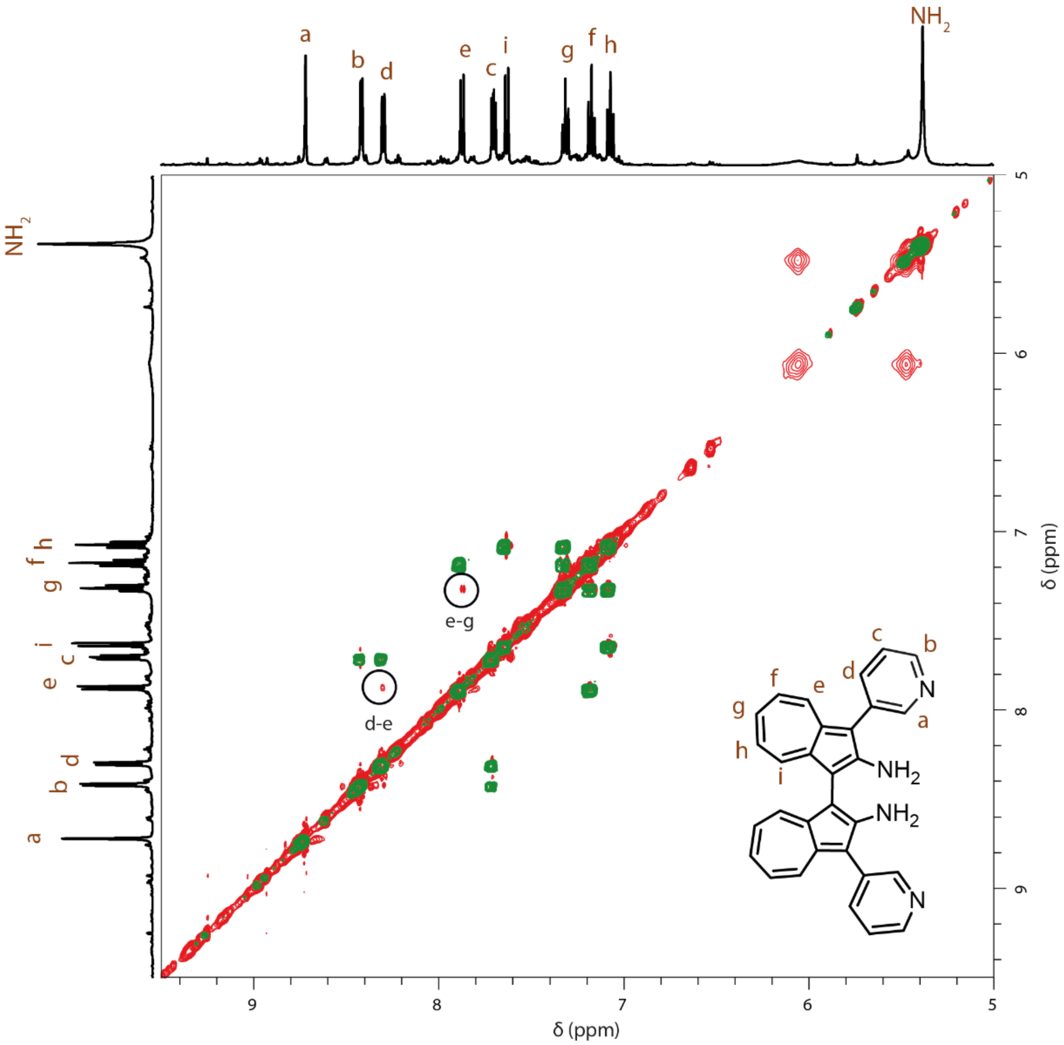

Figure S35: ^{1}H-^{1}H COSY (green traces) and NOESY (red traces) NMR (600 MHz, 298 K, CD_3CN) spectra of [Pd_2**BAAZU-1**$^{enant}_4$]$(BF_4)_4$.

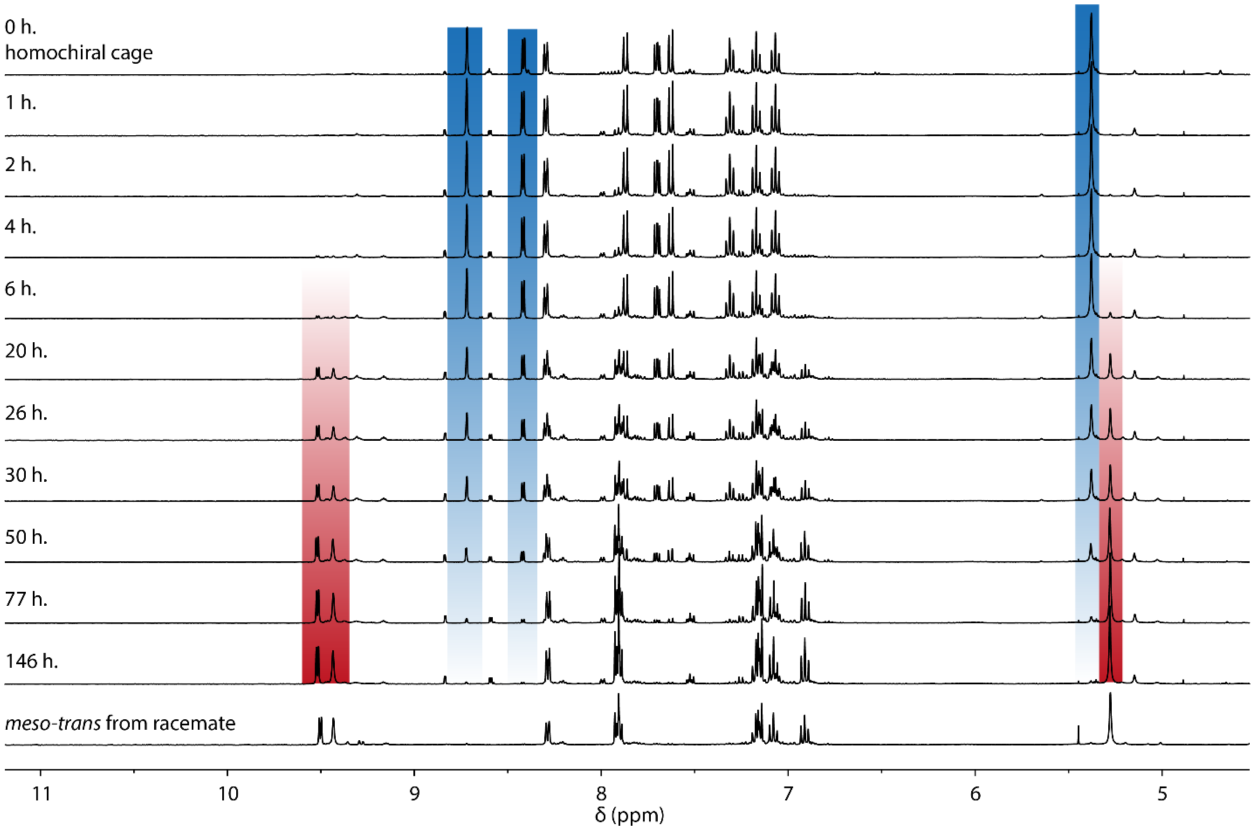

Fig. S36: ^{1}H-NMR (500 MHz, 298 K, CD_3CN) spectra of [Pd_2**BAAZU-1**$^{enant}{}_4$]$(BF_4)_4$ over time in acetonitrile, heating at 70°C. The system fully converts over time into the *meso-trans* isomer. However, it takes much longer than what was observed by CD spectroscopy (approx. 5 h.). This discrepancy may be explained by the difference in concentration. Indeed, the present concentration is 2.8 mM in ligand, while the kinetics of racemisation by CD were measured at 0.125 mM. The larger concentration in the NMR experiment may thus push the equilibrium in favour of the inclusion of the BF_4^- anion inside of the cavity. In turn, the presence of the encapsulated counteranion may block (or at least slow down) the racemisation process of the ligands. On the contrary, the low concentration in cage in the CD experiment favours less the encapsulation of BF_4^- by the cage, and by consequent, the racemisation of the ligands is less hindered and therefore faster.

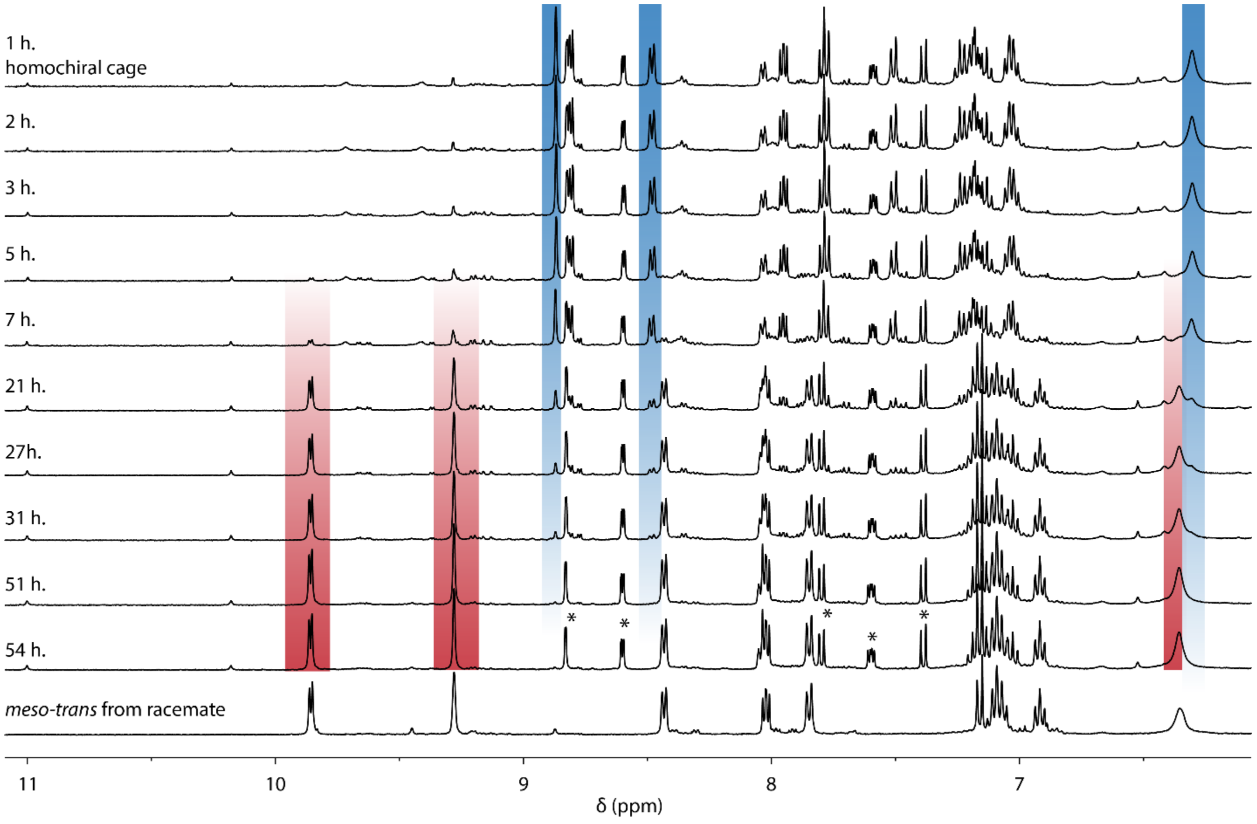

Fig. S37: ^{1}H-NMR (500 MHz, 298 K, CD_3CN) spectra of [Pd_2**BAAZU-1**$^{enant}{}_4$]$(BF_4)_4$ over time in DMSO, heating at 70°C. The system fully converts over time into the *meso-trans* isomer. * = free ligand

3.3. Pd_2**BAAZU-2**$^{rac}{}_4$ from racemic ligand

The coordination cage [Pd_2**BAAZU-2**$^{rac}{}_4$]$(BF_4)_4$ (mixture of all isomers) was synthesised as a 0.7 mM solution in CD_3CN, DMSO-d_6, or CD_3NO_2 according to the general procedure, using [$Pd(CH_3CN)_4$]$(BF_4)_4$ as the palladium (II) salt and the racemate of **BAAZU-2**. A mixture of isomers was obtained in every tested solvent.

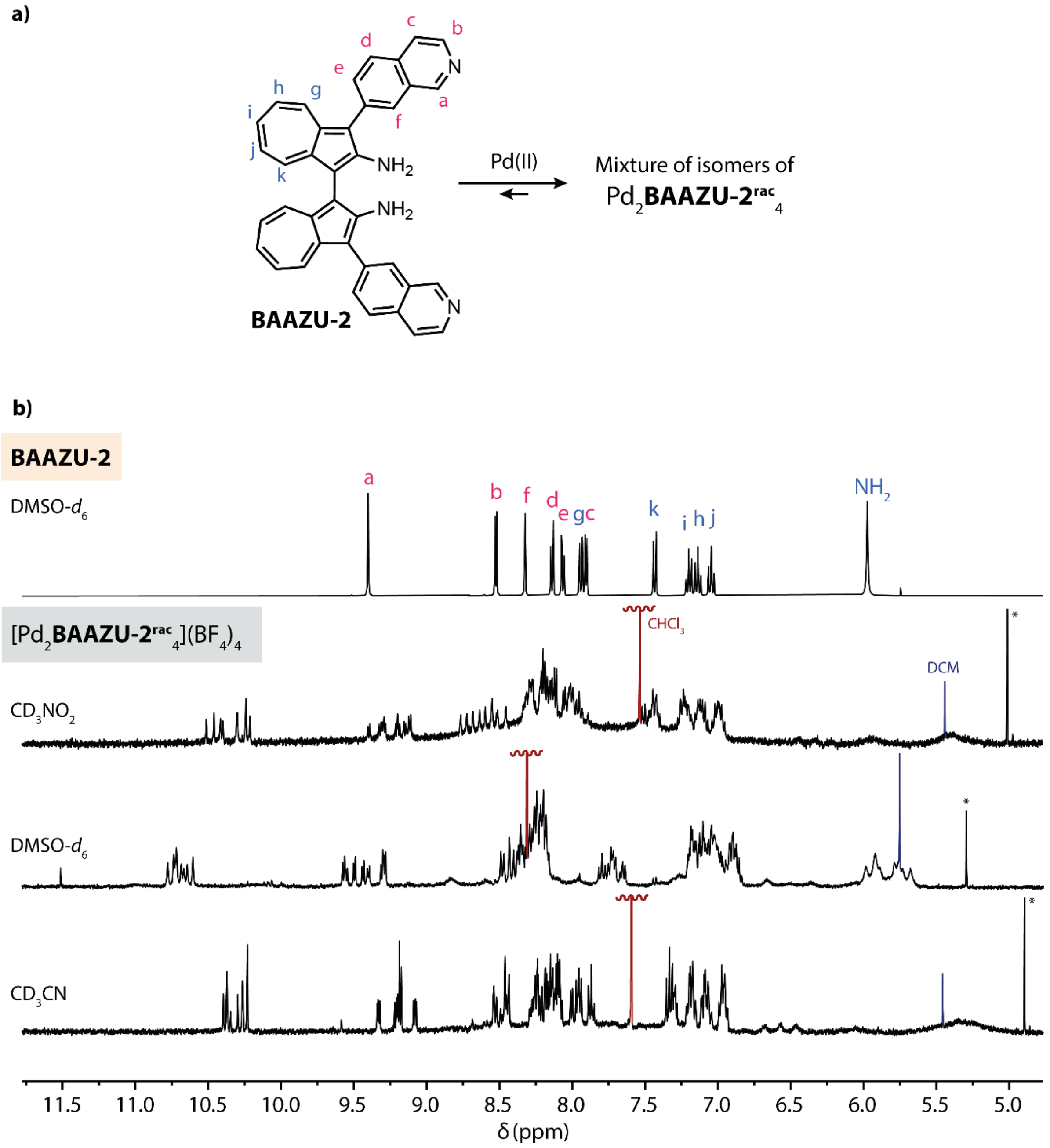

Figure S38: a) Scheme of the synthesis of the mixture of the isomers of [Pd_2**BAAZU-2**$^{rac}{}_4$]$(BF_4)_4$. b) ^{1}H-NMR (500 MHz, 298 K) of **BAAZU-2**, and of the [Pd_2**BAAZU-2**$^{rac}{}_4$]$(BF_4)_4$ mixtures in CD_3NO_2, DMSO-d$_6$, and CD_3CN.

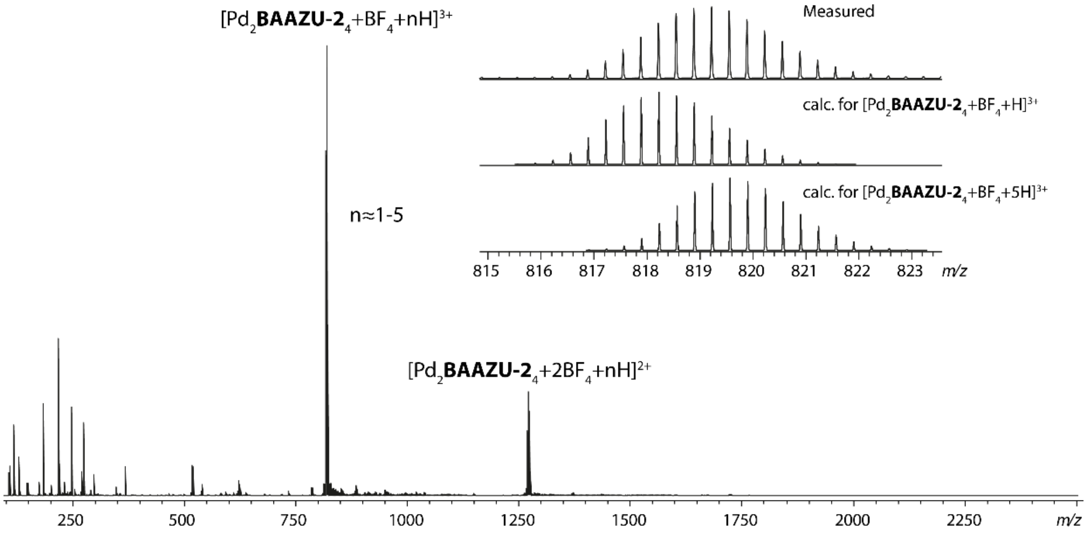

Figure S39: ESI-MS spectrum of cage [Pd_2**BAAZU-2**$^{rac}{}_4$]$(BF_4)_4$ prepared in CD_3NO_2.

3.4. Pd_2BAAZU-2$^{enant}{}_4$ from enantiomerically pure ligand

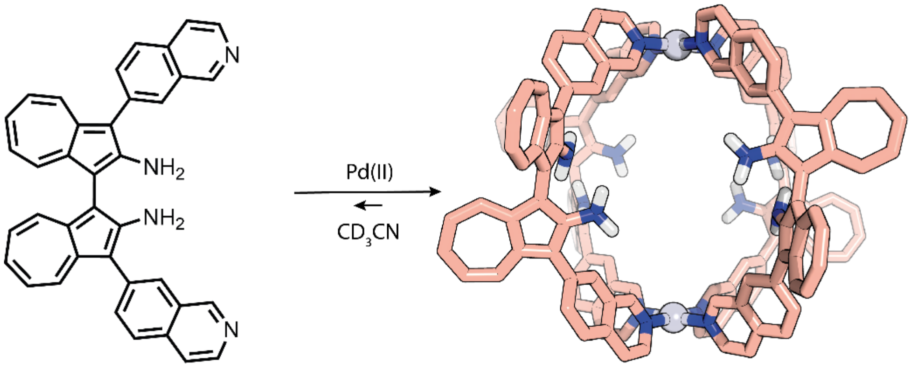

(R)- or (S)-**BAAZU-2** $\qquad$ Pd_2**BAAZU-2**$^{enant}{}_4$

Scheme S15: Formation of Pd_2**BAAZU-2**$^{enant}{}_4$ from the enantiopure ligand (R)- or (S)-**BAAZU-2**.

The coordination cage Pd_2**BAAZU-2**$^{enant}{}_4$ was synthesised from the enantiopure ligand **BAAZU-2** with either BF_4^- or $BArF_{20}^-$ counteranions from the corresponding Pd(II) salt in CD_3CN as a 0.7 mM solution. The two solutions were mixed and stirred at 70°C for 5 min. The following shifts are from the $BArF_{20}^-$ species.

^{1}H-NMR (600 MHz, CD_3CN) δ 10.08 (s, 2H), 9.18 (d, J = 6.7 Hz, 2H), 8.42 – 8.39 (m, 2H), 8.26 (dd, J = 8.3, 1.7 Hz, 2H), 8.15 (d, J = 8.3 Hz, 2H), 8.11 (d, J = 6.8 Hz, 2H), 8.00 – 7.95 (m, 2H), 7.35 (dd, J = 10.2, 1.0 Hz, 2H), 7.23 – 7.17 (m, 2H), 7.10 (t, J = 9.8 Hz, 2H), 6.98 (ddd, J = 10.0, 9.0, 0.9 Hz, 2H), 5.29 (s, 4H).

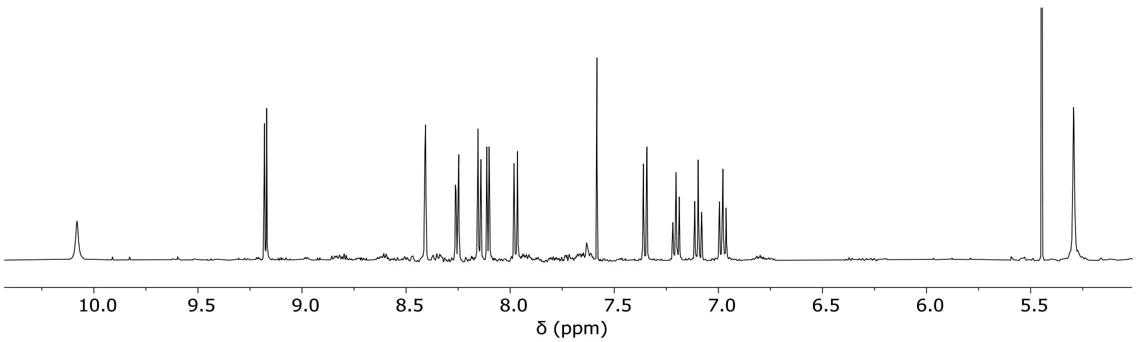

Figure S40: ^{1}H-NMR (600 MHz, 298 K, CD_3CN) spectrum of [Pd_2**BAAZU-1**$^{enant}{}_4$]($BArF_{20}$)$_4$.

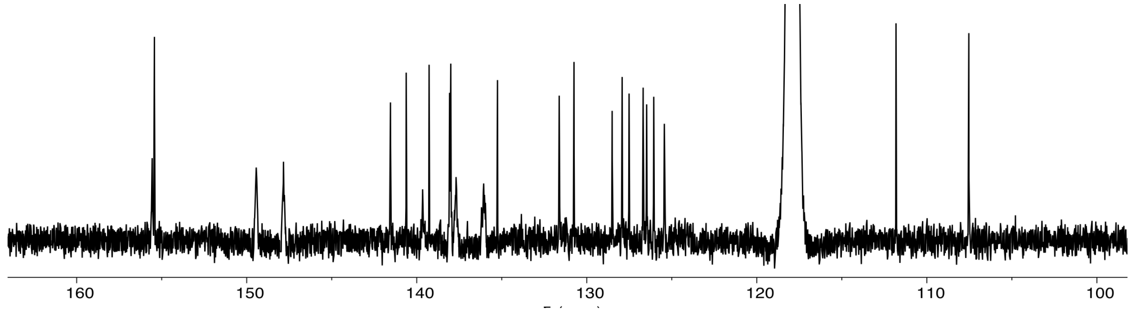

Figure S41: ^{13}C-NMR (151 MHz, 298 K, CD_3CN) spectrum of [Pd_2**BAAZU-2**$^{enant}{}_4$]($BArF_{20}$)$_4$.

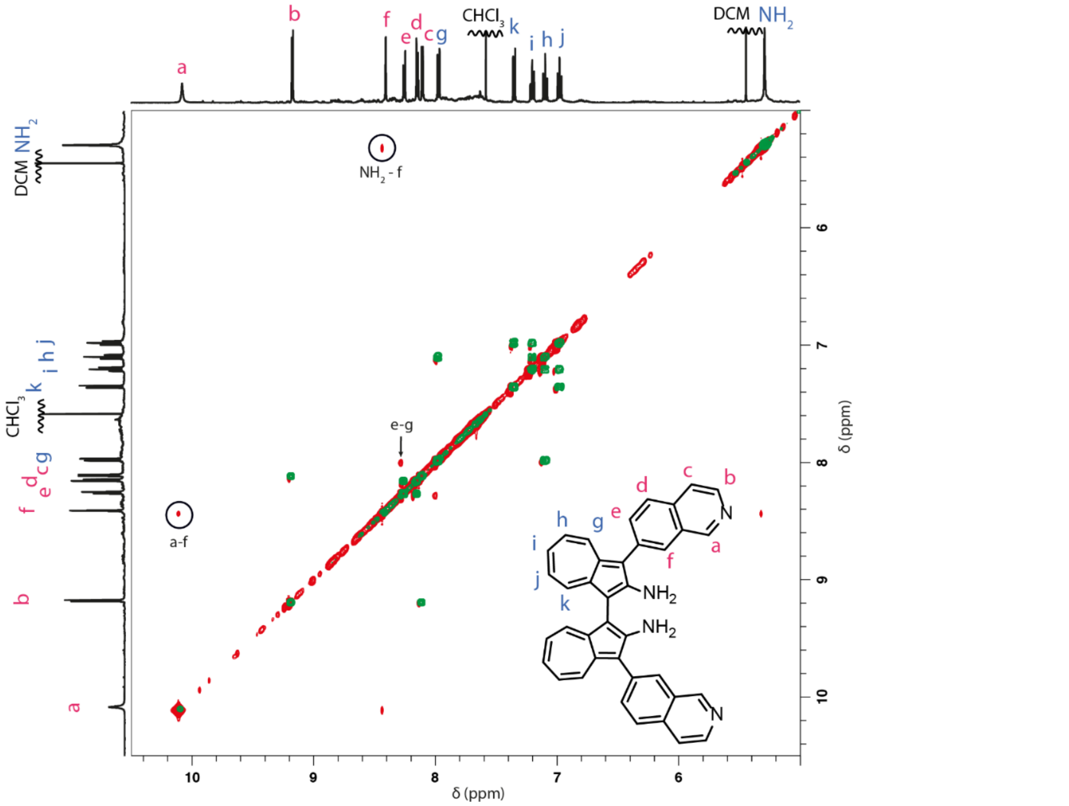

Figure S42: ^{1}H-^{1}H COSY (green traces) and NOESY (red traces) NMR (600 MHz, 298 K, CD_3CN) spectra of [Pd_2**BAAZU-2**$^{enant}{}_4$]($BArF_{20}$)$_4$. Important NOE correlations are highlighted.

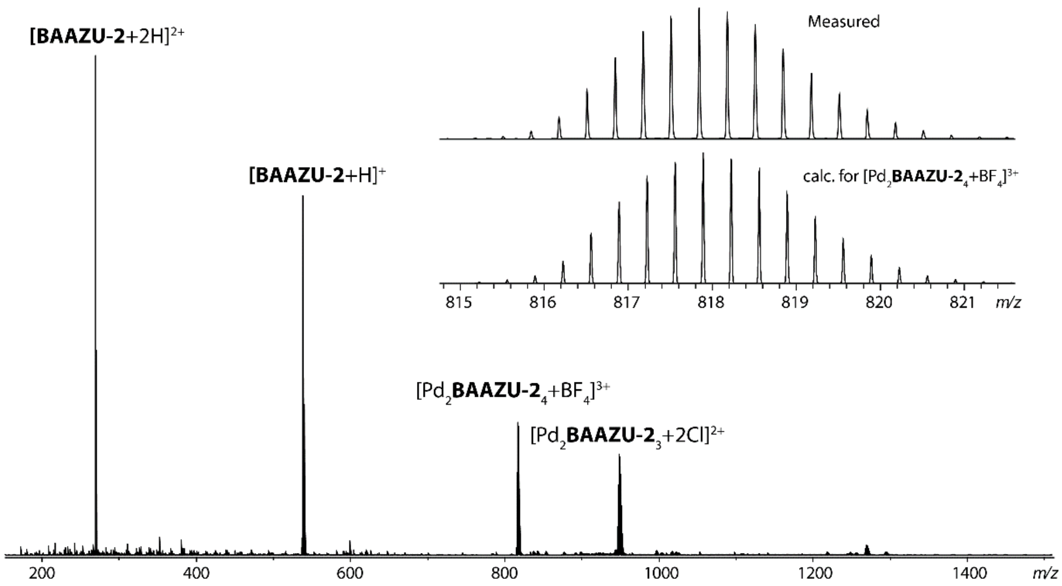

Figure S43: ESI-MS spectrum of cage [Pd_2**BAAZU-2**$^{enant}_4$]$(BF_4)_4$.

4. Circular dichroism spectra

Cuvette path length: 2mm (except when noted). Concentration in ligand: 1.25 mM or 0.125 mM.

4.1. CD of Intermediate 8

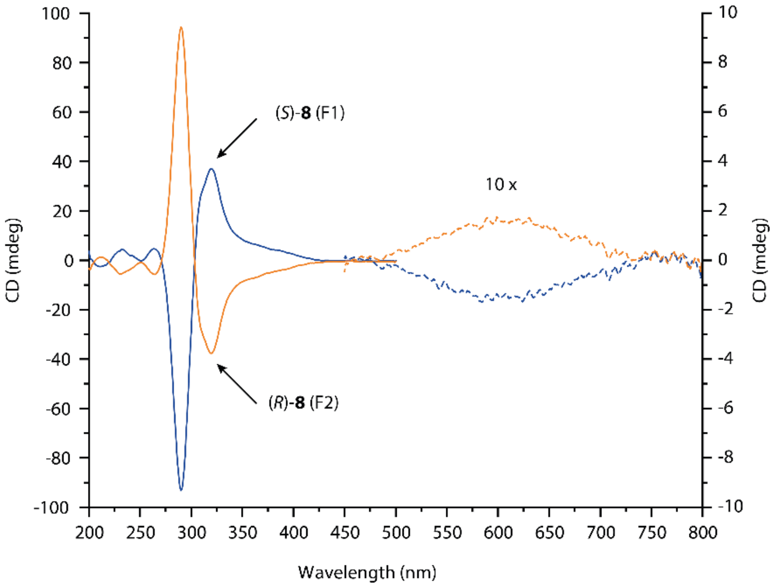

Figure S44: Circular dichroism of the two fractions of the intermediate **8**. The 450-800 nm spectra of the concentrated solutions are plotted on the right y-axis.

4.2. CD of BAAZU-1

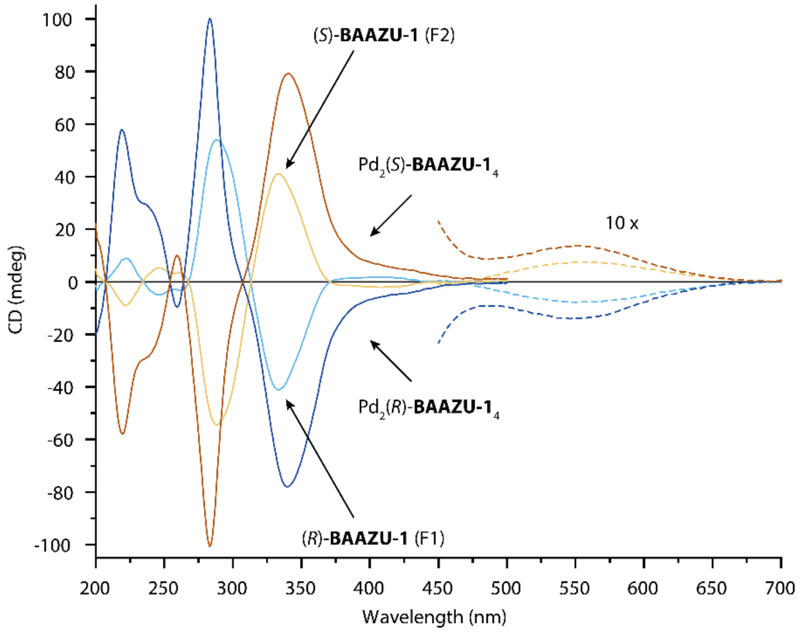

Figure S45: Circular dichroism of the two fractions of the **BAAZU-1** ligand and of the corresponding [Pd_2**BAAZU-1**$^{enant}{}_4$]$(BF_4)_4$ in acetonitrile (0.125 mM: continuous lines, 1.25 mM: dashed lines). Due to its low solubility, the higher concentration data for pure ligand **BAAZU-1** was recorded at 0.25 mM with a cuvette with a 1 cm path length.

4.3. CD of BAAZU-2

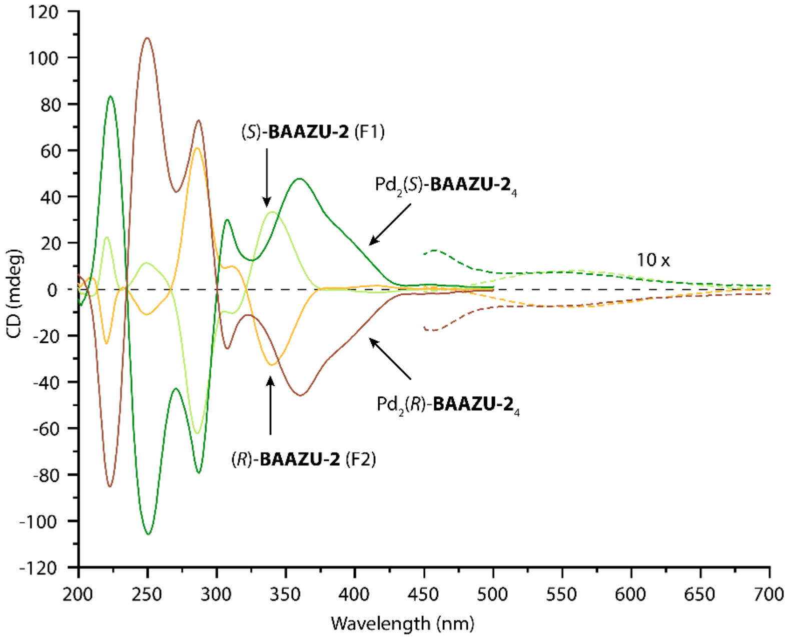

Figure S46: Circular dichroism of the two fractions of the **BAAZU-2** ligand and of the corresponding [Pd_2**BAAZU-2**$^{enant}{}_4$]$(BF_4)_4$ in acetonitrile (0.125 mM: continuous lines, 1.25 mM: dashed lines).

4.4. Racemisation kinetics of BAAZU-1

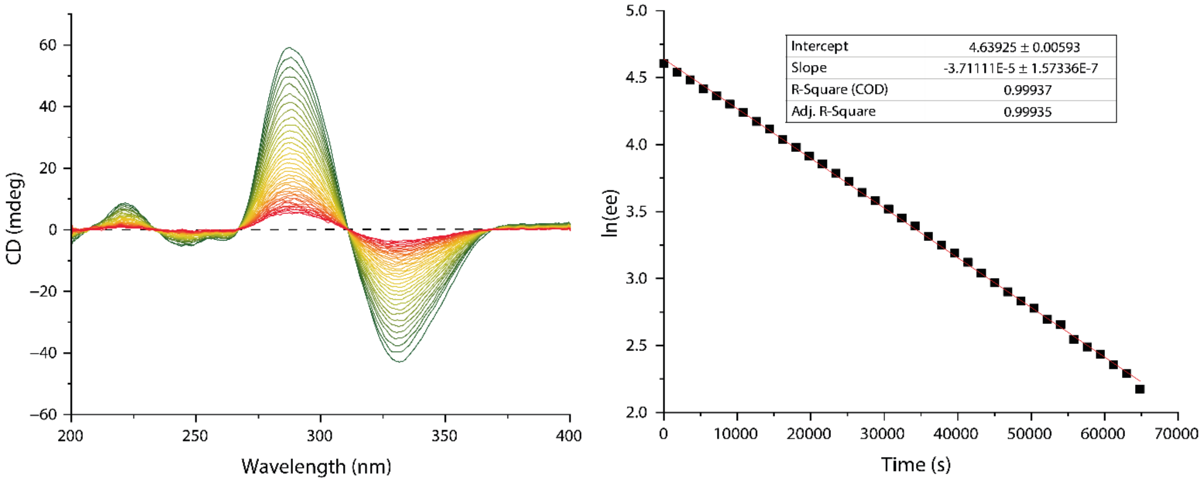

Figure S47: (left) CD spectra of (*R*)-**BAAZU-1** in acetonitrile at 70°C. Each curve is measured with a 30 min. interval over 20.5 h. (right) natural logarithm of the enantiomeric excess (*ee*) over time. Red line: linear fit of the data.

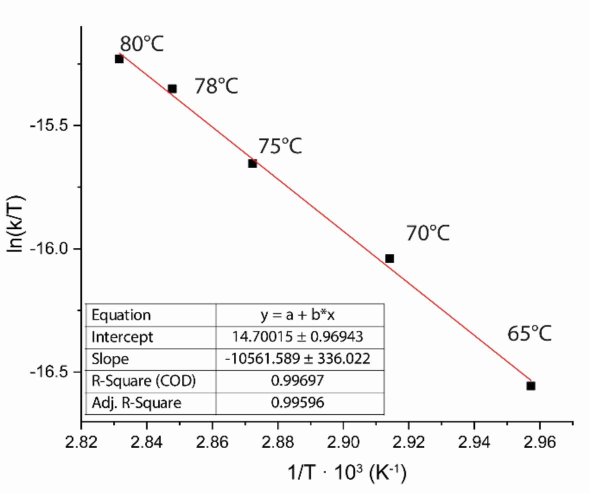

Figure S48: Eyring plot of the racemisation of **BAAZU-1** in acetonitrile.

The calculated kinetic parameters for the racemisation of **BAAZU-1** in acetonitrile are:

$$\Delta H^{\ddagger} = 87809\, J/mol$$

$$\Delta S^{\ddagger} = -75.3\, J/mol$$

$$\Delta G^{\ddagger}(RT) = 110.3\, kJ/mol = 26.4\, kcal/mol$$

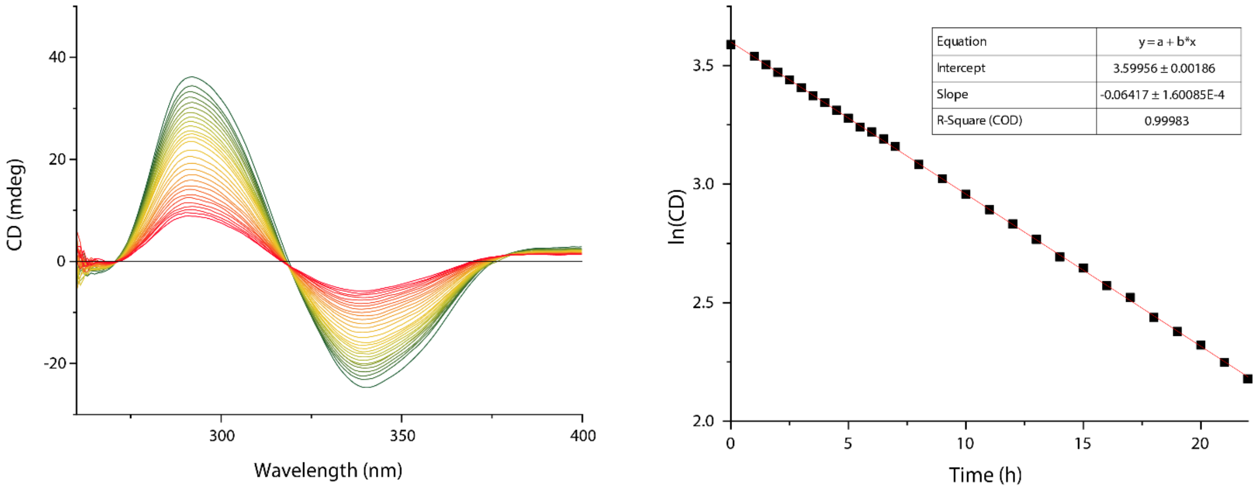

Fig. S49: (left) CD spectra of (*R)*-**BAAZU-1** in DMSO at 70°C. Each curve is measured with a 30 or 60 min. interval over 22 h. (right) natural logarithm of the CD signal over time. Red line: linear fit of the data.

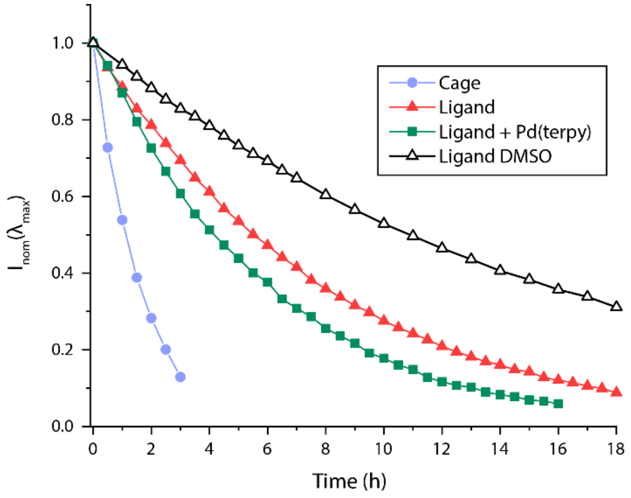

Fig. S50: Decay over time of the CD signal of **BAAZU-1** and of its two coordination species in MeCN, as well as in DMSO, at 70 °C measured at their λ_{max}.

4.5. Racemisation kinetics of $[Pd_2BAAZU\text{-}1(terpy)_2](BF_4)_4$

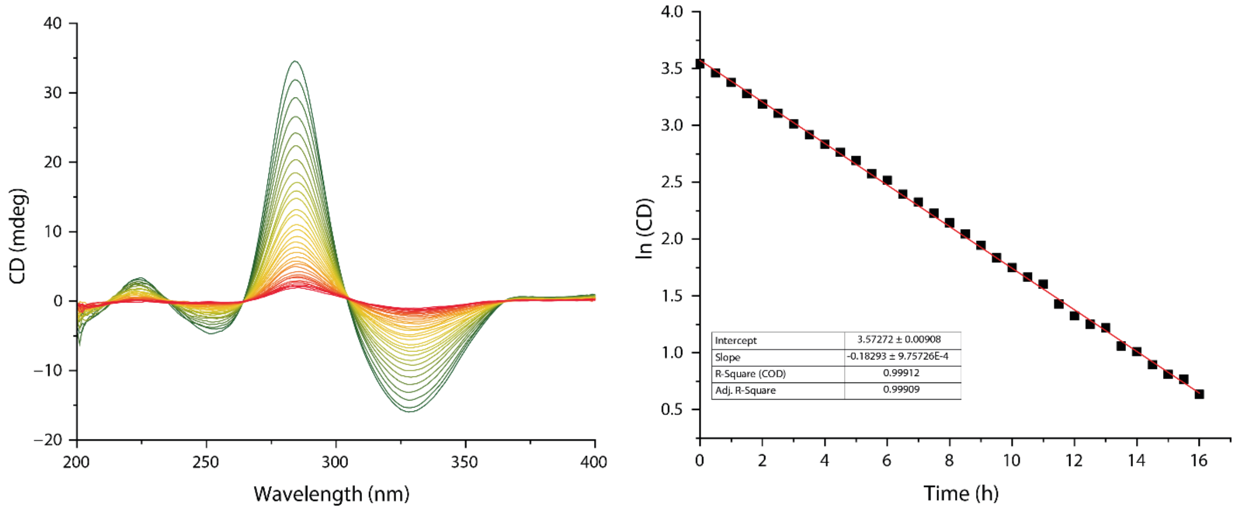

Figure S51: (left) CD spectra of $[Pd_2(R)$-**BAAZU-1**$(terpy)_2](BF_4)_4$ in acetonitrile at 70°C (0.125 mM, 1 mm cuvette). Each curve is measured with a 30 min interval over 13 h. (right) natural logarithm of the CD over time. Red line: linear fit of the data.

4.6. Racemisation kinetics of $[Pd_2BAAZU\text{-}1^{enant}{}_4](BF_4)_4$

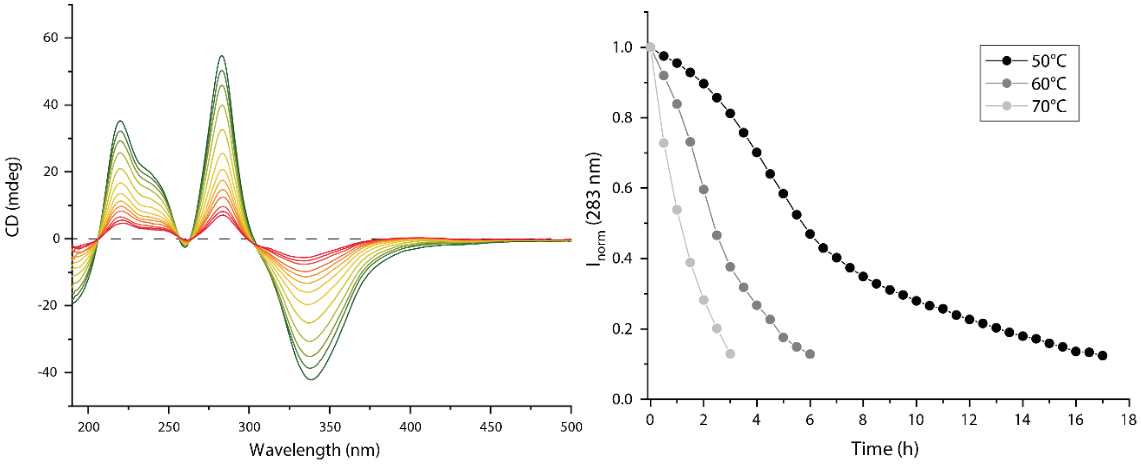

Figure S52: (left) CD spectra of $[Pd_2(R)$-**BAAZU-1**$_4](BF_4)_4$ in acetonitrile at 60°C. Each curve is measured with a 30 min interval over 6 h. (right) plot of the normalised intensity of the band at 283 nm at 50°C, 60°C, and 70°C. Note the sigmoidal shape of the curves (here: linear plot, not subjected to logarithm).

4.7. Racemisation of [Pd_2BAAZU-2^{enant}$_4$](BF_4)$_4$

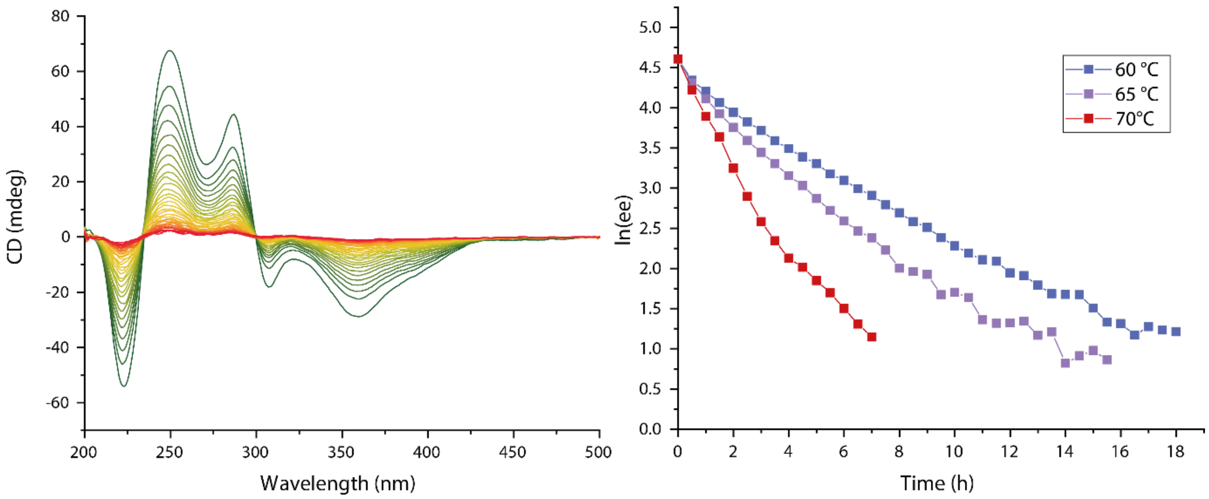

Figure S53: (left) CD spectra of [Pd_2(*R)*-**BAAZU-2**$_4$](BF_4)$_4$ in acetonitrile at 60°C. Each curve is measured with a 30 min interval over 6 h. (right) plot of the normalised intensity of the band at 283 nm at 60°C, 65°C, and 70°C.

5. Host-guest experiments

Guest titration experiments were performed with [Pd_2**BAAZU-2**^{enant}$_4$]($BArF_{20}$)$_4$ in CD_3CN solution. Association constants were calculated with BindFit (http://app.supramolecular.org/bindfit/). The effective concentration of the cage in the sample was compared by integration of the signals of the cage and of the tetrabutylammonium counterions of the guests. All titrations were done using aliquots of the same batch of cage solution.

5.1. $Cr^0(CO)_6$

The titration was recorded by ^{1}H-NMR, with a cage concentration of 0.16 mM. Calculated association constant: 111.4 M^{-1} ± 4.6 %. Due to the low binding affinity, the host-guest complex could not be detected by ESI-MS. Due to the absence of the signals from counterions for this uncharged guest, the effective concentration of the cage was determined from the calculated values of the next experiments, as they were measured from the same batch of cage solution.

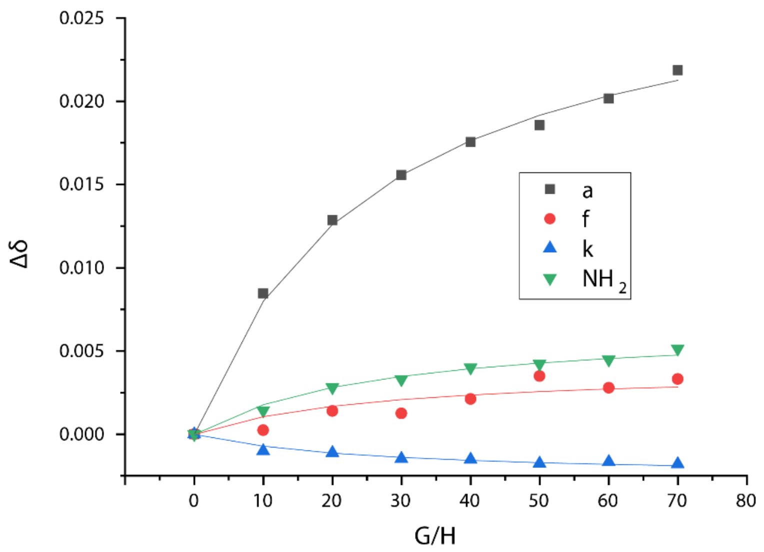

Figure S54: ^{1}H-NMR peak shifts upon addition of $Cr(CO)_6$ to $[Pd_2$**BAAZU-2**$^{enant}{}_4](BArF_{20})_4$ in CD_3CN. Solid lines: fitting curves.

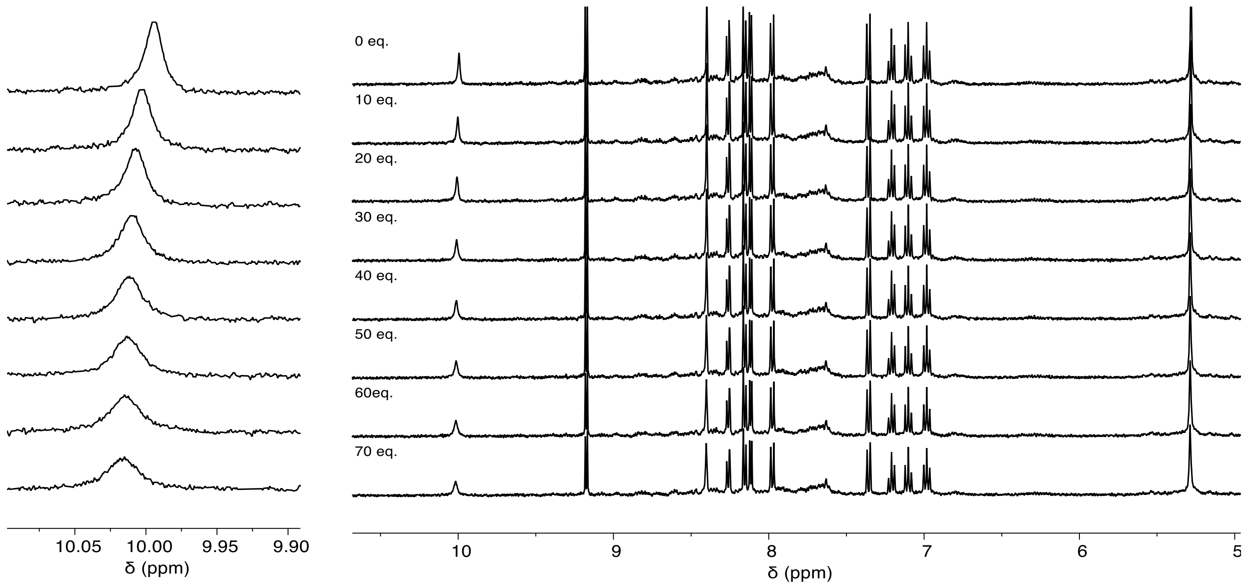

Figure S55: ^{1}H-NMR (500 MHz, 298 K, CD_3CN) titration of $[Pd_2$**BAAZU-2**$^{enant}{}_4](BArF_{20})_4$ upon addition of $Cr(CO)_6$. The region of the **a** proton is zoomed in in the insert on the left.

5.2. $(NBu_4)_2[Pt^{IV}(CN)_6]$

The titration was recorded by ^{1}H-NMR, UV/Vis and CD. However, precipitation was directly observed after the addition of more than 1.2 eq. of the guest, preventing us from calculating an accurate binding constant. The rough value calculated from the NMR data is: 86681 M^{-1} ± 47%.

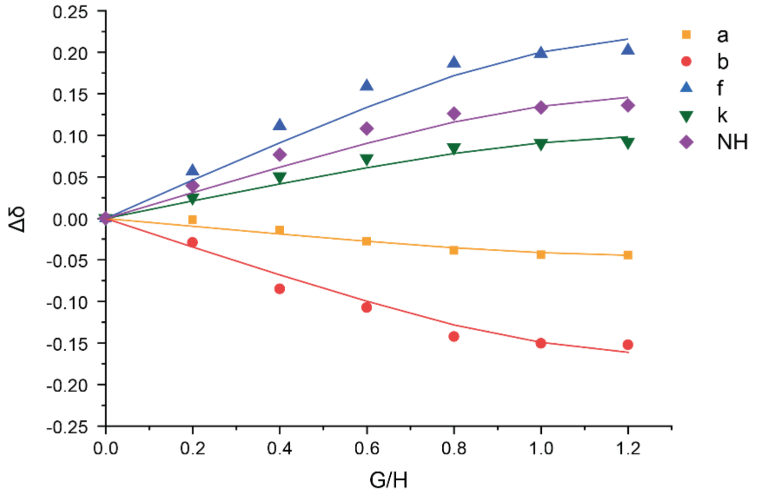

Figure S56: ^{1}H-NMR peak shifts upon addition of $(NBu_4)_2[Pt(CN)_6]$ to [Pd_2**BAAZU-2**$^{enant}{}_4$]$(BArF_{20})_4$ in CD_3CN. Solid lines: fitting curves.

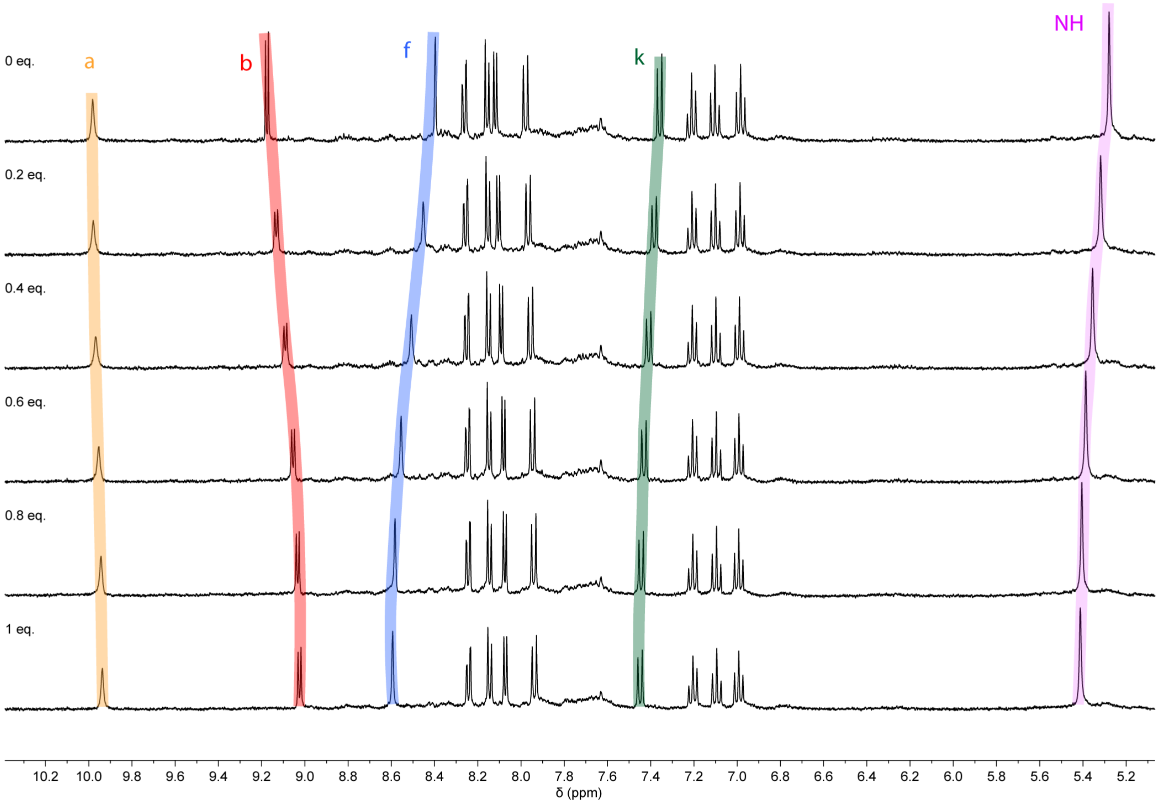

Figure S57: ^{1}H-NMR (500 MHz, 298 K, CD_3CN) titration of [Pd_2**BAAZU-2**$^{enant}{}_4$]$(BArF_{20})_4$ upon addition of $(NBu_4)_2[Pt(CN)_6]$.

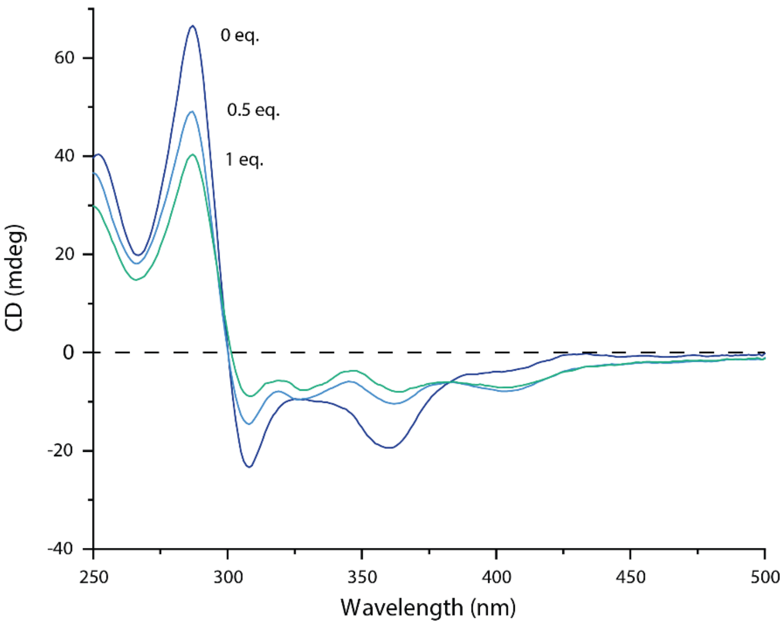

Figure S58: Partial data of the titration of $[Pd_2(R)$-**BAAZU-2**$)_4](BArF_{20})_4$ by $(NBu_4)_2[Pt(CN)_6]$ in CD_3CN measured by CD.

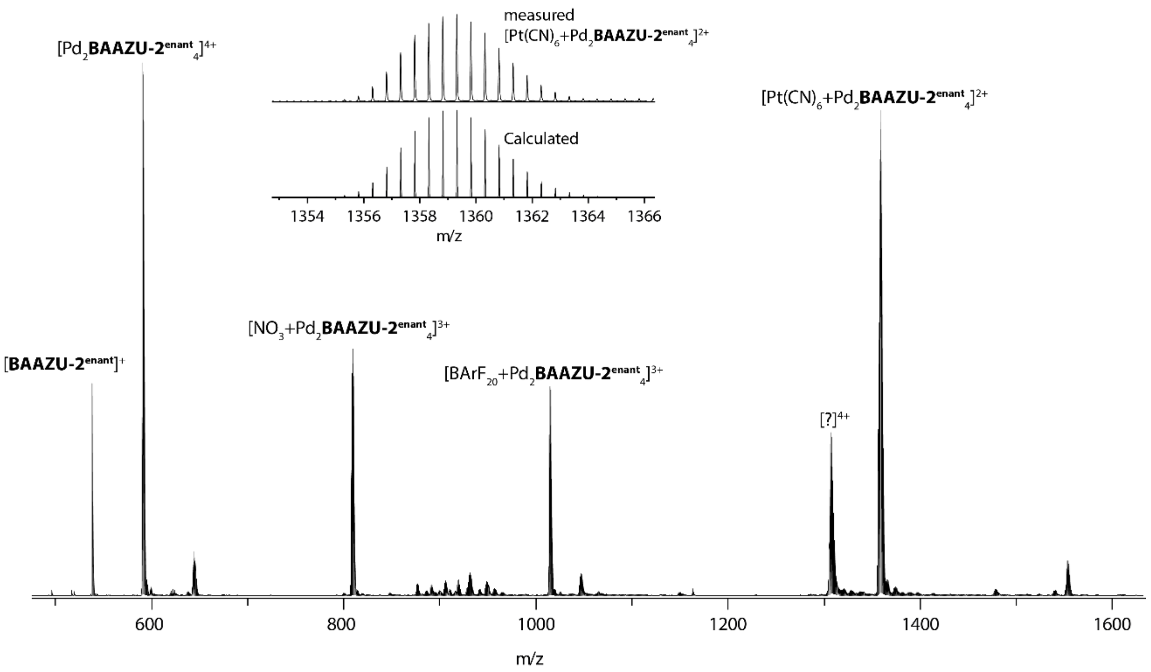

Figure S59: ESI-MS spectrum of host-guest complex $[Pt(CN)_6@Pd_2$**BAAZU-2**$^{enant}{}_4](BArF_{20})_2$

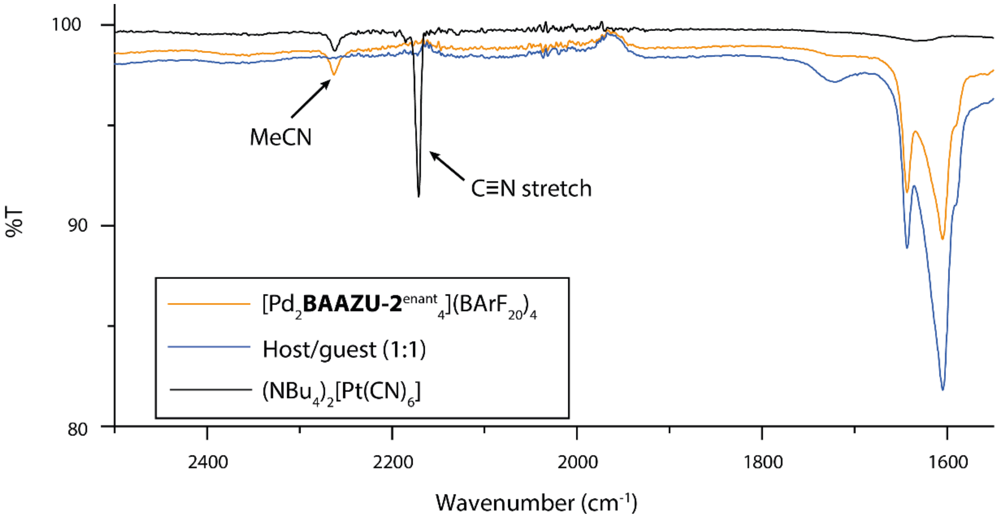

Figure S60: FT-IR spectra of $(NBu_4)_2[Pt(CN)_6]$ (black), cage [Pd$_2$**BAAZU-2**$^{\mathbf{enant}}$$_4$](BArF$_{20}$)$_4$ (orange), and the 1:1 host-guest complex (blue).

5.3. Camphorsulfonate (tetrabutyl ammonium salt)

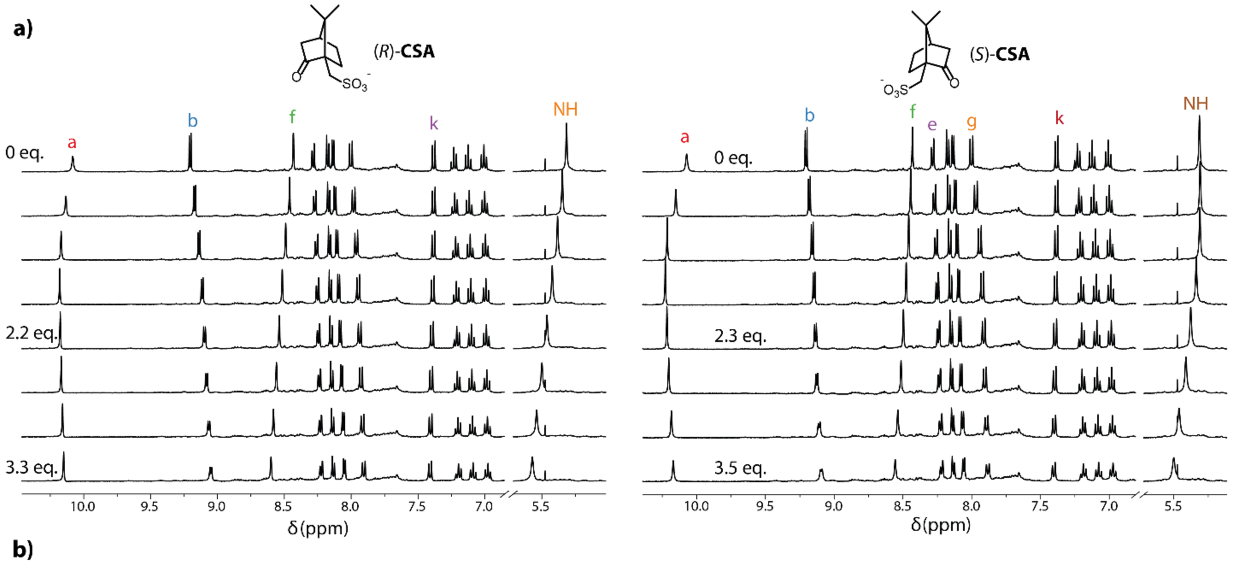

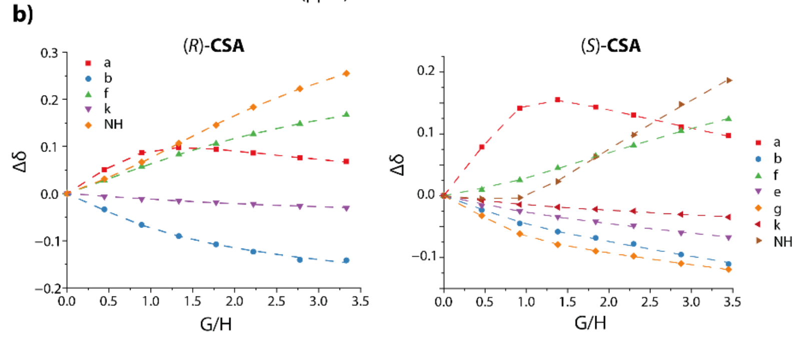

c) [Pd$_2$(*S*)-**BAAZU-2**$_4$](BArF$_{20}$)$_4$

	K_{11}	K_{21}
(*R*)-**CSA**	46’774 M^{-1}± 12%	1055 M^{-1}± 2%
(*S*)-**CSA**	150’260 M^{-1}± 16%	195 M^{-1}± 1%

Figure S61: (a) ^{1}H-NMR (500 MHz, CD_3CN, 298 K) of the titration of [Pd$_2$(*S*)-**BAAZU-2**$_4$](BArF$_{20}$)$_4$ with (*R*)- and (*S*)-**CSA** (tetrabutyl ammonium salt). The equivalents in guest were corrected through integration of the signals of tetrabutylammonium cation and of the cage. (b) Graph of the shifts of the signals of both titrations (scatter) and the BindFit fitting curve (dashed lines). (c) Binding constants of the two enantiomers of **CSA** to the cage [Pd$_2$(*S*)-**BAAZU-2**$_4$](BArF$_{20}$)$_4$.

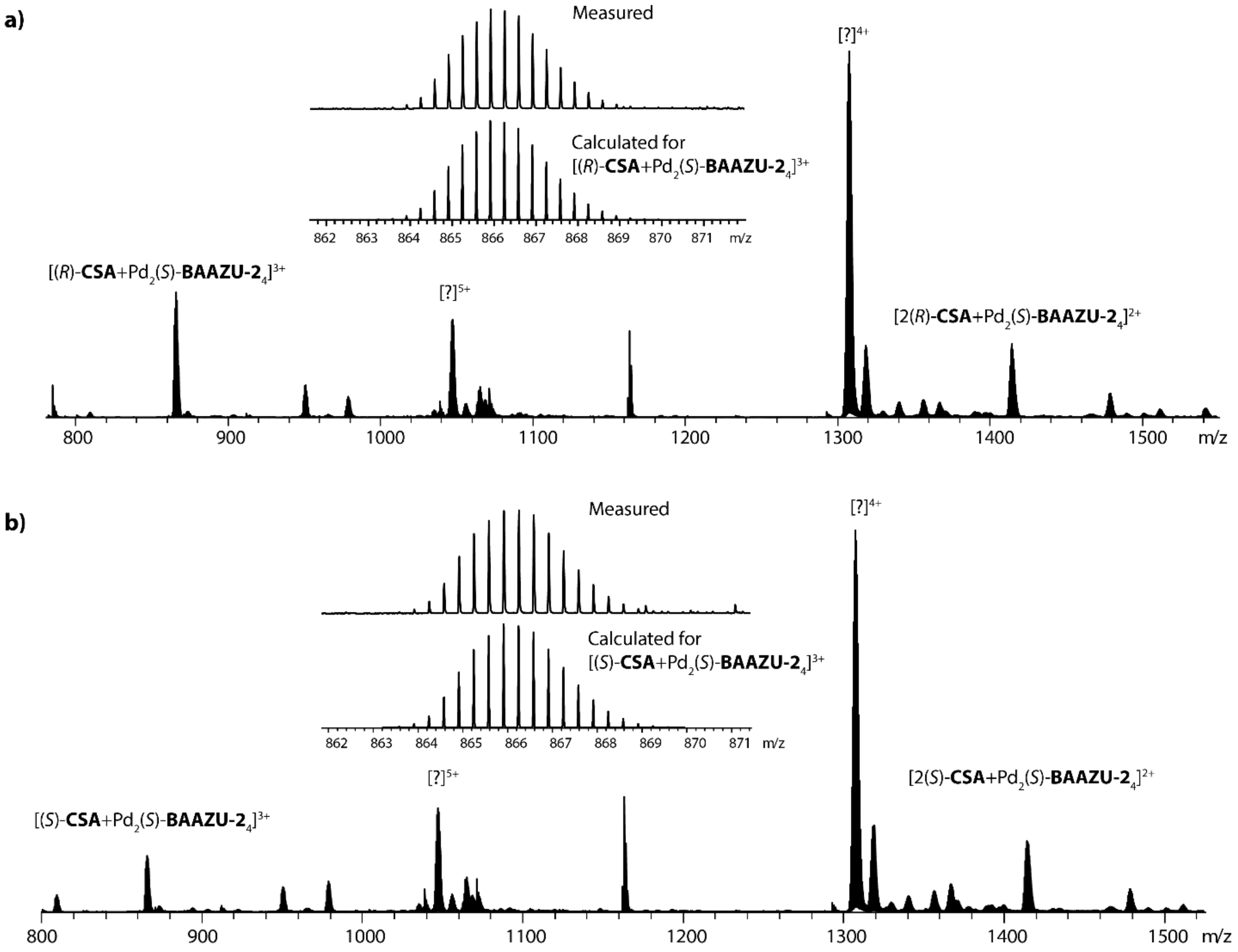

Figure S62: ESI-MS spectra of the host-guest complexes of cage $[Pd_2(S)$-**BAAZU-2**$_4](BArF_{20})_4$ with (*R*)- and (*S*)-**CSA** ((a) and (b), respectively). A larger species was detected as well, but could not be attributed to an obvious structure.

6. Computations

6.1. Computational investigation of the energetic ranking of the isomers

All geometries of the considered structures have been relaxed at the ωB97X-D3[7]/def2-SVP[8,9] level of theory and harmonic frequencies have been evaluated at the same level of theory in order to verify that the obtained structure is a minimum. When necessary, the harmonic frequencies have been employed to estimate the Gibbs free energy. In order to have accurate single point energies the DSD-PBEP86 D3BJ[10,11]/def2-TZVP[8,9] double hybrid functional has been employed[10,118,9]. The CPCM implicit solvation model[12] was used for both MeCN and DMSO; both solvents deliver the same energetic ranking. For this reason, we have employed only the MeCN implicit solvation model throughout the investigation. Below an example of the ORCA input file for the single point calculations.

```
! DSD-PBEP86 D3BJ
! def2-TZVP def2-TZVP/C
! CPCM(ACETONITRILE)
%MAXCORE 9200
%PAL NPROCS 32 END
%cpcm
surfacetype vdw_gaussian
end
* xyzFILE 4 1  input.xyz
```

Where indicated, the eight explicit solvent molecules were placed in the cage structure according to the crystal structures of the *RSRS* and *RRRR* isomers and at similar positions around the two other isomers. Both MeCN and DMSO solvent molecules were taken into account, based on the crystallographic data.

Table S2: Relative energies of the four different isomers of cage Pd_2**BAAZU-1**$_4$ with implicit and explicit solvation models.

Isomers	ΔE (kcal / mol)				ΔG (kcal / mol)		
	Implicit Solvent (MeCN)	8 MeCN	8 DMSO	4 MeCN + 4 DMSO	8 MeCN	8 DMSO	4 MeCN + 4 DMSO
RRRR	0	0	3	0	0	3	2
RRSS	8	7	11	13	4	9	12
RSRS	7	3	0	0	1	0	0
SRRR	4	6	7	8	6	7	5

Introducing explicit solvent molecules in the calculations significantly improves the agreement between the isomers energetic ranking and the experimental observations: the *RRRR* and *RSRS* cages are the two most stable isomers whereas the other two isomers are higher in energy, confirming the ^{1}H-NMR experiments.

In order to further evaluate which of the two isomers is the most stable, we increased the complexity of our computational models by introducing the BF_4^- anion in the structures of the two isomers. The position of the anion was chosen accordingly to the X-ray structures of the two isomers: in the *RRRR* isomer, the anion is located within the cage cavity and for the *RSRS* isomer it is positioned outside. In order to compare the isomers on the same ground, i.e. having a molecule in the cage cavity for both of them, we consider an additional MeCN molecule, and we position it inside the *RSRS* cage (as observed in the X-ray structure) and on top of the *RRRR* cage, interacting with the Pd centre.

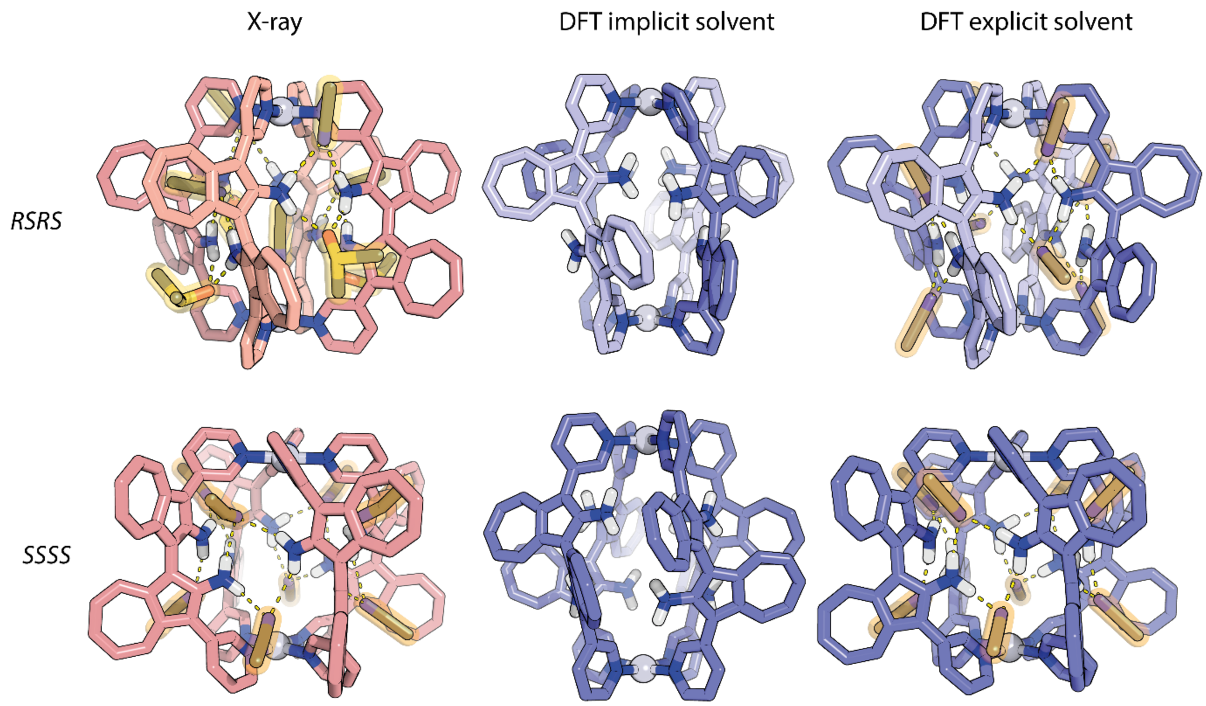

Figure S63: Comparison of the calculated structures of the *RRRR* and *RSRS* isomers in implicit and explicit solvent with the measured x-ray structures (only hydrogen-bonded solvents shown, encapsulated solvent in *RSRS* and encapsulated BF_4^- in *SSSS* not shown).

Table S3: relative energies of the *RRRR* and *RSRS* isomers of cage Pd$_2$**BAAZU-1**$_4$ with eight explicit hydrogen-bonded solvent molecules, one additional solvent molecule (inside or outside close to the Pd(II) site) and one additional BF_4^- anion (inside or outside close to the Pd(II) site).

Isomers	**ΔE (kcal / mol)**	**ΔG (kcal / mol)**
	8 MeCN + BF_4^- + 1 MeCN	8 MeCN + BF_4^- + 1 MeCN
RRRR	5	7
RSRS	0	0

The energies of the final models with a total of nine MeCN molecules and one counter-ion are in agreement with the experimental observation that the *RSRS* isomer is the most stable form.

6.2. Computational investigation of the ligand isomerisation

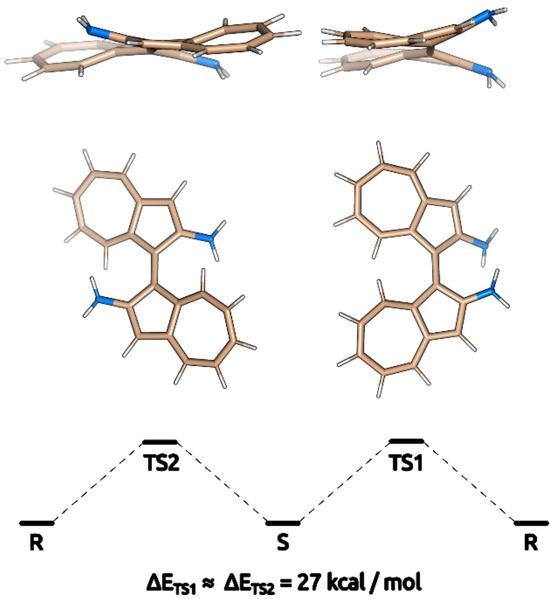

Figure S64: Calculated transition state geometries of the racemisation of **BAAZU** and their relative energies.

During the investigation, all geometries of the considered structures have been optimised at the same level of theory of section 6.1 in acetonitrile (treated with an implicit solvation model).[12] All transition states (TS), firstly located with the aid of NEB calculations[13], have been further optimised with the previously mentioned computational protocol. All transition state structures have one imaginary frequency.

Single point energies have been evaluated at the DLPNO-CCSD(T)[14]/def2-TZVPP level of theory in order to accurately estimate the energy barriers associated with the isomerisation process. Below an example of the ORCA input file concerning the single point calculations.

```
! DLPNO-CCSD(T) TightSCF TightPNO RIJCOSX NoPop

! def2-TZVPP def2-TZVPP/C

! CPCM(ACETONITRILE)

%MAXCORE 24000

%PAL NPROCS 64 END

* xyzFILE 0 1  input.xyz
```

First, the isolated **BAAZU** moiety was considered. The isomerisation between these two states can occur via two different transition states (Fig. S58). Both calculated energy barriers are 27 kcal/mol, suggesting that the two isomerisation pathways are isoenergetic. Nevertheless, the hypothetical formation of TS2 in the cage would require a large conformational rearrangement of the whole cage. For this reason, we infer that the isomerisation in the cage is preferred to occur through TS1. Then, the isomerisation of the full **BAAZU-1** ligand has been considered, yielding an energy barrier of 28 kcal/mol. This number is similar (within the chemical accuracy of 1 kcal/mol) to the **BAAZU** moiety considered before, suggesting that the energy barrier of the isomerisation is governed by the steric hindrance of the **BAAZU** unit in the TS.

Eventually, a reduced model of the cage was designed, with each pyridine donor coordinated to a terpy-Pd(II) fragment to simulate the effect of metal coordination on the electronic structure of the π-conjugated ligand. The same model has been studied experimentally. The transition state for the reduced model was considered (picture below, distance between the Pd atoms is highlighted in Angstrom) with a BF_4^- anion, as is present also in the experiments, leading to an energy barrier of 28 kcal/mol. These computations show that the perturbation of the electronic structure of the **BAAZU-1** ligand due to the palladium does not affect the isomerisation barrier. Hence, the experimentally observed rate acceleration of the cage isomerisation does not originate from a metal-induced perturbation of the electronic structure.

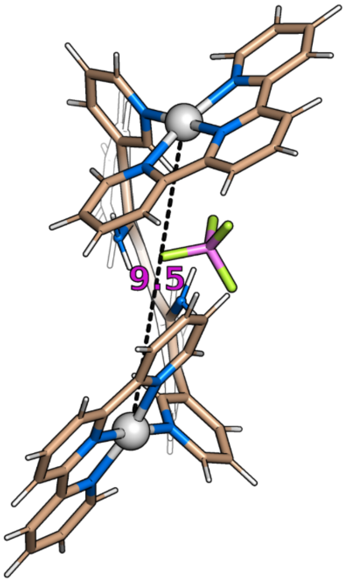

Figure S65: Model of the {[Pd_2**BAAZU-1**(terpy)$_2$](BF_4)}$^{3+}$ complex used for the racemisation energy barrier calculation.

6.3. Calculation of ECD spectra

The ECD of intermediate **8**, **BAAZU-1**, and **BAAZU-2** were calculated from DFT models of the compounds optimised at the ωB97X-D3/def2-TZVP level of theory. TD-DFT was performed with the same basis set, with both ωB2PLYP[15] and BHandHLYP[16] functionals. The resulting spectra were obtained through gaussian broadening. Below an example of the ORCA input file concerning the TD-DFT calculations.

```
! wB2PLYP Def2-TZVP VeryTightSCF

! AUTOAUX

! CPCM(ACETONITRILE)

! LARGEPRINT

%TDDFT

    Mode sTDDFT

    NROOTS  25
```

```
    TDA     FALSE
END
%MAXCORE 8000
%PAL NPROCS 32 END
%cpcm
surfacetype vdw_gaussian
end
* xyzFILE 0 1  input.xyz
```

The absolute configuration of both HPLC fractions intermediate **8** could be assigned through x-ray crystallography (see Section 7). Both functionals correctly predicted the sign of the weak S_0-S_1 transition in the visible range (450-700 nm). ωB2PLYP more accurately predicted the wavelength of the transition. However, in the UV range (250-400 nm) only BHandHLYP yielded the correct relative shifts and intensities of the higher energy bands.

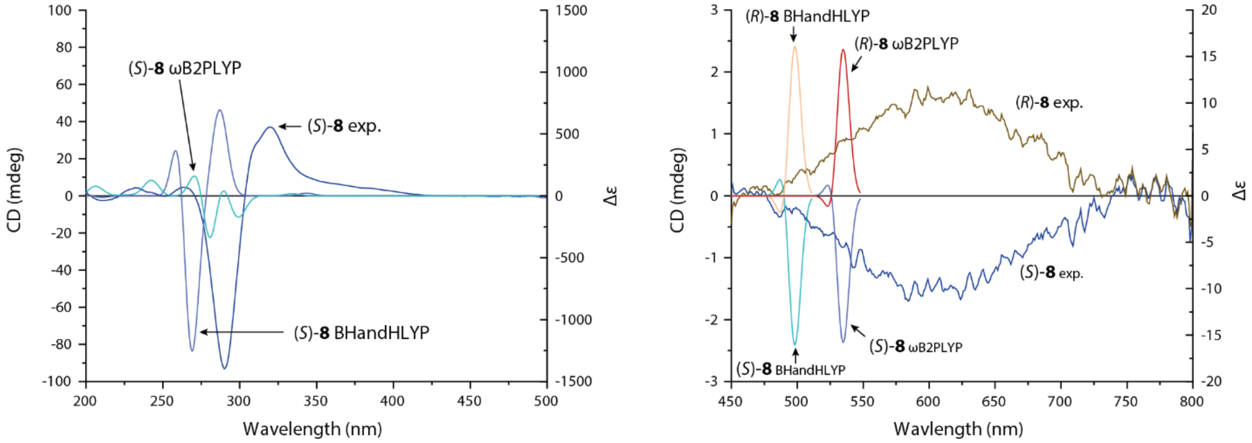

Figure S66: Comparison between the experimental CD of enantiopure **8** and the ECD calculated at the def2-TZVP/ ωB2PLYP or BHandHLYP level of theory

Fraction 1 of **BAAZU-1** could be assigned to the *R* enantiomer by x-ray crystallography (see section 7). Both functionals correctly predicted the sign of the weak S_0-S_1 transition in the visible range (450-700 nm). As with intermediate **8**, ωB2PLYP had an advantage in the prediction of the wavelength of the transition. In the UV range (250-400 nm), both functionals correctly predicted the sign and the wavelength of the transition at 290 nm, and of the overall shape of the spectrum from approx. 270-450 nm, with an advantage to BHandHLYP for a more correct relative intensity of the bands.

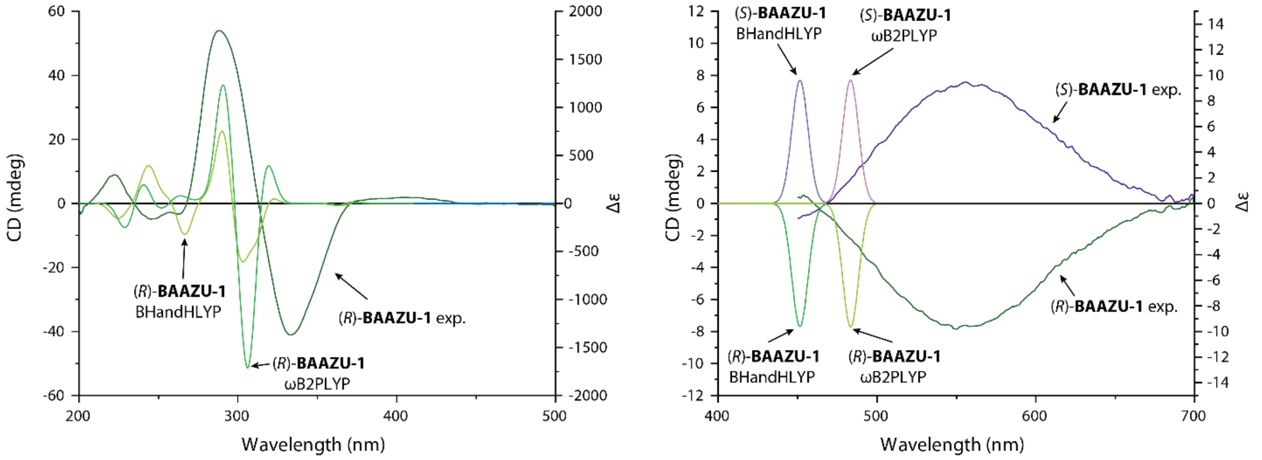

Figure S67: Comparison between the experimental CD of enantiopure **BAAZU-1** and the ECD calculated at the def2-TZVP/ωB2PLYP or BHandHLYP levels of theory.

In the absence of reliable data for the absolute configuration assignment of **BAAZU-2** by x-ray crystallography, TD-DFT was used exclusively to assign the enantiomers to the fractions of chiral HPLC, this method having been shown to work well for the two examples shown above. Fraction 1 was assigned to (*S*)-**BAAZU-2**, while fraction 2 was assigned to (*R*)-**BAAZU-2.**

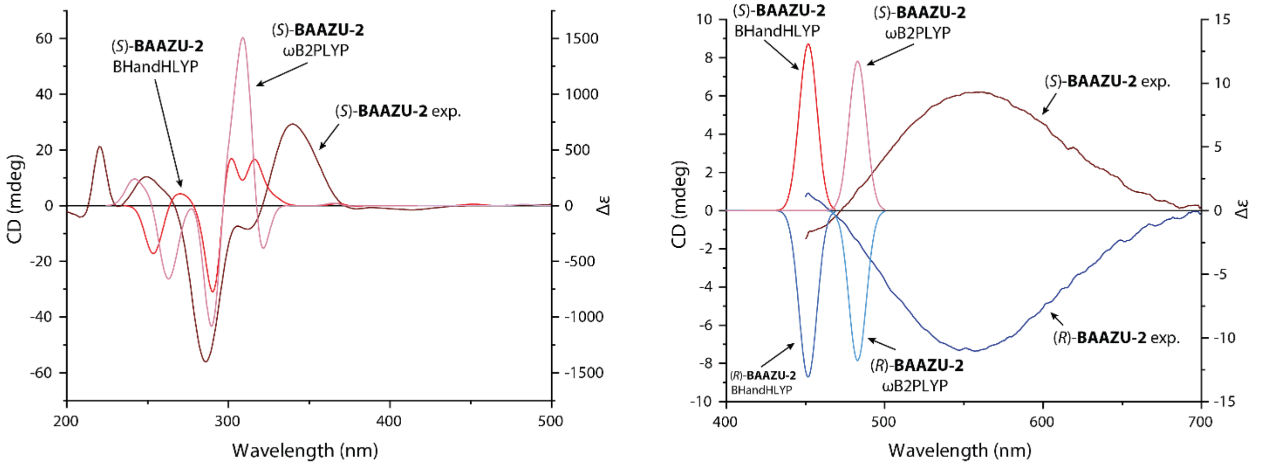

Figure S68: Comparison between the experimental CD of enantiopure **BAAZU-2** and the ECD calculated at the def2-TZVP/ωB2PLYP or BHandHLYP levels of theory.

6.4. BAAZU-2 cage and host-guest complexes

The models of the host-guest systems are based on an initial model constructed in Wavefunction SPARTAN'18,[17] and then pre-optimised by the semi-empirical PM6 method in Gaussian 16.[18] The final DFT model was geometry-optimised in the gas-phase at the ωB97X-D/def2-SVP level. The models as .xyz files can be found as supplementary data.

Due to convergence issues when using Gaussian, the cage-**CSA** host-guest complexes were optimised in Orca, at the ωB97X-D3/def2-SVP level.

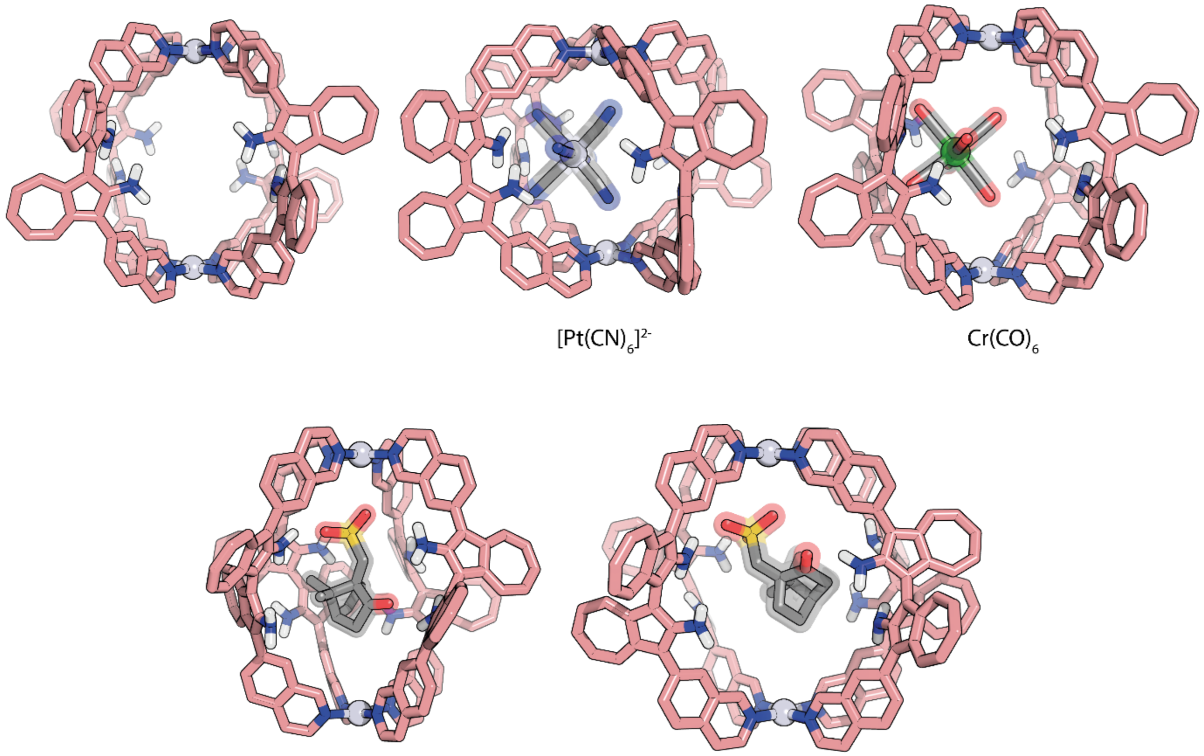

Figure S69: DFT-optimised structures of the *RRRR* Pd_2**BAAZU-2**$_4$ cage and of its four host-guest complexes.

6.5. Volumes of the cavities

The volumes of the following three cages' cavities were calculated by MoloVol,[19] using the X-ray structures for the first two species, and DFT with the third one. Size of the probe was chosen to be 2 Å for every calculation. No second larger external probe was used.

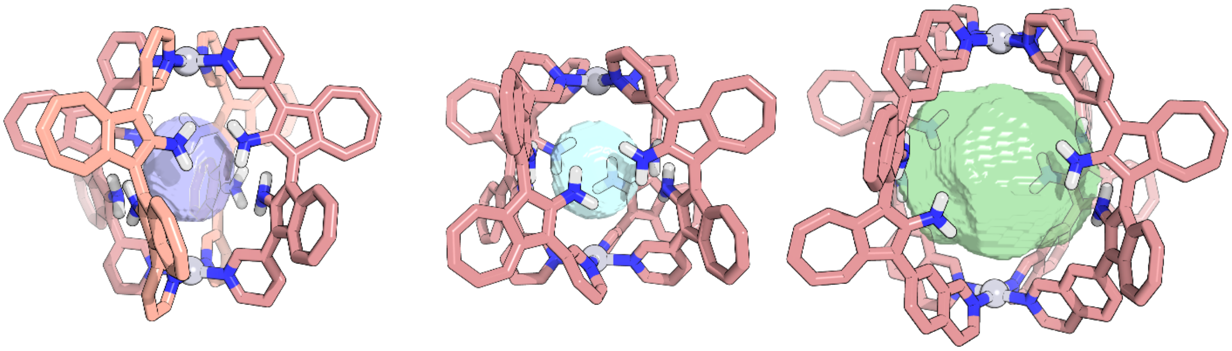

Pd_2**BAAZU-1**$^{rac}_4$ V_{cav}=60 Å^3 | Pd_2**BAAZU-1**$^{enant}_4$ V_{cav}=63 Å^3 | Pd_2**BAAZU-1**$^{rac}_4$ V_{cav}=414 Å^3

Figure S70: cavities of the cages, as calculated with MoloVol, with a 2 Å probe.

7. X-ray crystallography

Six compounds were studied by single crystal X-ray crystallography: **BAAZU** (**6**), **BAAZU-1 fraction 1**, **BAAZU-2 fraction 2**, diisobutyl (3,3'-dibromo-[1,1'-biazulene]-2,2'-diyl)dicarbamate (**8**) fractions 1 & 2, Pd_2**BAAZU-1**^{rac} and Pd_2(*S*)-**BAAZU-1**$_4$. Crystals of suitable quality and size to be measured in-house were obtained for **BAAZU** (**6**), **8** fractions 1 & 2, **BAAZU-1 fraction 1**, Pd_2**BAAZU-1**$^{rac}{}_4$ and Pd_2(*S*)-**BAAZU-1**$_4$. They were measured on a Bruker D8 venture diffractometer equipped with an INCOATEC microfocus sealed tube (Iµs 3.0) at 100 K. The data was integrated with APEX3 and the structure was solved by intrinsic phasing/direct methods using SHELXT[20] and refined with SHELXL[21] for full-matrix least-squares routines on F^2 and ShelXle[22] as a graphical user interface.

Small crystals of **BAAZU-2 fraction 2** were measured at the macomolecular BioMAX beamline of the MAXIV synchrotron, in Lund, Sweden.

Table S4: crystallographic tables of **BAAZU** and **BAAZU-1 F1**

**Compound**	**BAAZU (6)**	(*R*)-**BAAZU-1**
**CIF ID**	aw35high	aw117_f1_j
**CCDC number**	2356504	2356507
Empirical formula	$C_{20}H_{16}N_2$	$C_{30}H_{22}N_4$
Formula weight	284.35	438.51
Temperature [K]	100(2)	100(2)
Crystal system	orthorhombic	monoclinic
Space group (number)	$Pca2_1$ (29)	$P2_1$ (4)
a [Å]	19.9343(7)	8.5359(2)
b [Å]	11.1774(4)	23.0569(6)
c [Å]	6.7622(2)	11.3375(3)
α [°]	90	90
β [°]	90	90.0470(10)
γ [°]	90	90
Volume [$Å^3$]	1506.71(9)	2231.35(10)
Z	4	4
ρ_{calc} [gcm^{-3}]	1.254	1.305
μ [mm^{-1}]	0.074	0.610
F(000)	600	920
Crystal size [mm^3]	0.150×0.150×0.150	0.500×0.120×0.120
Crystal colour	red	brown
Crystal shape	block	block
Radiation	MoK_α (λ=0.71073 Å)	CuK_α (λ=1.54178 Å)
2θ range [°]	4.09 to 125.42 (0.40 Å)	3.83 to 159.91 (0.78 Å)
Index ranges	−49 ≤ h ≤ 45<br>−27 ≤ k ≤ 27<br>−16 ≤ l ≤ 15	−9 ≤ h ≤ 10<br>−29 ≤ k ≤ 29<br>−14 ≤ l ≤ 14
Reflections collected	190669	124991
Independent reflections	22926<br>R_{int} = 0.0724<br>R_{sigma} = 0.0330	9595<br>R_{int} = 0.0371<br>R_{sigma} = 0.0144
Completeness to θ = 67.679°	99.7 %	100.0 %
Data / Restraints / Parameters	22926/1/263	9595/1/746
Goodness-of-fit on F^2	1.109	1.024
Final *R* indexes [*I*≥2σ(*I*)]	R_1 = 0.0457<br>wR_2 = 0.1103	R_1 = 0.0246<br>wR_2 = 0.0632
Final *R* indexes [all data]	R_1 = 0.0567<br>wR_2 = 0.1182	R_1 = 0.0248<br>wR_2 = 0.0634
Largest peak/hole [$eÅ^{-3}$]	0.60/−0.28	0.14/−0.17
Flack X parameter	-	0.05(8)

Table S5: crystallographic tables of **8 F1** and **8 F2**

**Compound**	**8 F1**	**8 F2**
**CIF ID**	aw143f1	aw143f2
**CCDC number**	2356506	2356502
Empirical formula	$C_{30}H_{30}Br_2N_2O_4$	$C_{30}H_{30}Br_2N_2O_4$
Formula weight	642.38	642.38
Temperature [K]	100(2)	100(2)
Crystal system	orthorhombic	orthorhombic
Space group (number)	$P2_12_12_1$ (19)	$P2_12_12_1$ (19)
a [Å]	9.4436(5)	9.4452(5)
b [Å]	12.2584(7)	12.2541(6)
c [Å]	24.0877(14)	24.1005(13)
α [°]	90	90
β [°]	90	90
γ [°]	90	90
Volume [$Å^3$]	2788.5(3)	2789.5(3)
Z	4	4
ρ_{calc} [gcm^{-3}]	1.530	1.530
μ [mm^{-1}]	3.999	3.998
F(000)	1304	1304
Crystal size [mm^3]	0.100×0.050×0.050	0.200×0.050×0.050
Crystal colour	blue	blue
Crystal shape	block	block
Radiation	CuK_α (λ=1.54178 Å)	CuK_α (λ=1.54178 Å)
2θ range [°]	7.34 to 148.98 (0.80 Å)	7.34 to 158.03 (0.79 Å)
Index ranges	−11 ≤ h ≤ 9<br>−15 ≤ k ≤ 15<br>−30 ≤ l ≤ 30	−8 ≤ h ≤ 11<br>−15 ≤ k ≤ 15<br>−30 ≤ l ≤ 29
Reflections collected	56365	51795
Independent reflections	5694<br>R_{int} = 0.0415<br>R_{sigma} = 0.0224	5975<br>R_{int} = 0.0424<br>R_{sigma} = 0.0271
Completeness to<br>θ = 67.679°	100.0 %	100.0 %
Data / Restraints / Parameters	5694/537/353	5975/0/349
Goodness-of-fit on F^2	1.026	1.061
Final *R* indexes<br>[$I \geq 2\sigma(I)$]	R_1 = 0.0179<br>wR_2 = 0.0447	R_1 = 0.0218<br>wR_2 = 0.0542
Final *R* indexes<br>[all data]	R_1 = 0.0183<br>wR_2 = 0.0449	R_1 = 0.0225<br>wR_2 = 0.0546
Largest peak/hole [$eÅ^{-3}$]	0.25/−0.40	0.58/−0.48
Flack X parameter	0.044(4)	0.059(5)

Table S6: crystallographic tables of Pd_2**BAAZU-1**$^{rac}{}_4$ and Pd_2(*S*)-**BAAZU-1**$_4$

**Compound**	Pd_2**BAAZU-1**$^{rac}{}_4$	Pd_2(*S*)-**BAAZU-1**$_4$
**CIF ID**	aw82j	aw98j
**CCDC number**	2356505	2356503
Empirical formula	$C_{163}H_{169.09}B_4F_{16}N_{30.64}O_{4.86}Pd_2S_{3.86}$	$C_{140}H_{122}B_3F_{12}N_{24}OPd_2$
Formula weight	3318.98	2629.84
Temperature [K]	100(2)	100(2)
Crystal system	triclinic	triclinic
Space group (number)	$P\overline{1}$ (2)	$P1$ (1)
a [Å]	17.4805(6)	15.6000(8)
b [Å]	22.5706(8)	17.1715(10)
c [Å]	22.7915(8)	17.7204(9)
α [°]	92.3420(10)	96.809(2)
β [°]	100.2940(10)	115.622(2)
γ [°]	110.8380(10)	111.923(2)
Volume [Å^3]	8215.3(5)	3741.5(4)
Z	2	1
ρ_{calc} [gcm^{-3}]	1.342	1.167
μ [mm^{-1}]	2.904	2.502
F(000)	3437	1353
Crystal size [mm^3]	0.300×0.150×0.100	0.100×0.100×0.040
Crystal colour	brown	brown
Crystal shape	block	block
Radiation	CuK_α (λ=1.54178 Å)	CuK_α (λ=1.54178 Å)
2θ range [°]	3.97 to 158.47 (0.78 Å)	5.87 to 132.89 (0.84 Å)
Index ranges	−22 ≤ h ≤ 22<br>−28 ≤ k ≤ 28<br>−27 ≤ l ≤ 29	−15 ≤ h ≤ 18<br>−18 ≤ k ≤ 18<br>−21 ≤ l ≤ 18
Reflections collected	190543	93122
Independent reflections	34821<br>R_{int} = 0.1654<br>R_{sigma} = 0.0754	18292<br>R_{int} = 0.0815<br>R_{sigma} = 0.0644
Completeness to<br>θ = 67.679°	100.0 %	74.1 %
Data / Restraints / Parameters	34821/3682/2289	18292/3423/1732
Goodness-of-fit on F^2	1.081	1.264
Final *R* indexes<br>[*I*≥2σ(*I*)]	R_1 = 0.0557<br>wR_2 = 0.1583	R_1 = 0.1030<br>wR_2 = 0.2742
Final *R* indexes<br>[all data]	R_1 = 0.0582<br>wR_2 = 0.1615	R_1 = 0.1226<br>wR_2 = 0.2940
Largest peak/hole [eÅ^{-3}]	1.40/−1.63	2.08/−0.81
Flack X parameter		0.228(5)

Table S7: crystallographic table of **BAAZU-2** fraction 2

**Compound**	**BAAZU-2** f2
**CIF ID**	aw139f2
**CCDC number**	2356508
Empirical formula	$C_{38}H_{26}N_4$
Formula weight	538.63
Temperature [K]	100(2)
Crystal system	monoclinic
Space group (number)	$P2_1$ (4)
a [Å]	14.2400(11)
b [Å]	7.1410(15)
c [Å]	15.859(4)
α [°]	90
β [°]	94.206(11)
γ [°]	90
Volume [Å^3]	1608.3(5)
Z	2
ρ_{calc} [gcm^{-3}]	1.112
μ [mm^{-1}]	0.047
F(000)	564
Crystal size [mm^3]	0.250×0.015×0.005
Crystal colour	brown
Crystal shape	needle
Radiation	synchrotron (λ=0.5904 Å)
2θ range [°]	2.14 to 50.48 (0.69 Å)
Index ranges	−16 ≤ h ≤ 16<br>−8 ≤ k ≤ 8<br>−18 ≤ l ≤ 18
Reflections collected	45807
Independent reflections	7079<br>R_{int} = 0.0341<br>R_{sigma} = 0.0222
Completeness to<br>θ = 67.679°	97.0 %
Data / Restraints / Parameters	7079/668/391
Goodness-of-fit on F^2	0.822
Final *R* indexes<br>[I≥2σ(I)]	R_1 = 0.0519<br>wR_2 = 0.1686
Final *R* indexes<br>[all data]	R_1 = 0.0549<br>wR_2 = 0.1789
Largest peak/hole [eÅ^{-3}]	0.17/−0.26
Flack X parameter	−4.3(10)

7.1. BAAZU (6)

Brown block-shaped crystals of **6** (racemate) were grown by slow vapour diffusion of n-pentane into a DCM solution of **6** at room temperature. Single crystals in mother liquor were pipetted onto a glass slide containing NVH oil. To avoid collapse of the crystal lattice, the chosen crystal was quickly mounted on a micro loop and mounted on the diffractometer under a flow of N_2 at 100 K. The data collection was performed using a MoK_α radiation source.

Specific refinement details

The compound crystallises in the orthorhombic $Pca2_1$ (29) space group, with one molecule per asymmetric unit cell, and four per unit cell. Every hydrogen atom could be resolved.

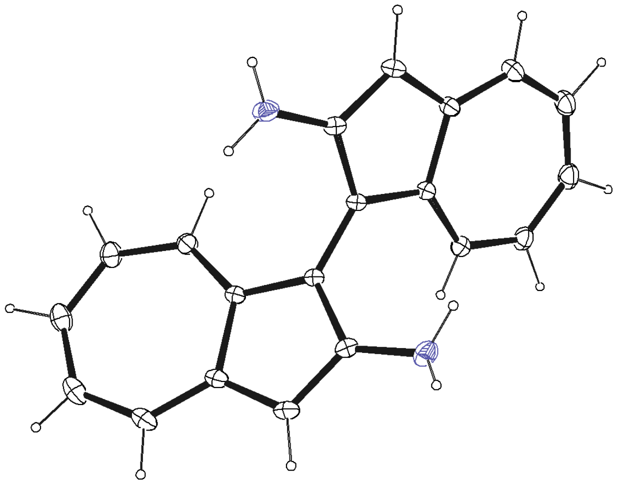

Figure S71: ORTEP representation of **BAAZU**. Ellipsoids drawn at 50% probability.

7.2. BAAZU-1 Fraction 1

Brown block-shaped crystals of **BAAZU-1** (fraction 1) were grown by slow vapour diffusion of diethyl ether (Et_2O) into a DCM solution of the ligand at room temperature. Crystals in mother liquor were pipetted onto a glass slide containing NVH oil and the chosen crystal was quickly mounted on a micro loop and mounted on the diffractometer under a flow of N_2 at 100 K. The data collection was performed using a CuK_α radiation source.

Specific refinement details

The compound crystalises in the monoclinic $P2_1$ (4) space group. Two ligands are present in the asymmetric unit cell. Twinning of the crystal was taken into account with the following instructions:

TWIN -1 0 0 0 -1 0 0 0 1

BASF 0.41872

A Flack parameter of 0.05(8) was measured according to Parson's method. Therefore, the absolute configuration of fraction 1 of the ligand was assigned to the *R* enantiomer. This result was further confirmed by comparison of the experimental and DFT-calculated CD spectra (see section 8).

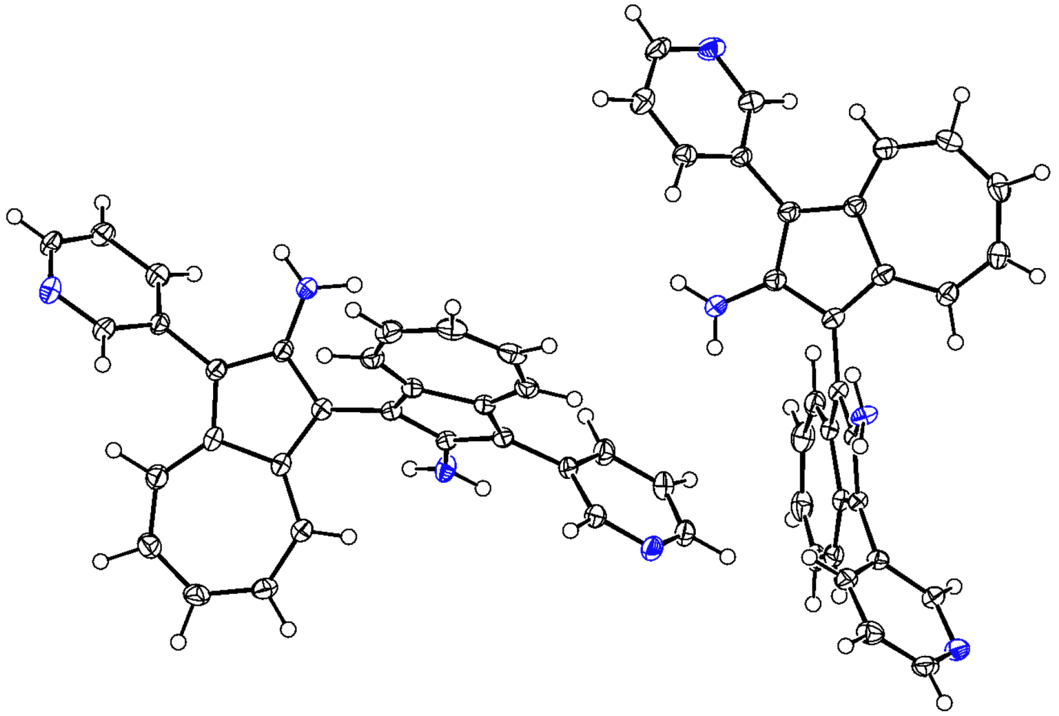

Figure S72: ORTEP representation of the asymmetric cell of (*R*)-**BAAZU-1**. Ellipsoids drawn at 50% probability.

7.3. Diisobutyl (3,3'-dibromo-[1,1'-biazulene]-2,2'-diyl)dicarbamate (8) fractions 1 & 2

Blue block-shaped crystals of intermediate **8** (the two enantiomers separated by chiral HPLC) were grown by layering a DCM solution of the title compound with n-hexane at 4°C. Crystals in mother liquor were pipetted onto a glass slide containing NVH oil and the chosen crystal was quickly mounted on a micro loop and then mounted on the diffractometer under a flow of N2 at 100 K. The data collection was performed using a CuK_{α} radiation source.

Specific refinement details

The compound crystalises in the orthorhombic $P2_12_12_1$ (19) space group, with one molecule per asymmetric unit cell. Most hydrogens were added through the **AFIX** instruction, except for the two NH groups.

Fraction 1 was assigned to the *S* enantiomer, with a Flack parameter according to Parson's method of 0.044 (4). Fraction 2 was assigned to the *R* enantiomer, with a Flack parameter according to Parson's method of 0.059 (5).

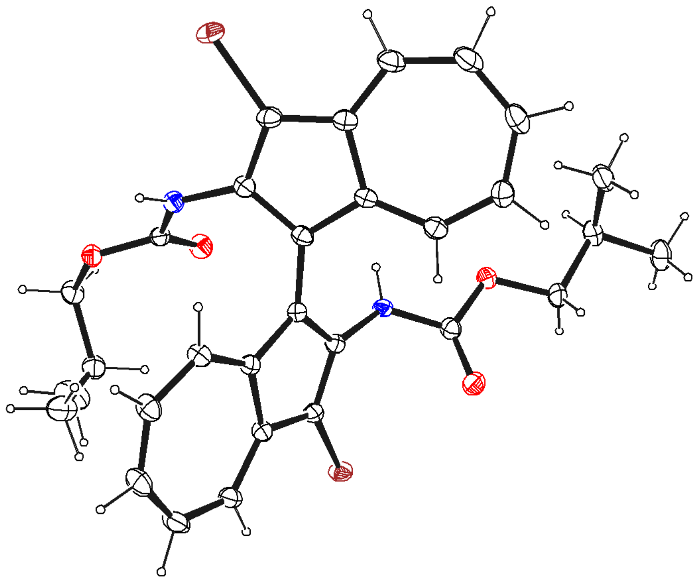

Figure S73: ORTEP representation of (*S*)-**8** (fraction 1). Ellipsoids drawn at 50% probability.

7.4. [Pd_2BAAZU-1$^{rac}{}_4$](BF_4)$_4$

Brown block-shaped crystals of [Pd_2**BAAZU-1**$^{rac}{}_4$](BF_4)$_4$ were grown by slow vapour diffusion of diisopropylether (iPr_2O) into an acetonitrile (ACN) solution of the supramolecular species with 10% DMSO at room temperature. Single crystals in mother liquor were pipetted onto a glass slide containing NVH oil. To avoid collapse of the crystal lattice, the chosen crystal was quickly mounted on a micro loop and mounted on the diffractometer under a flow of N_2 at 100 K. The data collection was performed using a CuK_α radiation source.

Specific refinement details

Stereochemical restraints for dimethyl sulfoxide (residue DMS), acetonitrile (residue ACN), and diisopropyl ether (residue DIP) solvent molecules, and tetrafluoroborate (residue BF4) counter anion were generated by the GRADE program using the GRADE Web Server (http://grade.globalphasing.org) and applied in the refinement. A GRADE dictionary for SHELXL contains target values and standard deviations for 1,2-distances (DFIX) and 1,3-distances (DANG), as well as restraints for planar groups (FLAT). All displacements for non-hydrogen atoms were refined anisotropically. The refinement of ADPs for every element except hydrogen was enabled by a combination of similarity restraints (SIMU) and rigid bond restraints (RIGU)[24].

The compound crystallised in the triclinic space group $P\overline{1}$ (2). The cage was modelled in its entirety, and the protons of the amino groups could be placed manually. Those amino groups are bridged by DMSO and ACN molecules. Those solvents molecules overlap in two positions, necessitating to be modelled as disordered over the same positions using free individual variables for refinement of occupancy factors. Moreover, three out of the four BF4 residues had to be modelled as disordered over two conformations using free individual variables for refinement of occupancy factors. Finally, one ACN residue had to be modelled on a special position, with half-occupancy.

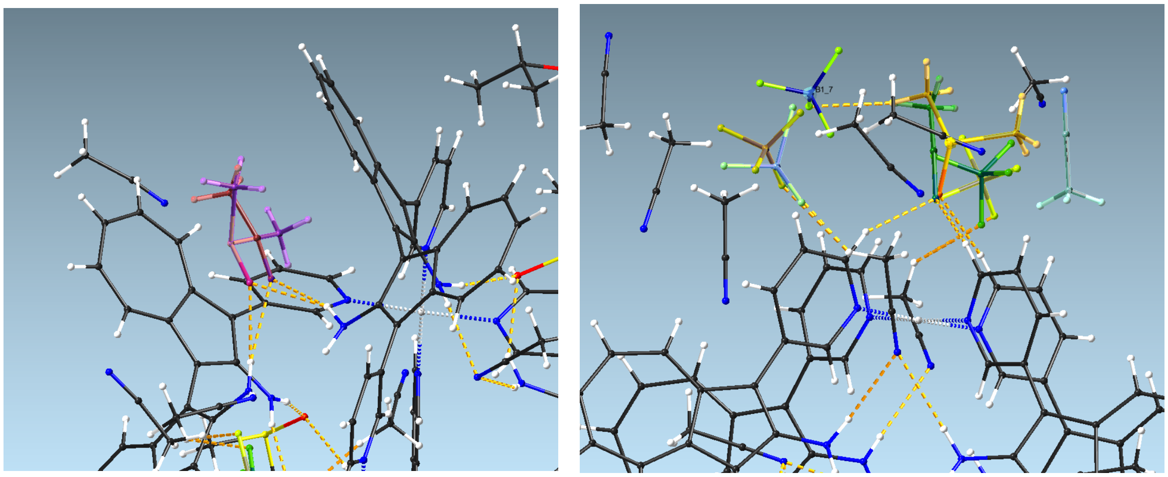

Figure S74: Disorders in some of the solvent and counteranions in the X-ray structure of [Pd_2(**BAAZU-1**^{rac})$_4$](BF_4)$_4$

7.5. [Pd_2(*S*)-BAAZU-1_4](BF_4)$_4$

Brown block-shaped crystals of [Pd_2(*S*)-**BAAZU-1**$_4$](BF_4)$_4$ (fraction 2 of **BAAZU-1**) were grown by slow vapour diffusion of diethylether (Et_2O) into an acetonitrile (ACN) solution of the supramolecular species prepared with fraction 2 at room temperature. Single crystals in mother liquor were pipetted onto a glass slide containing NVH oil. To avoid collapse of the crystal lattice, the chosen crystal was quickly mounted on a micro loop and mounted on the diffractometer under a flow of N_2 at 100 K. The data collection was performed using a CuK_α radiation source.

Specific refinement details

Stereochemical restraints for the **BAAZU-1** ligand (residue BAA), acetonitrile (residue ACN) and diethyl ether (residue ETO) solvent molecules, and tetrafluoroborate (residue BF4) counter anion were generated by the GRADE program using the GRADE Web Server (http://grade.globalphasing.org) and applied in the refinement. A GRADE dictionary for SHELXL contains target values and standard deviations for 1,2-distances (DFIX) and 1,3-distances (DANG), as well as restraints for planar groups (FLAT). All displacements for non-hydrogen atoms were refined anisotropically. The refinement of ADPs for every element except hydrogen was enabled by a combination of similarity restraints (SIMU) and rigid bond restraints (RIGU).[24] The contribution of the electron density from disordered counterions and solvent molecules, which could not be modelled with discrete atomic positions were handled using the SQUEEZE[25] routine in PLATON.[26] The solvent mask file (.fab), computed by PLATON, was included in the SHELXL refinement via the ABIN instruction leaving the measured intensities untouched.

The compound crystalised in the triclinic space group $P1$ (1), with one full cage per unit cell, as well as three BF4 counter anions and one diethy ether counter solvent molecule. One out of the three BF4 residues and the ETO residue had to be modelled as disordered over two conformations using free individual variables for refinement of occupancy factors. The measured Flack parameter according to Parson's method before SQUEEZE was 0.208 (5), with the ligands in the *S*-conformation.

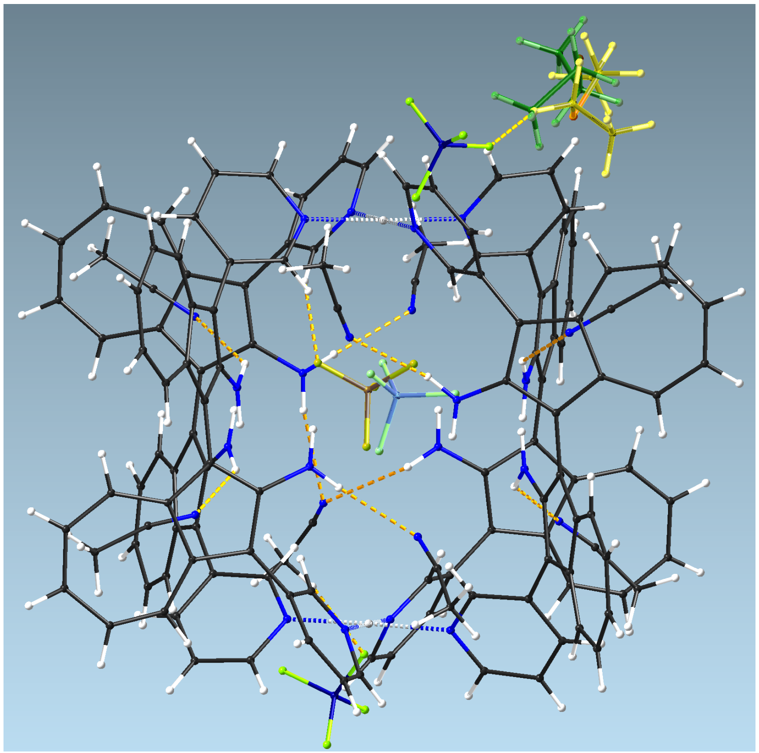

Figure S75: Full model of the cage, with cavity-bound disordered BF_4^- residue, and disordered diethyl ether counter solvent (top right).

7.6. BAAZU-2 Fraction 2

Brown needle-shaped of BAAZU-2 fraction 2 were grown by slow vapour diffusion of pentane into a DCM solution of the ligand at 4°C. Single crystals in mother liquor were pipetted onto a glass slide containing NVH oil. To avoid collapse of the crystal lattice, several crystals were quickly mounted on micro loops and immediately flash-cooled in liquid nitrogen. Crystals were stored at cryogenic temperature in dry shippers, in which they were safely transported to macromolecular beamline BioMAX[27] at the MAX IV synchrotron, Lund, Sweden. A wavelength of λ = 0.5904 Å was chosen using a liquid N_2 cooled double crystal monochromator. Single crystal X-ray diffraction data was collected at 100(2) K on Arinax MD3 microdiffractometer with minikappa axis, equipped with Oxford Cryostream 800, Eiger1 16M detector and Irelec Isara sample changing robot. 3600 diffraction images were collected in a single 360° ϕ sweep using local MXCube3[28] and ISPyB[29] implementation and a detector distance of 188.5 mm, 100% filter transmission, 0.1° step width and 40 milliseconds exposure time per image. Data integration and reduction were undertaken using autoProc[30] and XDS.[31]

Specific refinement details

All displacements for non-hydrogen atoms were refined anisotropically. The refinement of ADPs for every element except hydrogen was enabled by a combination of similarity restraints (SIMU) and rigid bond restraints (RIGU).[24] The contribution of the electron density from disordered counterions and solvent molecules, which could not be modelled with discrete atomic positions were handled using the SQUEEZE[25] routine in PLATON.[26] The solvent mask file (.fab), computed by PLATON, was included in the SHELXL refinement via the ABIN instruction leaving the measured intensities untouched.

The compound crystallised in the monoclinic space group $P2_1$ (4), with one molecule per asymmetric unit cell. Every hydrogen atom was added by the **AFIX** instruction. The crystal possesses voids occupied by disordered solvent molecules, amounting to approx. 14% of the total volume.

The Flack parameter was calculated to be −4.3(10), which is very high and hence does allow us to determine the absolute structure to derive the absolute configuration of the compound by X-ray crystallography.

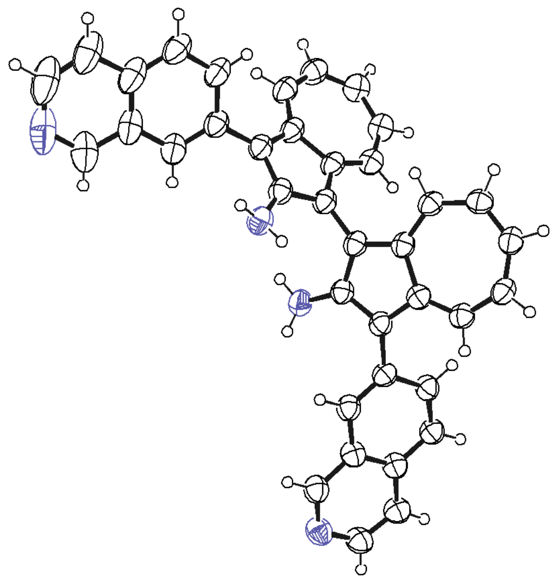

Figure S76: ORTEP representation of **BAAZU-2**. Ellipsoids drawn at 50% probability.

8. Assignment of the absolute configuration of BAAZU-derivatives

Due to the sparse examples of BAAZU-derivatives in the literature, the absolute configuration of the ligands **BAAZU-1** and **BAAZU-2** and by extension of their corresponding Pd_2L_4 cages had to be clearly determined. The only literature example of the absolute configuration determination of a bis-amino azulene derivative as far as we know was performed by Chen, Yen, Kuo, and Chen.[32] There, they used the chiral exciton coupling of CD to assign the *S*-form to a positive Cotton effect and the *R*-form to a negative Cotton effect.

We wished in turn to complement this analysis by our own measurements. The two techniques we used here were X-ray crystallography on one side, and comparison of experimental and computation CD spectra on the other.

Crystals of enantiopure **BAAZU-1** and **BAAZU-2** were successfully grown and measured by X-ray crystallography. Unfortunately, **BAAZU-1** was not able to be obtained as a single crystal, and therefore twinning had to be considered in the refinement of the data. See Section 7 for a detailed description of the refinement of the data. Fraction 1 of the ligand was assigned to the *R*-enantiomer, and by extrapolation Fraction 2 to the *S*-enantiomer. CD measurements showed a negative Cotton effect for Fraction 1 (*R)*, consistent with the work of Chen *et al.*[32]

TD-DFT computations were also performed for comparison with the experimental CD spectra. They also confirmed the assignment of the *R*-enantiomer of **BAAZU-1** to a negative Cotton effect.

Due to the small size of the crystals of enantiopure **BAAZU-2**, they had to be measured with synchrotron radiation. However, the Flack parameter could not be reliably obtained for this ligand. Interestingly, the Cotton effects of each corresponding fraction of **BAAZU-1** and **BAAZU-2** show inverse signs, suggesting that Fraction 1 of **BAAZU-2** corresponds to the *S*-enantiomer. The order of elution in the chiral HPLC column cannot therefore be used as a reliable assignment of the absolute configuration of BAAZU-derivatives. TD-DFT again confirmed that a negative Cotton effect is expected for the *R*-enantiomer.

Finally, the two enantiomers of intermediate **8** were separated, crystallised, and measured by X-ray crystallography. The analysis showed no twinning, and Fraction 1 could be assigned to *S*-**8**, and Fraction 2 to *R*-**8**. However, unlike the ligands already characterised, *S*-**8** showed a negative Cotton effect, which was confirmed by ECD. Therefore, in the three compounds studied here, there is no correlation between the order of elution or the sign of the Cotton effect, and the absolute configuration of the compound. However, TD-DFT was shown as a reliable tool for the assignment of the absolute configuration of the BAAZU-derivatives.

Table S8: Correspondence between the order of elution of the enantiomers of the three measured chiral compounds and of their absolute configuration and of the Cotton effect.

Compound	Intermediate **8**	**BAAZU-1**	**BAAZU-2**
Fraction 1	*S (negative)*	*R (negative)*	*S (positive)*
Fraction 2	*R (positive)*	*S (positive)*	*R (negative)*

9. References

(1) Li, M.; Wang, D.-H. Copper-Catalyzed 3-Positional Amination of 2-Azulenols with O-Benzoylhydroxylamines. *Org. Lett.* **2021**, *23* (17), 6638–6641. https://doi.org/10.1021/acs.orglett.1c02132.

(2) Nozoe, T.; Kitahara, Y.; Takase, K.; Sasaki, M. Syntheses of Benzo [b] Tropazines. *Proc. Jpn. Acad.* **1956**, *32* (5), 349–352. https://doi.org/10.2183/pjab1945.32.349.

(3) Nozoe, T.; Seto, S.; Matsumura, S.; Murase, Y. The Synthesis of Azulene Derivatives from Troponoids. *Bull. Chem. Soc. Jpn.* **1962**, *35* (7), 1179–1188. https://doi.org/10.1246/bcsj.35.1179.

(4) Shoji, T.; Maruyama, A.; Yamamoto, A.; Fujiwara, Y.; Ito, S.; Okujima, T.; Morita, N. Synthesis of 2,2'-Diamino-1,1'-Biazulenes by the Copper-Catalyzed Homocoupling Reaction of 2-Aminoazulenes. *Chem. Lett.* **2014**, *43* (7), 1122–1124. https://doi.org/10.1246/cl.140333.

(5) Vierle, M.; Zhang, Y.; Santos, A. M.; Köhler, K.; Haeßner, C.; Herdtweck, E.; Bohnenpoll, M.; Nuyken, O.; Kühn, F. E. Solvent-Ligated Manganese(II) Complexes for the Homopolymerization of Isobutene and the Copolymerization of Isobutene and Isoprene. *Chem. Eur. J.* **2004**, *10* (24), 6323–6332. https://doi.org/10.1002/chem.200400446.

(6) Kim, E. H.; Lee, H. M.; Jeong, M. S.; Ryu, J. Y.; Lee, J.; Lee, B. Y. Methylaluminoxane-Free Chromium Catalytic System for Ethylene Tetramerization. *ACS Omega* **2017**, *2* (3), 765–773. https://doi.org/10.1021/acsomega.6b00506.

(7) Lin, Y.-S.; Li, G.-D.; Mao, S.-P.; Chai, J.-D. Long-Range Corrected Hybrid Density Functionals with Improved Dispersion Corrections. *J. Chem. Theory Comput.* **2013**, *9* (1), 263–272. https://doi.org/10.1021/ct300715s.

(8) Weigend, F.; Ahlrichs, R. Balanced Basis Sets of Split Valence, Triple Zeta Valence and Quadruple Zeta Valence Quality for H to Rn: Design and Assessment of Accuracy. *Phys. Chem. Chem. Phys.* **2005**, *7* (18), 3297–3305. https://doi.org/10.1039/b508541a.

(9) Weigend, F. Accurate Coulomb-Fitting Basis Sets for H to Rn. *Phys. Chem. Chem. Phys.* **2006**, *8* (9), 1057–1065. https://doi.org/10.1039/b515623h.

(10) Kozuch, S.; Martin, J. M. L. DSD-PBEP86: In Search of the Best Double-Hybrid DFT with Spin-Component Scaled MP2 and Dispersion Corrections. *Phys. Chem. Chem. Phys.* **2011**, *13* (45), 20104–20107. https://doi.org/10.1039/c1cp22592h.

(11) Grimme, S.; Antony, J.; Ehrlich, S.; Krieg, H. A Consistent and Accurate Ab Initio Parametrization of Density Functional Dispersion Correction (DFT-D) for the 94 Elements H-Pu. *J. Chem. Phys.* **2010**, *132* (15), 154104. https://doi.org/10.1063/1.3382344.

(12) Barone, V.; Cossi, M. Quantum Calculation of Molecular Energies and Energy Gradients in Solution by a Conductor Solvent Model. *J. Phys. Chem.* **1998**, *102* (11), 1995–2001. https://doi.org/10.1021/jp9716997.

(13) Ásgeirsson, V.; Birgisson, B. O.; Bjornsson, R.; Becker, U.; Neese, F.; Riplinger, C.; Jónsson, H. Nudged Elastic Band Method for Molecular Reactions Using Energy-Weighted Springs Combined with Eigenvector Following. *J. Chem. Theory Comput.* **2021**, *17* (8), 4929–4945. https://doi.org/10.1021/acs.jctc.1c00462.

(14) Riplinger, C.; Neese, F. An Efficient and near Linear Scaling Pair Natural Orbital Based Local Coupled Cluster Method. *J. Chem. Phys.* **2013**, *138* (3), 034106. https://doi.org/10.1063/1.4773581.

(15) Casanova-Páez, M.; Dardis, M. B.; Goerigk, L. ΩB2PLYP and ΩB2GPPLYP: The First Two Double-Hybrid Density Functionals with Long-Range Correction Optimized for Excitation Energies. *J. Chem. Theory Comput.* **2019**, *15* (9), 4735–4744. https://doi.org/10.1021/acs.jctc.9b00013.

(16) Becke, A. D. A New Mixing of Hartree–Fock and Local Density-functional Theories. *J. Chem. Phys.* **1993**, *98* (2), 1372–1377. https://doi.org/10.1063/1.464304.

(17) Spartan'18. Wavefunction Inc.: Irvine.

(18) Frisch, M. J.; Trucks, G. W.; Schlegel, H. B.; Scuseria, G. E.; Robb, M. A.; Cheeseman, J. R.; Scalmani, G.; Barone, V.; Petersson, G. A.; Nakatsuji, H.; Li, X.; Caricato, M.; Marenich, A. V.; Bloino, J.; Janesko, B. G.; Gomperts, R.; Mennucci, B.; Hratchian, H. P.; Ortiz, J. V.; Izmaylov, A. F.; Sonnenberg, J. L.; Williams-Young, D.; Ding, F.; Lipparini, F.; Egidi, F.; Goings, J.; Peng, B.; Petrone, A.; Henderson, T.; Ranasinghe, D.; Zakrzewski, V. G.; Gao, J.; Rega, N.; Zheng, G.; Liang, W.; Hada, M.; Ehara, M.; Toyota, K.; Fukuda, R.; Hasegawa, J.; Ishida, M.; Nakajima, T.; Honda, Y.; Kitao, O.; Nakai, H.; Vreven, T.; Throssell, K.; Montgomery, Jr.; Peralta, J. E.; Ogliaro, F.; Bearpark, M. J.; Heyd, J. J.; Brothers, E. N.; Kudin, K. N.; Staroverov, V. N.; Keith, T. A.; Kobayashi, R.; Normand, J.; Raghavachari, K.; Rendell, A. P.; Burant, J. C.; Iyengar, S. S.; Tomasi, J.; Cossi, M.; Millam, J. M.; Klene, M.; Adamo, C.; Cammi, R.; Ochterski, J. W.; Martin, R. L.; Morokuma, K.; Farkas, O.; Foresman, J. B.; Fox, D. J. *Gaussian'16 Revision C.01*; Gaussian Inc.: Wallingford CT.

(19) Maglic, J. B.; Lavendomme, R. MoloVol: An Easy-to-Use Program for Analyzing Cavities, Volumes and Surface Areas of Chemical Structures. *J. Appl. Crystallogr.* **2022**, *55* (Pt. 4), 1033–1044. https://doi.org/10.1107/s1600576722004988.

(20) Sheldrick, G. M. SHELXT – Integrated Space-Group and Crystal-Structure Determination. *Acta Crystallogr., Sect A: Found. Adv.* **2015**, *71* (1), 3–8. https://doi.org/10.1107/s2053273314026370.

(21) Sheldrick, G. M. Crystal Structure Refinement with SHELXL. *Acta Crystallogr., Sect. C: Struct. Chem.* **2015**, *71* (1), 3–8. https://doi.org/10.1107/s2053229614024218.

(22) Hübschle, C. B.; Sheldrick, G. M.; Dittrich, B. ShelXle: A Qt Graphical User Interface for SHELXL. *J. Appl. Crystallogr.* **2011**, *44* (Pt 6), 1281–1284. https://doi.org/10.1107/s0021889811043202.

(23) Ursby, T.; Åhnberg, K.; Appio, R.; Aurelius, O.; Barczyk, A.; Bartalesi, A.; Bjelčić, M.; Bolmsten, F.; Cerenius, Y.; Doak, R. B.; Eguiraun, M.; Eriksson, T.; Friel, R. J.; Gorgisyan, I.; Gross, A.; Haghighat, V.; Hennies, F.; Jagudin, E.; Jensen, B. N.; Jeppsson, T.; Kloos, M.; Lidon-Simon, J.; Lima, G. M. A. de; Lizatovic, R.; Lundin, M.; Milan-Otero, A.; Milas, M.; Nan, J.; Nardella, A.; Rosborg, A.; Shilova, A.; Shoeman, R. L.; Siewert, F.; Sondhauss, P.; Talibov, V. O.; Tarawneh, H.; Thånell, J.; Thunnissen, M.; Unge, J.; Ward, C.; Gonzalez, A.; Mueller, U. BioMAX – the First Macromolecular Crystallography Beamline at MAX IV Laboratory. *J. Synchrotron Radiat.* **2020**, *27* (5), 1415–1429. https://doi.org/10.1107/s1600577520008723.

(24) Thorn, A.; Dittrich, B.; Sheldrick, G. M. Enhanced Rigid-Bond Restraints. *Acta Crystallogr., Sect A: Found. Adv.* **2012**, *68* (4), 448–451. https://doi.org/10.1107/s0108767312014535.

(25) Spek, A. L. PLATON SQUEEZE: A Tool for the Calculation of the Disordered Solvent Contribution to the Calculated Structure Factors. *Acta. Crystallogr., Sect. C: Struct. Chem.* **2015**, *71* (1), 9–18. https://doi.org/10.1107/s2053229614024929.

(26) Spek, A. L. Structure Validation in Chemical Crystallography. *Acta Crystallogr., Sect. D: Biol. Cryst.* **2009**, *65* (Pt 2), 148–155. https://doi.org/10.1107/s090744490804362x.

(27) Ursby, T.; Åhnberg, K.; Appio, R.; Aurelius, O.; Barczyk, A.; Bartalesi, A.; Bjelčić, M.; Bolmsten, F.; Cerenius, Y.; Doak, R. B.; Eguiraun, M.; Eriksson, T.; Friel, R. J.; Gorgisyan, I.; Gross, A.; Haghighat, V.; Hennies, F.; Jagudin, E.; Jensen, B. N.; Jeppsson, T.; Kloos, M.; Lidon-Simon, J.; Lima, G. M. A. de; Lizatovic, R.; Lundin, M.; Milan-Otero, A.; Milas, M.; Nan, J.; Nardella, A.; Rosborg, A.; Shilova, A.; Shoeman, R. L.; Siewert, F.; Sondhauss, P.; Talibov, V. O.; Tarawneh, H.; Thånell, J.; Thunnissen, M.; Unge, J.; Ward, C.; Gonzalez, A.; Mueller, U. BioMAX – the First Macromolecular Crystallography Beamline at MAX IV Laboratory. *J. Synchrotron Radiat.* **2020**, *27* (Pt 5), 1415–1429. https://doi.org/10.1107/s1600577520008723.

(28) Mueller, U.; Thunnissen, M.; Nan, J.; Eguiraun, M.; Bolmsten, F.; Milàn-Otero, A.; Guijarro, M.; Oscarsson, M.; Sanctis, D. de; Leonard, G. MXCuBE3: A New Era of MX-Beamline Control Begins. *Synchrotron Radiat. N.* **2017**, *30* (1), 22–27. https://doi.org/10.1080/08940886.2017.1267564.

(29) Delagenière, S.; Brenchereau, P.; Launer, L.; Ashton, A. W.; Leal, R.; Veyrier, S.; Gabadinho, J.; Gordon, E. J.; Jones, S. D.; Levik, K. E.; McSweeney, S. M.; Monaco, S.; Nanao, M.; Spruce, D.; Svensson, O.; Walsh, M. A.; Leonard, G. A. ISPyB: An Information Management System for Synchrotron Macromolecular Crystallography. *Bioinformatics* **2011**, *27* (22), 3186–3192. https://doi.org/10.1093/bioinformatics/btr535.

(30) Vonrhein, C.; Flensburg, C.; Keller, P.; Sharff, A.; Smart, O.; Paciorek, W.; Womack, T.; Bricogne, G. Data Processing and Analysis with the AutoPROC Toolbox. *Acta Crystallogr., Sect. D: Biol. Cryst* **2011**, *67* (4), 293–302. https://doi.org/10.1107/s0907444911007773.

(31) Kabsch, W. Integration, Scaling, Space-Group Assignment and Post-Refinement. *Acta Crystallogr Sect D Biological Crystallogr* **2010**, *66* (2), 133–144. https://doi.org/10.1107/s0907444909047374.

(32) Chen, A.; Yen, H.; Kuo, Y.; Chen, W. Asymmetric Synthesis and Characterization of Chiral 2,2'-Diamino-3,3'-diethoxycarbonyl-8,8'-diphenyl-1,1'-biazulene. *Synth. Commun.* **2007**, *37* (17), 2975–2987. https://doi.org/10.1080/00397910701473374.
